# Combining a Genetically Engineered Oxidase with Hydrogen‐Bonded Organic Frameworks (HOFs) for Highly Efficient Biocomposites

**DOI:** 10.1002/anie.202117345

**Published:** 2022-02-24

**Authors:** Peter Wied, Francesco Carraro, Juan M. Bolivar, Christian J. Doonan, Paolo Falcaro, Bernd Nidetzky

**Affiliations:** ^1^ Institute of Biotechnology and Biochemical Engineering Graz University of Technology Petersgasse 12/1 8010 Graz Austria; ^2^ Institute of Physical and Theoretical Chemistry Graz University of Technology Stremayrgasse 9/Z2 8010 Graz Austria; ^3^ Department of Chemistry The University of Adelaide Adelaide South Australia 5005 Australia

**Keywords:** Biocatalysis, Hydrogen-Bonded Organic Frameworks, Immobilization, Metal–Organic Frameworks, Porous Carrier

## Abstract

Enzymes incorporated into hydrogen‐bonded organic frameworks (HOFs) via bottom‐up synthesis are promising biocomposites for applications in catalysis and sensing. Here, we explored synthetic incorporation of d‐amino acid oxidase (DAAO) with the metal‐free tetraamidine/tetracarboxylate‐based BioHOF‐1 in water. N‐terminal enzyme fusion with the positively charged module Z_basic2_ strongly boosted the loading (2.5‐fold; ≈500 mg enzyme g_material_^−1^) and the specific activity (6.5‐fold; 23 U mg^−1^). The DAAO@BioHOF‐1 composites showed superior activity with respect to every reported carrier for the same enzyme and excellent stability during catalyst recycling. Further, extension to other enzymes, including cytochrome P450 BM3 (used in the production of high‐value oxyfunctionalized compounds), points to the versatility of genetic engineering as a strategy for the preparation of biohybrid systems with unprecedented properties.

## Graphical abstract

Immobilization of d‐amino acid oxidase in crystalline frameworks at high protein loading is reported. A biocomposite of hydrogen‐bonded organic framework (BioHOF‐1) shows excellent retention of activity. Fusion to the positively charged module Z_basic2_ (Z‐DAAO) promotes incorporation of the active enzyme. Protein engineering can facilitate development of framework‐based enzyme composites, as shown with three further examples of industrial enzymes.

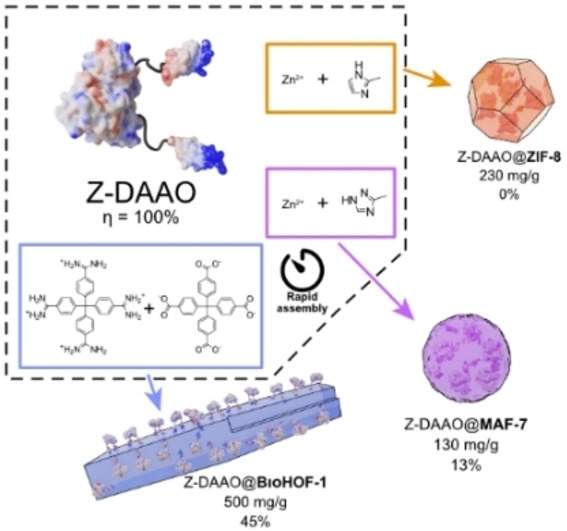

## Introduction

The application of enzymes to modern industrial processes[^1^, ^2^, ^3^] and therapeutics[^4^, ^5^] demands progress in research aimed at tailoring enzyme functionality and developing methodologies for their integration into devices.[^6^, ^7^, ^8^, ^9^, ^10^] Like most non‐fibrous proteins, enzymes are inherently fragile ex situ, hindering their use, for example, in commercial catalysis where recyclability is desired.[^11^, ^12^, ^13^] Thus, immobilization on, or within, solids is employed as a strategy to enhance enzyme stability in catalysis, biomedical science and biosensing applications.[^6^, ^8^, ^14^, ^15^, ^16^, ^17^, ^18^, ^19^, ^20^, ^21^] For example, infiltration into preformed porous materials (e. g., silica, organic polymers) is well known for enzyme immobilization.[^20^, ^22^, ^23^] A key challenge in the synthesis of such biocomposites is to achieve high enzyme loading on the surface of the solid while retaining the biological activity.[^21^, ^24^, ^25^, ^26^, ^27^]

**Figure 1:**
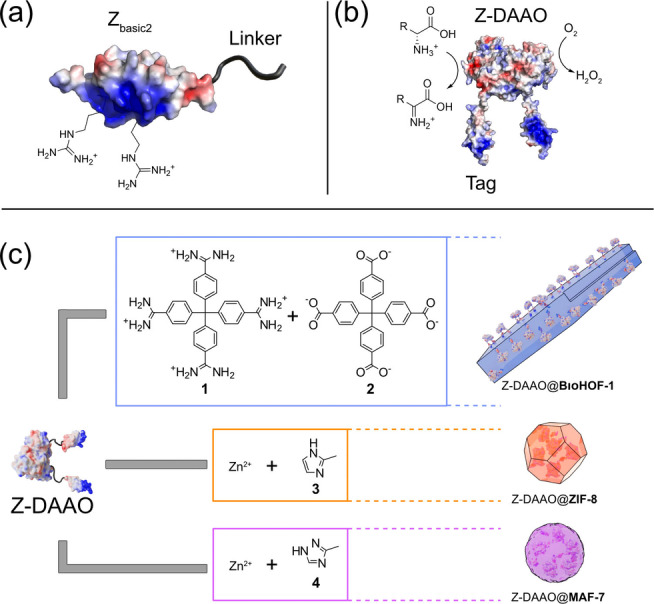
Strategy of biocomposite development. a) Z_basic2_ binding module and clustered arginine residues. A structural model of d‐amino acid oxidase from *Rhodotorula toruloides* and the fused Z_basic2_ binding module was created using The PyMOL Molecular Graphics System, Version 2.6 Schrödinger, LLC. Surface charge visualization was calculated using APBS software.^[59]^ b) Schematic representation of Z‐DAAO dimer catalyzing the deamination of d‐amino acids. c) Precursors used for the one‐pot and water‐based synthesis of each biocomposite (Z‐DAAO@BioHOF‐1, Z‐DAAO@ZIF‐8, and Z‐DAAO@MAF‐7).

Porous crystalline frameworks such as metal‐organic frameworks (MOFs) and hydrogen‐bonded organic frameworks (HOFs)[^28^, ^29^] are a class of solids assembled in a modular fashion and thus allow for precise control of their structures through the judicious choice of the building blocks. Whereas MOFs consist of metal nodes and organic linkers, HOFs are assembled through hydrogen bonding of organic components.[^30^, ^31^, ^32^, ^33^, ^34^] These materials offer unique opportunities to promote enzyme immobilization beyond the limits of traditional carrier‐based approaches[^8^, ^35^, ^36^, ^37^, ^38^, ^39^] as their bottom‐up synthesis can be carried out under enzyme‐compatible incubation conditions. Thus, biocomposites that preserve the active enzyme into the porous solids framework can be realized.[^8^, ^36^, ^37^] Indeed, MOF‐ and HOF‐based composites have been reported for a number of enzymes and several show promise as biocatalysts with high specific activity, stability and recyclability.[^36^, ^37^, ^40^, ^41^] Recent seminal studies in HOF biocomposites have shown very high protein loading.^[41]^ In this case the protein surface was chemically modified to enhance its positive charge, yielding favorable interactions with the carboxylate‐based HOF building blocks.[^41^, ^42^] Although this approach resulted in high enzyme loading, protein surface functionalization protocols require multistep procedures,[^41^, ^43^, ^44^] depend on the specific sequence of amino acids and their accessibility,^[45]^ and can influence the native bioactivity.^[46]^ A strategy that has been overlooked to increase protein‐framework interactions is protein engineering, where progress in both molecular biotechnology and DNA manipulation has enabled a straightforward and cost‐effective expression of fused protein systems.[^47^, ^48^, ^49^] For example, enzymes can be expressed with arginine‐rich mini‐proteins (modules) connected by a polypeptide chain (Z_basic2_ made of 58 amino acid; 7 kDa) to improve non‐covalent immobilization on inorganic substrates.^[50]^ We hypothesize that such positive surface charge enrichment via Z_basic2_ modules would enhance the protein immobilization in HOFs (Figure 1a).

**Figure 2:**
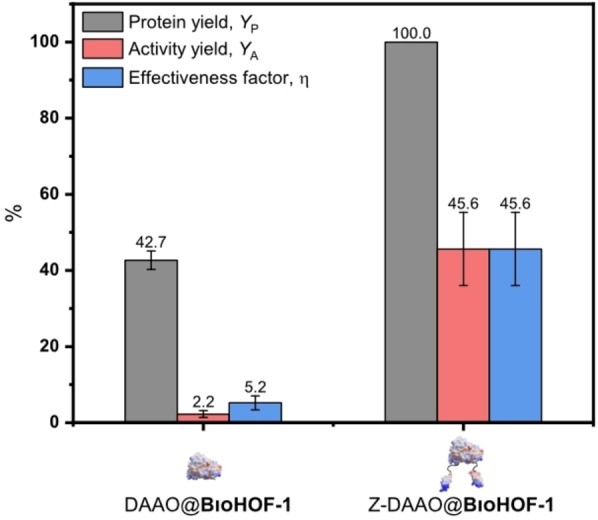
Immobilization performance of the HOF biocomposite prepared with the native enzyme (DAAO@BioHOF‐1) and with the Z_basic2_ module engineered enzyme (Z‐DAAO@BioHOF‐1). Protein yield (*Y*_P_), activity yield (*Y*_A_), and effectiveness factor (*η*) are given in %.

Here, we report a detailed study of biocomposites prepared via mixing Z_basic2_ functionalized d‐amino acid oxidase (Z‐DAAO; Figure 1b) and BioHOF1 precursors (tetraamidinium and tetracarboxylate linkers; Figure 1c, 1, 2) in water. DAAO was selected due its broad relevance in industrial bio‐catalysis[^15^, ^51^] and due to its potential applications in analytics and medicine.[^52^, ^53^] For comparison, Z‐DAAO biocomposites were prepared from two MOF materials that are known to immobilize enzymes via an analogous *one‐pot* approach; Zeolitic Imidazolate Framework 8^[54]^ (ZIF‐8, composed of Zn^2+^ and 2‐methylimidazole, 3) and a structurally analogous but more hydrophilic material, Metal Azolate Framework 7^[55]^ (MAF‐7, composed of Zn^2+^ and 3‐methyl‐1,2,4‐triazole, 4; Figure 1c). This comparison allowed us to benchmark the performance characteristics of the HOF‐based biocomposite to those of the well‐studied ZIF‐based analogues. In summary, we show that Z_basic2_ functionalization enhances DAAO immobilization in/on BioHOF‐1 and that the resulting enzymatic activity of the Z‐DAAO@BioHOF‐1 significantly outperforms Z‐DAAO@ZIF‐8, Z‐DAAO@MAF‐7 and all previously reported DAAO composites. The versatility of this strategy was explored by synthesizing BioHOF‐1‐based biocomposites using three additional, industrially relevant, enzymes modified with the Z_basic2_ module; cytochrome P450 BM3,^[56]^ sucrose phosphorylase BlSP,^[57]^ and phosphatase HAD4.^[58]^ For each enzyme, the specific activity measured for the respective biocomposite was superior to literature reported values (Table S1). Thus, we envision that protein engineering will offer new opportunities for the synthesis of efficient HOF‐based composites for enzyme applications.

## Results and Discussion

Native DAAO[^60^, ^61^] from *Trigonopsis variabilis* (78 kDa, homodimer) and DAAO fused with the cationic binding module Z_basic2_ (7 kDa),^[62]^ here referred to as Z‐DAAO, were purified from an *Escherichia coli* expression culture (see Supporting Information_I2). The addition of the positively charged Z_basic2_ module confers affinity for binding to negatively charged surfaces and has been employed to facilitate immobilization on solid supports (e. g., porous silica) and to control biomolecular orientation.[^62^, ^63^, ^64^, ^65^, ^66^] In this study we tested both native DAAO and Z‐DAAO for the *one‐pot* preparation of HOF and ZIF‐based biocomposites, here denoted as DAAO@BioHOF‐1, Z‐DAAO@BioHOF‐1.

Addition of DAAO and Z‐DAAO (1.0 mg mL^−1^), respectively, to a solution of BioHOF‐1 precursors (tetraamidinium and tetracarboxylate linkers), yielded the rapid formation of a solid precipitate. Close inspection of the precipitate via optical microscopy revealed that the precipitate was comprised of high aspect ratio particles. We note such particle morphology is typical of BioHOF‐1 composites (Figure S8).^[40]^ The samples were then centrifuged and the enzyme loading and activity were determined. For DAAO@BioHOF‐1, only 43 % of the protein was immobilized (see protein yield, *Y*_P_, aka encapsulation efficiency EE, Figure 2) whereas fusion of the Z_basic2_ module in Z‐DAAO engendered a *Y*_P_ of 100 %. Enzyme activity was measured using the DAAO assay (see Supporting Information_I10). As control experiment, we performed the DAAO assay on each of the supports and each showed zero activity in the absence of the enzyme. Notably, when compared to DAAO immobilized in BioHOF‐1, Z‐DAAO@BioHOF‐1 resulted in a 20‐fold higher activity retention (see activity yield, *Y*_A_, Supporting Information_I13—the activity improved from 2 % for DAAO@BioHOF‐1 to 46 % for Z‐DAAO@BioHOF‐1).^[8]^ Compared to the free enzyme, the activity of the bound enzyme of Z‐DAAO@BioHOF‐1 is 6.5‐fold the activity of DAAO@BioHOF‐1 (effectiveness factor *η*, Supporting Information_I13). These results suggest that the Z_basic2_ module is primarily responsible for the high enzyme loading and activity of observed for the Z‐DAAO@BioHOF‐1 composite.

**Figure 3:**
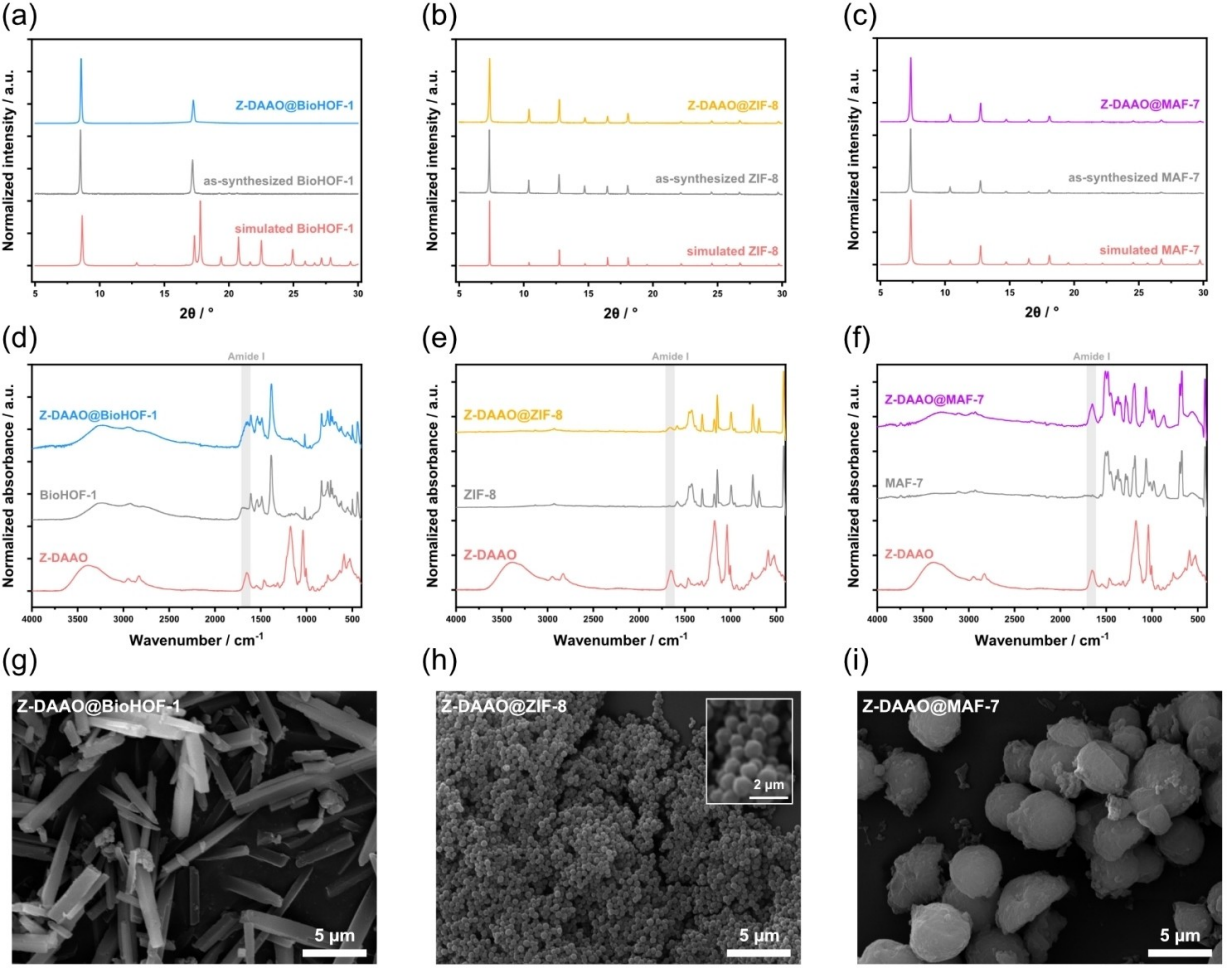
Material characterization of Z‐DAAO@BioHOF‐1, Z‐DAAO@ZIF‐8, and Z‐DAAO@MAF‐7 with an initial Z‐DAAO concentration of 1 mg mL^−1^ during synthesis after optimization of the synthesis to obtain each material at the correct topology. a)–c) PXRD patterns including simulated PXRD patterns of each material (red), each material without biocatalyst (grey), and Z‐DAAO@HOF/MOF. d)–f) ATR‐FTIR spectra of each material with/without biocatalyst. g)–i) SEM images of the obtained biocomposites with inset zoom of Z‐DAAO@ZIF‐8

The positive charge on Z_basic2_ derives from several Arg residues clustered on one side of the module's three‐helical bundle structure (Figure 1a).^[66]^ The Arg guanidine group is chemically similar to the amidine groups of the cationic building block of the BioHOF‐1. Thus we hypothesize that Z‐DAAO incorporation into the nascent BioHOF‐1 composite may involve a multivalent process whereby Z_basic2_ guanidine moieties bind to the exposed carboxylate groups of the HOF in place of tetraamidinium building units.^[41]^ This idea is in good agreement with examples of HOF composites that report superior *Y*_P_ for amino‐modified model proteins (i. e., BSA and GFP), compared with pristine and carboxyl‐modified proteins.[^41^, ^67^] The structures of the DAAO biocomposites were examined by X‐ray diffraction (XRD), which revealed that the diffraction patterns of DAAO@BioHOF‐1 and Z‐DAAO@BioHOF‐1 were analogous to that of as‐synthesized BioHOF‐1 (see Figure S9 and Figure 3a).^[40]^

Next, we prepared Z‐DAAO composites of ZIF‐8 and, the isostructural but more hydrophilic, MAF‐7 to benchmark the performance of Z‐DAAO@BioHOF‐1 to other porous biocomposites that can be prepared via one‐pot methods in water. Employing reported protocols^[68]^ for the synthesis of the ZIF‐8 and MAF‐7 biocomposites yielded Z‐DAAO@ZIF‐L^[69]^ an amorphous product, respectively (Figure S10 and S11). However, by modifying the synthesis conditions (e. g., metal‐to‐ligand ratio and concentration of ammonia), biocomposites of sodalite (*sod*) topology were obtained (see S12–S15, and Figure 3b, c). The sod topology (flexible framework with pore window of 3.4 Å) ensures accessible porosity and maximizes mass diffusion.[^54^, ^70^, ^71^]

The chemical composition of each biocomposite was examined by Fourier‐transformed infrared (FTIR) spectroscopy (see Figure 3d–f). Z‐DAAO@BioHOF‐1, Z‐DAAO@ZIF‐8 and Z‐DAAO@MAF‐7 composites each showed an absorbance band in the protein amide I region (1700–1600 cm^−1^) that was absent in pure framework and increased with enzyme loading, thus confirming the presence of the enzyme (Figure S16–S18).^[72]^

However, for Z‐DAAO@ZIF‐8, a bifurcation in the amide I vibrational band (1650 cm^−1^) is observed (Figure S19). This suggests the secondary structure of the enzyme is modified upon composite formation.[^73^, ^74^, ^75^, ^76^, ^77^] Additionally, confocal laser scanning microscopy (CLSM) images of the porous crystals grown in presence of Z‐DAAO, tagged with Invitrogen Alexa Fluor™ 647, showed emission of a homogeneous fluorescent signal that is consistent with encapsulated enzyme (Figure S20–S22).

The particle size and morphology of the biocomposites were examined using scanning electron microscopy (SEM, see particle size analysis Figure S23–S32). Figure 3g shows that Z‐DAAO@BioHOF‐1 formed needle‐like crystals of ca. 7 μm with squared cross section of 700 nm in width, Z‐DAAO@ZIF‐8 exhibited a rhombic dodecahedral morphology (Figure 3h) with average particle size of ca. 500 nm and Z‐DAAO@MAF‐7 formed spherical particles (Figure 3i) of ca. 4.5 μm that presumably formed due to crystal inter‐growth. We note that the presence of the enzyme in the synthesis did not influence the particle morphology of the different frameworks (Figure S23–S25), however, a significant change in average particle size was noted only for pure MAF‐7 (ca. 30 % decrease).

To further characterize the crystalline frameworks, the ζ‐potential of each material and biocomposite was measured (Figure S33). BioHOF‐1, ZIF‐8 and MAF‐7 showed positive ζ‐potential charges, +27.7 mV, +16.0 mV and +26.3 mV, respectively. The ζ‐potential was decreased in the biocomposites compared to the corresponding plain materials in the order Z‐DAAO@MAF‐7 (Δ8.0 mV), Z‐DAAO@ZIF‐8 (Δ7.1 mV) and Z‐DAAO@BioHOF‐1 (Δ2.0 mV). As Z‐DAAO has a negative ζ‐potential charge of −9.5 mV, the ζ‐potential values of the biocomposites can be explained by the protein immobilization into the porous particles.

**Figure 4:**
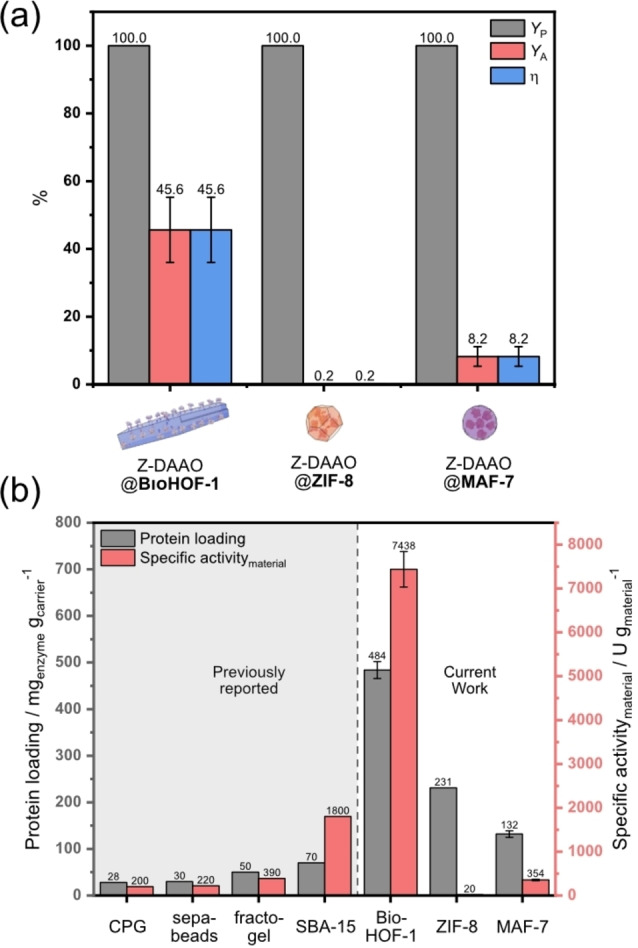
a) The immobilization performance of Z‐DAAO@BioHOF‐1, Z‐DAAO@ZIF‐8, and Z‐DAAO@MAF‐7 prepared with an initial Z‐DAAO concentration of 1 mg mL^−1^ during the immobilization (values calculated averaging at least 3 different batches from biological replicates). Protein yield (*Y*_P_), activity yield (*Y*_A_), and effectiveness factor (*η*) are given in %. b) Protein loading and specific activity of DAAO@ZIF‐8, DAAO@MAF‐7 and DAAO@BioHOF‐1 (current work) compared to previously reported results of immobilized DAAO (grey): controlled pore glass (CPG),^[63]^ sepabeads,^[62]^ fractogel^[62]^ and ordered mesoporous silica (SBA‐15).^[64]^

Next, the immobilization performance at varying initial Z‐DAAO concentrations, during the biocomposite synthesis, was assessed. The framework integrity at all Z‐DAAO loadings was confirmed by FTIR and XRD analyses (Figure S16–S18, S34–S36). Each of the biocomposites incorporated all of the Z‐DAAO (0.25–1.5 mg mL^−1^) from solution into the solid material (see *Y*_P_ Figure 4a, Figure S37). Given that the protein yield is 100 % for all three biocomposites *Y*_A_=*η*. The activity of the three materials varied significantly; Z‐DAAO@BIOHOF‐1 was highly active (*η*=45 %), while Z‐DAAO@ZIF‐8 was inactive, likely due to the hydrophobic nature of the framework.^[68]^ Z‐DAAO@MAF‐7 retained partial activity with *η*=8 % (Figure S38). This could be ascribed to the enhanced diffusion through defects in the MAF‐7 structure^[78]^ or possibly that the solid dissolved during the assay and the activity recorded may be the result of free enzyme (Figure S39).

Figure 4b compares the effective protein loading, considered a key parameter for bioreactor design, of the HOF and ZIF‐based biocomposites along with traditional materials employed for DAAO immobilization.[^79^, ^80^] The DAAO@BioHOF‐1 composite (0.55 g_enyzme_ g_material_^−1^) showed a higher effective loading than the two MOF materials and significantly exceeded the limits of traditional materials (≈0.1 g_enyzme_ g_material_^−1^). For example, Z‐DAAO@BioHOF‐1 is ca. 7‐fold higher than Z‐DAAO@SBA‐15.^[64]^ Furthermore, the exceptionally high loading for Z‐DAAO@BioHOF‐1 corresponded to the highest specific activity (ca. 7500 U g^−1^_material_). It is worth noting that enzymes of the O_2_ dependent class are challenging to process into highly active solid preparations.[^81^, ^82^, ^83^, ^84^]

**Figure 5:**
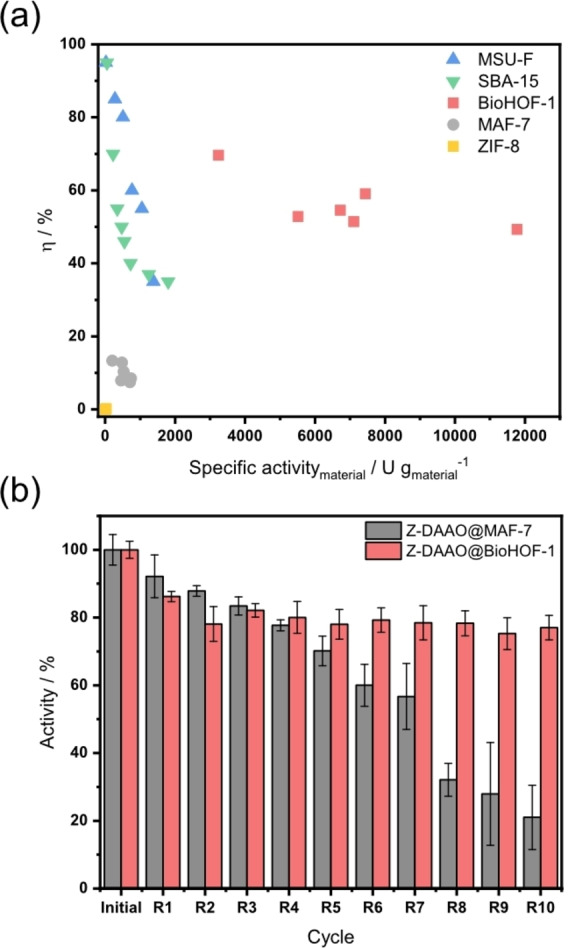
DAAO biocomposite characterization. a) Specific activity_material_ vs. effectiveness factor (*η*) of Z‐DAAO@BioHOF‐1 and Z‐DAAO@ZIF‐8 and Z‐DAAO@MAF‐7 compared to ultra large pore SBA‐15 silica^[64]^ and mesocellular silica foam MSU‐F.^[86]^ b) Recycling of DAAO@MAF‐7 and DAAO@BioHOF‐1. Activity after each cycle. The reaction was performed with 20 mg_wet weight_ mL^−1^ biocomposite, 10 mM d‐methionine and 20 mM HEPES (pH 8) at 30 °C after each cycle the biocomposite was separated by centrifugation and reused in a fresh reaction mixture.

For O_2_ dependent enzymes, *η* decreases sharply with increasing enzyme loading due to O_2_ depletion inside the porous catalyst.[^84^, ^85^, ^86^, ^87^] This can be seen by the decrease of *η* at increased specific activity_material_ for silica‐based support such as MSU‐F and SBA‐15 (see Figure 5a). Enzyme composites showing increased O_2_ activity due to better balance between reaction and diffusion are thus desirable.^[85]^ The crystalline porous frameworks are promising in this respect due to their large pore volume and accessible porosity. To this end we examined η vs. specific activity_material_ for the MOF and HOF‐based biocomposites. Figure 5a shows that Z‐and DAAO@BioHOF‐1 are highly active: 5.7‐fold more active than the best MOF biocomposite (Z‐DAAO@MAF‐7) with a measured specific activity of ≈24 U mg_enzyme_ (*η*≈45 %). Remarkably, compared to traditional porous silica (MSU‐F and SBA‐15)[^64^, ^86^] or MAF‐7, Z‐DAAO@BioHOF‐1 showed that *η* is preserved even at higher enzyme loading (Figure S40–S44). Indeed, the high *η* of the BioHOF‐1 composite (higher than ≈45 %) results in a record value of specific activity per gram of material: 6 times higher than the best previously reported value for mesoporous silica (SBA‐15) and ca. 12 times that of the hydrophilic MOF (MAF‐7). We posit that the record activity of Z‐DAAO@BioHOF‐1, can be attributed to the unique 1D (needle‐like) particle geometry of the composite.

In addition, the spatial distribution of immobilized enzymes in/on carriers may play a crucial role.[^88^, ^89^] To distinguish between the externally and internally bound enzyme, we used tryptic digestion to inactivate the Z‐DAAO. Substantial loss (>90 %) of Z‐DAAO@BioHOF‐1 activity on incubation with trypsin revealed that the major portion of the immobilized Z‐DAAO was protease‐accessible, hence at least partially exposed to external surface of the particles (Figure S45). Importantly, the performance characteristics of the Z‐DAAO@BioHOF‐1 composite could not be reproduced by a simple adsorption of the Z‐DAAO on a pre‐synthesized BioHOF‐1 framework (Figure S46). The enzyme binding to the solid was decreased strongly (>70 %) and the specific activity of the immobilized Z‐DAAO lowered to a similar degree (>70 %) as compared to Z‐DAAO@BioHOF‐1 synthesized via the *one‐pot* approach. Overall, the comparison between *η* for the adsorption of the Z‐DAAO on BioHOF‐1 and *one‐pot* preparations of DAAO@BioHOF‐1 and Z‐DAAO@BioHOF‐1 (see Figure 2 and Figure S46) suggests that for the *one‐pot* Z‐DAAO@BioHOF‐1 system the enzyme is partially embedded on the HOF surface.[^64^, ^90^] While this hypothesis requires further study beyond the current scope of this work, it is evident that the incorporation of the Z_basic2_ module via rational protein engineering is a promising strategy for the development of framework‐based enzyme composites.[^11^, ^91^, ^92^] Nevertheless, the notion of partially embedded DAAO is consistent with kinetic studies that show a *K*_M_ value for d‐Met ≈2.2‐fold lower in Z‐DAAO@BioHOF‐1 than soluble Z‐DAAO as this implies a catalytic reaction for the immobilized Z‐DAAO that is effectively unrestricted by diffusion (Table 1, Figure S47–S52). Slow diffusion into the solid catalyst would show as an increase in the apparent *K*_m_ (Figure S53).[^23^, ^84^, ^93^] Decrease in the *K*_M_ might be explained by a favorable partitioning of the somewhat hydrophobic d‐Met between the liquid phase and the solid surface of the composite.^[94]^ Remarkably, therefore, the catalytic efficiency (*k*_cat_/*K*_M_) of the Z‐DAAO@BioHOF‐1 composite is close to that of the soluble enzyme. Consistent with this interpretation, the d‐Met *K*_M_ of Z‐DAAO@MAF‐7 was comparable to that of the soluble enzyme suggesting that diffusional effects are negligible.

**Table 1:** Comparison of apparent kinetic parameters for free (DAAO) and immobilized DAAO (DAAO@MAF‐7 and DAAO@BioHOF‐1) for d‐methionine. At an initial Z‐DAAO concentration of 1 mg mL^−1^ during the immobilization.

Catalyst	*V*_max_ [μmol min^−1^ mg_enzyme_^−1^]	*k*_cat_ [s^−1^]	*K*_M_ [mM]	*k*_cat_/*K*_M_ [s^−1^ mM^−1^]
Z‐DAAO	50.1±0.8	40.6±0.7	1.26±0.11	16.1±2.9
Z‐DAAO@MAF‐7	3.2±0.01	2.6±0.0	1.96±0.08	0.7±0.0
Z‐DAAO@BioHOF‐1	16.3±0.1	13.2±0.0	0.56±0.03	11.8±1.2

The retention of enzyme activity in the Z‐DAAO@MAF‐7 composite and the loss of activity for Z‐DAAO@ZIF‐8 (Figure 4a) prompted further investigation. When incubated with the soluble Z‐DAAO for 1 h, the framework precursors of all three composites proved to be deleterious to enzyme activity (Figure S54). In particular Zn(NO_3_)_2_ (MAF‐7) resulted in a greater loss in activity than Zn(OAc)_2_ (ZIF‐8). However, such simple stability assays cannot reproduce the self‐assembly conditions that lead to rapid composite formation (≤5 min). Indeed, the evidence implies that reason for the inactive Z‐DAAO@ZIF‐8 is likely due to the microenvironment surrounding the enzyme in the solid composite. For example, FTIR analysis (Figure S19) shows a characteristic change in infrared band (band shift to higher wavenumber; single band→bifurcated band)[^73^, ^74^, ^75^, ^76^, ^77^] in the amide I region of the spectrum that is indicative of more significant denaturation in Z‐DAAO@ZIF‐8 compared to Z‐DAAO@MAF‐7 and the free enzyme.^[68]^

We analyzed the biological stability and the structural robustness of the Z‐DAAO composites via recycling experiments of the HOF‐based solid catalyst for d‐Met oxidation (Figure S55, S56) using surface aeration to supply O_2_. On the basis of specific activity of the framework‐bound enzyme, the Z‐DAAO@BioHOF‐1 showed excellent stability, with negligible loss of activity (ca. 20 %) over the first three reaction cycles (Figure 5b), followed by a plateau. It is worth noting that for recycling tests of immobilized enzymes a decrease in specific activity of about 25 % can be observed in the first cycle.^[95]^ In case of Z‐DAAO@BioHOF‐1, this may reflect that initially surface bound Z‐DAAO could be readily released from the material (for the general case, see ref [26]). Stability tests show that after 10 cycles the weight and crystallinity of Z‐DAAO@BioHOF‐1 were maintained (Figure S57a, S57b). However, the particle morphology showed increased surface roughness (Figure S57c, S57d). Similar stability was observed for Z‐DAAO@MAF‐7, however this biocomposite featured gradual decrease in specific activity to just ≈20 % remaining in the last cycle. Furthermore, these results show that the BioHOF‐1‐bound Z‐DAAO was significantly more stable and more active than the MAF‐bound Z‐DAAO.

To highlight the potential of Z‐DAAO@BioHOF‐1 for sensing and industrial biocatalysis, we examined d‐serine (a key metabolite of neural signaling in the brain)[^96^, ^97^] and cephalosporin C^[98]^ (Industrial production of the antibiotic precursor 7‐amino‐cephalosporanic acid involves immobilized DAAO for oxidation of cephalosporin C as the key first step of the bioprocess.^[98]^) as substrates for the immobilized enzyme (Figure S58). Indeed, Z‐DAAO@BioHOF‐1 showed great promise for use in these processes retaining excellent values of *η* for d‐serine (49 %) and cephalosporin C (91 %).

Lastly, the broad scope of the Z_basic2_ fusion approach to enzyme immobilization in BioHOF‐1 was demonstrated by synthesizing composites from three enzymes relevant to applied biocatalysis; cytochrome P450 BM3,[^56^, ^99^] sucrose phosphorylase BlSP,^[57]^ and phosphatase HAD4.^[58]^ Our data showed that loading of the Zenzyme (0.25–0.62 g_enzyme_ g_material_^−1^), was significantly enhanced (20–50 %) with respect to the corresponding reference His‐tagged enzyme (Figure S59). Further, all Z‐enzyme@BioHOF‐1 composites were active (Figure S60–S62) and showed a specific catalyst activity (U g_material_^−1^) surpassing the activity of representative immobilizations on porous carriers (Table S1). Therefore, BioHOF‐1 together with a rapidly evolving diversity of alternative HOF materials^[30]^ offers excellent scope for the development of Z‐enzyme composites.

## Conclusion

We presented a systematic evaluation of three crystalline, porous framework materials (BioHOF‐1, ZIF‐8 and MAF‐7) for the aqueous *one‐pot* immobilization of Z‐DAAO, without the use of organic co‐solvents (e. g. DMF[^41^, ^67^]). BioHOF‐1, a hydrogen‐bonded organic framework, shows outstanding performance with respect to enzyme loading and retention of activity, exceeding both ZIF‐8 and MAF‐7 as well as traditional immobilization carriers. Our results suggest that the surface‐binding module Z_basic2_ drives the active incorporation of the enzyme into the functional BioHOF‐1 composite. Studies of three further Z‐enzymes support the suggestion that modular approaches based on fusion proteins that involve Z_basic2_ as incorporator module could facilitate the enzyme biocomposite development. Despite the high loadings achieved, the Z‐DAAO catalyzes O_2_‐dependent transformations of industrial interest without evidence of diffusional restrictions and shows excellent stability and can be cycled with negligible loss of activity. The outstanding performance characteristics of Z‐DAAO@BioHOF‐1 demonstrate the potential scope for combining fusion proteins with BioHOF‐1 for the preparation of a new generation of highly efficient heterogeneous biocatalysts.

## Conflict of interest

The authors declare no conflict of interest.

1

## Supporting information

As a service to our authors and readers, this journal provides supporting information supplied by the authors. Such materials are peer reviewed and may be re‐organized for online delivery, but are not copy‐edited or typeset. Technical support issues arising from supporting information (other than missing files) should be addressed to the authors.

Supporting Information

## Data Availability

The data that support the findings of this study are available from the corresponding author upon reasonable request.

## References

[1] R. A. Sheldon , J. M. Woodley , Chem. Rev. 2018, 118, 801–838. 28876904 10.1021/acs.chemrev.7b00203

[2] E. L. Bell , W. Finnigan , S. P. France , A. P. Green , M. A. Hayes , L. J. Hepworth , S. L. Lovelock , H. Niikura , S. Osuna , E. Romero , K. S. Ryan , N. J. Turner , S. L. Flitsch , Nat. Rev. Methods Primers 2021, 1, 46.

[3] S. Wu , R. Snajdrova , J. C. Moore , K. Baldenius , U. T. Bornscheuer , Angew. Chem. Int. Ed. 2021, 60, 88–119; 10.1002/anie.202006648 PMC7818486 32558088

[4] M. Vellard , Curr. Opin. Biotechnol. 2003, 14, 444–450. 12943856 10.1016/s0958-1669(03)00092-2

[5] S. A. Farhadi , E. Bracho-Sanchez , S. L. Freeman , B. G. Keselowsky , G. A. Hudalla , Bioconjugate Chem. 2018, 29, 649–656. 10.1021/acs.bioconjchem.7b00719 PMC5993548 29285931

[6] R. A. Sheldon , A. Basso , D. Brady , Chem. Soc. Rev. 2021, 50, 5850–5862. 34027942 10.1039/d1cs00015b

[7] M. Li , N. T. Blum , J. Wu , J. Lin , P. Huang , Adv. Mater. 2021, 33, 2008438. 10.1002/adma.202008438 34197008

[8] W. Liang , P. Wied , F. Carraro , C. J. Sumby , B. Nidetzky , C.-K. Tsung , P. Falcaro , C. J. Doonan , Chem. Rev. 2021, 121, 1077–1129. 33439632 10.1021/acs.chemrev.0c01029

[9] I. S. Kucherenko , O. O. Soldatkin , D. Y. Kucherenko , O. V. Soldatkina , S. V. Dzyadevych , Nanoscale Adv. 2019, 1, 4560–4577. 10.1039/c9na00491b PMC9417062 36133111

[10] H. Chen , O. Simoska , K. Lim , M. Grattieri , M. Yuan , F. Dong , Y. S. Lee , K. Beaver , S. Weliwatte , E. M. Gaffney , S. D. Minteer , Chem. Rev. 2020, 120, 12903–12993. 33050699 10.1021/acs.chemrev.0c00472

[11] V. D. Jäger , R. Lamm , K. Küsters , G. Ölçücü , M. Oldiges , K.-E. Jaeger , J. Büchs , U. Krauss , Appl. Microbiol. Biotechnol. 2020, 104, 7313–7329. 32651598 10.1007/s00253-020-10760-3 PMC7413871

[12] G. Ölçücü , O. Klaus , K.-E. Jaeger , T. Drepper , U. Krauss , ACS Sustainable Chem. Eng. 2021, 9, 8919–8945.

[13] U. Roessl , J. Nahálka , B. Nidetzky , Biotechnol. Lett. 2010, 32, 341–350. 19943180 10.1007/s10529-009-0173-4

[14] P. T. Anastas , R. H. Crabtree , Green Catalysis, Vol. 3, Wiley, Hoboken, 2014.

[15] K. Buchholz , V. Kasche , U. T. Bornscheuer , Biocatalysts and Enzyme Technology, Wiley, Hoboken, 2012.

[16] Y. Dai , C. C. Liu , Angew. Chem. Int. Ed. 2019, 58, 12355–12368; 10.1002/anie.201901879 30990933

[17] H. H. Nguyen , S. H. Lee , U. J. Lee , C. D. Fermin , M. Kim , Materials 2019, 12, 121. 10.3390/ma12010121 PMC6337536 30609693

[18] P. Pinyou , V. Blay , L. M. Muresan , T. Noguer , Mater. Horiz. 2019, 6, 1336–1358.

[19] Immobilization of Enzymes and Cells: Methods and Protocols (Eds.: J. M. Guisan , J. M. Bolivar , F. López-Gallego , J. Rocha-Martín ), Springer US, New York, 2020. 10.1007/978-1-0716-0215-7_33 32193833

[20] J. Zdarta , A. S. Meyer , T. Jesionowski , M. Pinelo , Catalysts 2018, 8, 92.

[21] M. Romero-Fernández , F. Paradisi , Curr. Opin. Chem. Biol. 2020, 55, 1–8. 31865258 10.1016/j.cbpa.2019.11.008

[22] S. Cantone , V. Ferrario , L. Corici , C. Ebert , D. Fattor , P. Spizzo , L. Gardossi , Chem. Soc. Rev. 2013, 42, 6262–6276. 23525282 10.1039/c3cs35464d

[23] J. M. Bolivar , I. Eisl , B. Nidetzky , Catal. Today 2016, 259, 66–80.

[24] J. M. Bolivar , B. Nidetzky , Biochim. Biophys. Acta Proteins Proteomics 2020, 1868, 140333. 31778816 10.1016/j.bbapap.2019.140333

[25] C. Garcia-Galan , Á. Berenguer-Murcia , R. Fernandez-Lafuente , R. C. Rodrigues , Adv. Synth. Catal. 2011, 353, 2885–2904.

[26] D. Faulón Marruecos , D. K. Schwartz , J. L. Kaar , Curr. Opin. Colloid Interface Sci. 2018, 38, 45–55.

[27] N. Carlsson , H. Gustafsson , C. Thörn , L. Olsson , K. Holmberg , B. Åkerman , Adv. Colloid Interface Sci. 2014, 205, 339–360. 24112562 10.1016/j.cis.2013.08.010

[28] S. R. Batten , N. R. Champness , X.-M. Chen , J. Garcia-Martinez , S. Kitagawa , L. Öhrström , M. O'Keeffe , M. P. Suh , J. Reedijk , Pure Appl. Chem. 2013, 85, 1715–1724.

[29] I. Hisaki , C. Xin , K. Takahashi , T. Nakamura , Angew. Chem. Int. Ed. 2019, 58, 11160–11170; 10.1002/anie.201902147 30891889

[30] S. A. Boer , M. Morshedi , A. Tarzia , C. J. Doonan , N. G. White , Chem. Eur. J. 2019, 25, 10006–10012. 31267583 10.1002/chem.201902117

[31] P. Li , M. R. Ryder , J. F. Stoddart , Acc. Mater. Res. 2020, 1, 77–87.

[32] T. Adachi , M. D. Ward , Acc. Chem. Res. 2016, 49, 2669–2679. 27689535 10.1021/acs.accounts.6b00360

[33] M. A. Little , A. I. Cooper , Adv. Funct. Mater. 2020, 30, 1909842.

[34] B. Wang , R.-B. Lin , Z. Zhang , S. Xiang , B. Chen , J. Am. Chem. Soc. 2020, 142, 14399–14416. 32786796 10.1021/jacs.0c06473

[35] R. J. Drout , L. Robison , O. K. Farha , Coord. Chem. Rev. 2019, 381, 151–160.

[36] S. Huang , X. Kou , J. Shen , G. Chen , G. Ouyang , Angew. Chem. Int. Ed. 2020, 59, 8786–8798; 10.1002/anie.201916474 31901003

[37] S. Liang , X.-L. Wu , J. Xiong , M.-H. Zong , W.-Y. Lou , Coord. Chem. Rev. 2020, 406, 213149.

[38] S. S. Nadar , L. Vaidya , V. K. Rathod , Int. J. Biol. Macromol. 2020, 149, 861–876. 31987954 10.1016/j.ijbiomac.2020.01.240

[39] H. Furukawa , K. E. Cordova , M. O'Keeffe , O. M. Yaghi , Science 2013, 341, 1230444. 23990564 10.1126/science.1230444

[40] W. Liang , F. Carraro , M. B. Solomon , S. G. Bell , H. Amenitsch , C. J. Sumby , N. G. White , P. Falcaro , C. J. Doonan , J. Am. Chem. Soc. 2019, 141, 14298–14305. 31426638 10.1021/jacs.9b06589

[41] G. Chen , S. Huang , Y. Shen , X. Kou , X. Ma , S. Huang , Q. Tong , K. Ma , W. Chen , P. Wang , J. Shen , F. Zhu , G. Ouyang , Chem 2021, 7, 2722–2742.

[42] J. Tang , J. Liu , Q. Zheng , W. Li , J. Sheng , L. Mao , M. Wang , Angew. Chem. Int. Ed. 2021, 60, 22315–22321; 10.1002/anie.202105634 34382314

[43] W. A. Border , J. W. Harry , E. S. Kamil , A. H. Cohen , J. Clin. Invest. 1982, 69, 451–461. 7056856 10.1172/JCI110469 PMC370995

[44] N. K. Maddigan , A. Tarzia , D. M. Huang , C. J. Sumby , S. G. Bell , P. Falcaro , C. J. Doonan , Chem. Sci. 2018, 9, 4217–4223. 29780551 10.1039/c8sc00825f PMC5942038

[45] O. Boutureira , G. J. L. Bernardes , Chem. Rev. 2015, 115, 2174–2195. 25700113 10.1021/cr500399p

[46] Y. Zhang , K.-Y. Park , K. F. Suazo , M. D. Distefano , Chem. Soc. Rev. 2018, 47, 9106–9136. 30259933 10.1039/c8cs00537k PMC6289631

[47] N. C. Dubey , B. P. Tripathi , ACS Appl. Bio Mater. 2021, 4, 1077–1114. 10.1021/acsabm.0c01293 35014469

[48] M. J. Kummer , Y. S. Lee , M. Yuan , B. Alkotaini , J. Zhao , E. Blumenthal , S. D. Minteer , JACS Au 2021, 1, 1187–1197. 34467357 10.1021/jacsau.1c00180 PMC8397353

[49] X. Chen , J. L. Zaro , W.-C. Shen , Adv. Drug Delivery Rev. 2013, 65, 1357–1369. 10.1016/j.addr.2012.09.039 PMC3726540 23026637

[50] J. M. Bolivar , B. Nidetzky , Langmuir 2012, 28, 10040–10049. 22668007 10.1021/la3012348

[51] L. Pollegioni , G. Molla , Trends Biotechnol. 2011, 29, 276–283. 21397351 10.1016/j.tibtech.2011.01.010

[52] S. Moussa , M. R. V. Horn , A. Shah , L. Pollegioni , C. J. Thibodeaux , E. S. Ruthazer , J. Mauzeroll , J. Electrochem. Soc. 2021, 168, 025502.

[53] E. Rosini , P. D'Antona , L. Pollegioni , Int. J. Mol. Sci. 2020, 21, 4574. 10.3390/ijms21134574 PMC7369756 32605078

[54] K. S. Park , Z. Ni , A. P. Côté , J. Y. Choi , R. Huang , F. J. Uribe-Romo , H. K. Chae , M. O'Keeffe , O. M. Yaghi , Proc. Natl. Acad. Sci. USA 2006, 103, 10186–10191. 16798880 10.1073/pnas.0602439103 PMC1502432

[55] J.-P. Zhang , A.-X. Zhu , R.-B. Lin , X.-L. Qi , X.-M. Chen , Adv. Mater. 2011, 23, 1268–1271. 21381128 10.1002/adma.201004028

[56] M. B. Buergler , A. Dennig , B. Nidetzky , Biotechnol. Bioeng. 2020, 117, 2377–2388. 32369187 10.1002/bit.27372 PMC7384007

[57] C. Zhong , B. Duić , J. M. Bolivar , B. Nidetzky , ChemCatChem 2020, 12, 1350–1358.

[58] M. Pfeiffer , P. Wildberger , B. Nidetzky , J. Mol. Catal. B 2014, 110, 39–46. 10.1016/j.molcatb.2014.09.004 PMC4251788 25484615

[59] E. Jurrus , D. Engel , K. Star , K. Monson , J. Brandi , L. E. Felberg , D. H. Brookes , L. Wilson , J. Chen , K. Liles , M. Chun , P. Li , D. W. Gohara , T. Dolinsky , R. Konecny , D. R. Koes , J. E. Nielsen , T. Head-Gordon , W. Geng , R. Krasny , G.-W. Wei , M. J. Holst , J. A. McCammon , N. A. Baker , Protein Sci. 2018, 27, 112–128. 28836357 10.1002/pro.3280 PMC5734301

[60] I. Dib , D. Stanzer , B. Nidetzky , Appl. Environ. Microbiol. 2007, 73, 331–333. 17056691 10.1128/AEM.01569-06 PMC1797113

[61] I. Dib , B. Nidetzky , BMC Biotechnol. 2008, 8, 72. 18798979 10.1186/1472-6750-8-72 PMC2557008

[62] J. Wiesbauer , J. M. Bolivar , M. Mueller , M. Schiller , B. Nidetzky , ChemCatChem 2011, 3, 1299–1303.

[63] J. M. Bolivar , B. Nidetzky , Biotechnol. Bioeng. 2012, 109, 1490–1498. 22249953 10.1002/bit.24423

[64] J. M. Bolivar , V. Gascon , C. Marquez-Alvarez , R. M. Blanco , B. Nidetzky , Langmuir 2017, 33, 5065–5076. 28464607 10.1021/acs.langmuir.7b00441

[65] D. Valikhani , J. M. Bolivar , A. Dennig , B. Nidetzky , Biotechnol. Bioeng. 2018, 115, 2416–2425. 30036448 10.1002/bit.26802 PMC6836874

[66] M. Hedhammar , S. Hober , J. Chromatogr. A 2007, 1161, 22–28. 17570380 10.1016/j.chroma.2007.05.091

[67] Z. Tang , X. Li , L. Tong , H. Yang , J. Wu , X. Zhang , T. Song , S. Huang , F. Zhu , G. Chen , G. Ouyang , Angew. Chem. Int. Ed. 2021, 60, 23608–23613; 10.1002/anie.202110351 34459532

[68] W. Liang , H. Xu , F. Carraro , N. K. Maddigan , Q. Li , S. G. Bell , D. M. Huang , A. Tarzia , M. B. Solomon , H. Amenitsch , L. Vaccari , C. J. Sumby , P. Falcaro , C. J. Doonan , J. Am. Chem. Soc. 2019, 141, 2348–2355. 30636404 10.1021/jacs.8b10302

[69] R. Chen , J. Yao , Q. Gu , S. Smeets , C. Baerlocher , H. Gu , D. Zhu , W. Morris , O. M. Yaghi , H. Wang , Chem. Commun. 2013, 49, 9500–9502. 10.1039/c3cc44342f 24018656

[70] T. Stassin , I. Stassen , J. Marreiros , A. J. Cruz , R. Verbeke , M. Tu , H. Reinsch , M. Dickmann , W. Egger , I. F. J. Vankelecom , D. E. De Vos , R. Ameloot , Chem. Mater. 2020, 32, 1784–1793.

[71] F.-X. Coudert , ChemPhysChem 2017, 18, 2732–2738. 28657200 10.1002/cphc.201700463

[72] J. Bandekar , Biochim. Biophys. Acta Protein Struct. Mol. Enzymol. 1992, 1120, 123–143. 10.1016/0167-4838(92)90261-b 1373323

[73] F. Mallamace , C. Corsaro , D. Mallamace , S. Vasi , C. Vasi , G. Dugo , Comput. Struct. Biotechnol. J. 2015, 13, 33–37. 25750698 10.1016/j.csbj.2014.11.007 PMC4348435

[74] D. Mallamace , E. Fazio , F. Mallamace , C. Corsaro , Int. J. Mol. Sci. 2018, 19, 3825. 10.3390/ijms19123825 PMC6321052 30513664

[75] J. Kong , S. Yu , Acta Biochim. Biophys. Sin. 2007, 39, 549–559. 17687489 10.1111/j.1745-7270.2007.00320.x

[76] D. M. Byler , H. Susi , Fourier and Computerized Infrared Spectroscopy, International Society for Optics and Photonics, Bellingham, 1985, pp. 289–290.

[77] H. Yang , S. Yang , J. Kong , A. Dong , S. Yu , Nat. Protoc. 2015, 10, 382–396. 25654756 10.1038/nprot.2015.024

[78] C. Hu , Y. Bai , M. Hou , Y. Wang , L. Wang , X. Cao , C.-W. Chan , H. Sun , W. Li , J. Ge , K. Ren , Sci. Adv. 2020, 6, eaax5785. 32064336 10.1126/sciadv.aax5785 PMC6989138

[79] C. L. Cooney , Science 1983, 219, 728–733. 17814034 10.1126/science.219.4585.728

[80] M. Andlar , I. Rezić , D. Oros , D. Kracher , R. Ludwig , T. Rezić , B. Šantek , J. Chem. Technol. Biotechnol. 2017, 92, 623–632.

[81] E. Romero , J. R. Gómez Castellanos , G. Gadda , M. W. Fraaije , A. Mattevi , Chem. Rev. 2018, 118, 1742–1769. 29323892 10.1021/acs.chemrev.7b00650

[82] J. Dong , E. Fernández-Fueyo , F. Hollmann , C. E. Paul , M. Pesic , S. Schmidt , Y. Wang , S. Younes , W. Zhang , Angew. Chem. Int. Ed. 2018, 57, 9238–9261; 10.1002/anie.201800343 PMC6099261 29573076

[83] D. Holtmann , M. W. Fraaije , I. W. C. E. Arends , D. J. Opperman , F. Hollmann , Chem. Commun. 2014, 50, 13180–13200. 10.1039/c3cc49747j 24902635

[84] J. M. Bolivar , B. Nidetzky , Molecules 2019, 24, 3460. 10.3390/molecules24193460 PMC6803829 31554193

[85] A. Lorente-Arevalo , M. Ladero , J. M. Bolivar , Curr. Opin. Green Sustain. Chem. 2021, 32, 100544.

[86] J. M. Bolivar , S. Schelch , T. Mayr , B. Nidetzky , ACS Catal. 2015, 5, 5984–5993.

[87] J. M. Bolivar , T. Consolati , T. Mayr , B. Nidetzky , Biotechnol. Bioeng. 2013, 110, 2086–2095. 23436425 10.1002/bit.24873

[88] J. van Roon , R. Beeftink , K. Schroën , H. Tramper , Curr. Opin. Biotechnol. 2002, 13, 398–405. 12323364 10.1016/s0958-1669(02)00327-0

[89] A. I. Benítez-Mateos , B. Nidetzky , J. M. Bolivar , F. López-Gallego , ChemBioChem 2018, 10, 654–665.

[90] Y. Pan , H. Li , J. Farmakes , F. Xiao , B. Chen , S. Ma , Z. Yang , J. Am. Chem. Soc. 2018, 140, 16032–16036. 30418778 10.1021/jacs.8b09257

[91] F. S. Aalbers , M. W. Fraaije , ChemBioChem 2019, 20, 20–28. 30178909 10.1002/cbic.201800394 PMC6563810

[92] K. Yu , C. Liu , B.-G. Kim , D.-Y. Lee , Biotechnol. Adv. 2015, 33, 155–164. 25450191 10.1016/j.biotechadv.2014.11.005

[93] R. C. Rodrigues , C. Ortiz , Á. Berenguer-Murcia , R. Torres , R. Fernández-Lafuente , Chem. Soc. Rev. 2013, 42, 6290–6307. 23059445 10.1039/c2cs35231a

[94] P. J. Halling , Enzyme Microb. Technol. 1994, 16, 178–206. 7764598 10.1016/0141-0229(94)90043-4

[95] A. Sadana , Chem. Rev. 1992, 92, 1799–1818.

[96] P. Pernot , J.-P. Mothet , O. Schuvailo , A. Soldatkin , L. Pollegioni , M. Pilone , M.-T. Adeline , R. Cespuglio , S. Marinesco , Anal. Chem. 2008, 80, 1589–1597. 18229946 10.1021/ac702230w

[97] A. Hashimoto , T. Nishikawa , T. Oka , K. Takahashi , T. Hayashi , J. Chromatogr. B 1992, 582, 41–48. 10.1016/0378-4347(92)80300-f 1491056

[98] L. Pollegioni , G. Molla , S. Sacchi , E. Rosini , R. Verga , M. S. Pilone , Appl. Microbiol. Biotechnol. 2008, 78, 1–16. 18084756 10.1007/s00253-007-1282-4

[99] V. B. Urlacher , M. Girhard , Trends Biotechnol. 2019, 37, 882–897. 30739814 10.1016/j.tibtech.2019.01.001

