# Supplementary material for: Combining a Genetically Engineered Oxidase with Hydrogen‐Bonded Organic Frameworks (HOFs) for Highly Efficient Biocomposites
Source: Angew Chem Int Ed Engl. 2022 Feb 24;61(16):e202117345. doi: 10.1002/anie.202117345 (PMC9305891; doi:10.1002/anie.202117345)
Supplement: Supplementary file 1 — Supporting Information [file ANIE-61-0-s001.pdf]

Angewandte Chemie
Eine Zeitschrift der Gesellschaft Deutscher Chemiker

Supporting Information

Combining a Genetically Engineered Oxidase with Hydrogen-Bonded Organic Frameworks (HOFs) for Highly Efficient Biocomposites

P. Wied, F. Carraro, J. M. Bolivar, C. J. Doonan, P. Falcaro*, B. Nidetzky**

Supporting Information
©Wiley-VCH 2021
69451 Weinheim, Germany

Combining Genetically Engineered Oxidase with Hydrogen Bonded Organic Framework (HOF) for Highly Efficient Biocomposites

Peter Wied, Francesco Carraro, Juan M. Bolivar, Christian J. Doonan,* Paolo Falcaro* and Bernd Nidetzky*

Abstract: Enzymes incorporated into hydrogen bonded organic frameworks (HOFs) via bottom-up synthesis are promising biocomposites for applications in catalysis and sensing. Here, we explored synthetic incorporation of D-amino acid oxidase (DAAO) with the metal-free tetraamidine/tetracarboxylate-based BioHOF-1 in water. N-terminal enzyme fusion with the positively charged module Z_{basic2} strongly boosted the loading (2.5-fold; ~500 mg enzyme $g_{material}^{-1}$) and the specific activity (6.5-fold; 23 U mg^{-1}). The DAAO@BioHOF-1 composites showed superior activity with respect to every reported carrier for the same enzyme and excellent stability during catalyst recycling. Further, extension to other enzymes, including cytochrome P450 BM3 (used in the production high-value oxyfunctionalized compounds), points to the versatility of genetic engineering as a strategy for the preparation of biohybrid systems with unprecedented properties.

DOI: 10.1002/anie.2021XXXXX

Table of Contents

Table of Contents 2
Experimental Section 3
___I1. Materials. 3
___I2. Enzyme expression and purification. 3
___I3. Enzyme immobilization in BioHOF-1. 4
___I4. Immobilization of Z-DAAO@ZIF-8. 4
___I5. Immobilization of Z-DAAO@MAF-7. 4
___I6. Fourier transform infrared spectroscopy (FTIR). 4
___I7. Optical microscopy. 5
___I8. Confocal laser scanning microscopy (CLSM). 5
___I9. Scanning electron microscopy (SEM). 5
___I10. Powder X-ray Diffraction (PXRD). 5
___I11. ζ-potential. 5
___I12. Determination of protein concentration. 5
___I13. Enzymatic activity assays. 5
___I14. Protease stability. 6
___I15. Recycling of Z-DAAO@MAF-7 and Z-DAAO@BioHOF-1. 7
___I16. Characterization of immobilization performance. 7
___I17. Data analysis. 7
Supporting Results 8
Phase optimization of Z-DAAO@ZIF-8 and Z-DAAO@MAF-7. 8
References 39

Experimental Section

I1. Materials.

Unless stated, chemicals and reagents were from Sigma-Aldrich (Vienna, Austria) or Carl Roth (Karlsruhe, Germany). Ultrapure water was obtained using a TKA GenPure system (JWT GmbH, Jena, Germany). The enzyme constructs used were the following: D-amino acid oxidase (DAAO, EC 1.4.3.3) from *Trigonopsis variabilis* with Strep-tag II[1] (native DAAO) or Z_{basic2}[2] (Z-DAAO) at the N-terminus; cytochrome P450 BM3 (BM3, EC 1.6.2.4) from *Bacillus megaterium* with His-tag (H-BM3) or Z_{basic2} (Z-BM3) at the N-terminus; sucrose phosphorylase from *Bifidobacterium longum* (BlSP) with His-tag (H-BlSP) or Z_{basic2} (Z-BlSP) at the N-terminus; and phosphatase HAD4 from *Escherichia coli* K12 with Z_{basic2} (Z-HAD4) at the N-terminus. Columns for purification (StrepTrap™ HP, HisTrap™ FF, HiTrap™ SP FF) were from Cytiva (Vienna, Austria).

I2. Enzyme expression and purification.

Expression of native DAAO and Z-DAAO. Native DAAO and Z-DAAO were expressed as reported previously[3] with some modifications. Briefly, the relevant *E. coli* colonies were grown in TB-medium (5 g/L glycerol, 24 g/L yeast extract, 12 g/L peptone from casein, 2.31 g/L KH_2PO_4, 12.54 g/L K_2HPO_4) supplemented with 10 mM D-methionine and 50 µg/mL kanamycin (200 mL in 1 L baffled shake flasks). Cell cultures were grown to OD600 of 0.7–1.0 at 37°C and 110 rpm in a CERTOMAT BS-1 incubation shaker (Sartorius, Göttingen, Germany). OD600 was measured spectrophotometrically (DU® 800 UV/Vis Spectrophotometer, Beckman Coulter, Brea, CA, USA). The cultures were induced with β-D-1-thiogalactopyranoside (IPTG, 0.5 mM) and induction was carried out overnight at 25°C. The cells were harvested by centrifugation (20 min, 4 °C, 4.4 krcf, Ultracentrifuge Sorvall RC-5B Superspeed) and washed once with 0.9% NaCl. Cell pellets were stored at -20°C (~5 g wet weight alliquots).

Expression of Z/H-BM3. Z/H-BM3 were expressed as reported previously[4] with some modifications. Briefly, the relevant *E. coli* colonies were grown in TB-medium supplemented with 0.5 mM 5-aminolevulinic acid and 50 µg/mL kanamycin (200 mL in 1 L baffled shake flasks). Z-BM3 cultures were grown to OD600 of 1.8–2.0 at 37°C and 110 rpm. The cultures were induced with 0.5 mM IPTG and induction was carried out overnight at 30°C. H-BM3 cultures were grown to OD600 of 0.8–1.0 at 37°C and 110 rpm. The cultures were induced with 0.5 mM IPTG and induction was carried out overnight at 18°C. The cells were harvested, washed, and stored as described above.

Expression of Z/H-BlSP. Z/H-BlSP were expressed as reported previously[5,6] with some modifications. Briefly, the relevant *E. coli* colonies were grown in TB-medium supplemented with 50 µg/mL kanamycin for Z-BlSP or 100 µg/mL for H-BlSP (200 mL in 1 L baffled shake flasks). Cultures were grown to OD600 of 0.7–1.0 at 37°C and 110 rpm. The cultures were induced with 0.25 mM IPTG and induction was carried out overnight at 25°C. The cells were harvested, washed, and stored as described above.

Expression of ZHAD4. Z-HAD4 was expressed as reported previously[7] with some modifications. Briefly, the relevant *E. coli* colonies were grown in TB-medium supplemented with 50 µg/mL kanamycin (200 mL in 1 L baffled shake flasks). Cultures were grown to OD600 of 0.7–1.0 at 37°C and 110 rpm. The cultures were induced with 0.5 mM IPTG and induction was carried out overnight at 18°C. The cells were harvested, washed, and stored as described above.

Cell disruption. Prior to cell disruption the cells were resuspended in a 4-fold volume of the corresponding binding buffer used for purification. Cell disruption was performed by ultra-sonification with a Sonic Dismembrator Model 505 (Fisher Scientific, Vienna, Austria) using the following protocol: 6 min in total, alternating 2 s pulse on/4 s pulse off at 60% amplitude. The cell extract was recovered (20.000 rcf, 4°C, 60 min) afterwards and the clear supernatant was sterile filtered (0.2 µm syringe filter).

Purification of Z-DAAO, Z-BM3, Z-BlSP and Z-HAD4: Z-DAAO, Z-BM3, Z-BlSP and Z-HAD4 were purified with a HiTrap™ SP FF 5 mL column on a ÄKTA-system (ÄKTAprime plus or ÄKTA go, Cytiva, Vienna, Austria) with UV detection (Figure S1-S4). Purification was at 4°C. Buffer A (50 mM HEPES, pH 7.5, 250 mM NaCl) was used for the loading of the cell free extract. Buffer B (50 mM HEPES, pH 7.5, 2 M NaCl) was used for isocratic (Z-DAAO) or gradient elution (Z-BM3, Z-BlSP, Z-HAD4). The flow rate was 3 ml min^{-1}. Typically, 20-30 mL cell-free extract (from ~5 g cell pellet) was loaded per purification. Gradient elution was performed for 20 column volumes (100 mL). The purified enzyme was pooled and the buffer was exchanged by ultrafiltration (Vivaspin® Ultrafiltration Unit, Sartorius, Göttingen, Germany) to Buffer A. Purified enzymes were concentrated to a final concentration of 5-25 mg/mL and directly used for immobilization experiments or stored at -20°C.

Purification of native DAAO (Strep II[1] tagged): Native DAAO was purified with a StrepTrap™ HP 5 mL column on a ÄKTA-system (ÄKTAprime plus or ÄKTA go, Cytiva, Vienna, Austria) with UV detection (Figure S1). Purification was at 4°C. Buffer A (100 mM Tris-HCl, pH 8, 150 mM NaCl, 1mM EDTA) was used for the loading of the cell free extract. Buffer B (100 mM Tris-HCl, pH 8, 150 mM NaCl, 1mM EDTA, 2.5 mM desthiobiotin) was used for isocratic elution. The flow rate was 3 ml min^{-1}. Typically, 20-30 mL cell-free extract (from ~5 g cell pellet) was loaded per purification The purified enzyme was pooled and the buffer was exchanged by

ultrafiltration (Vivaspin® Ultrafiltration Unit, Sartorius, Göttingen, Germany) to HEPES buffer (50 mM, pH 7.5). Purified enzymes were concentrated to a final concentration of 5-25 mg/mL and directly used for immobilization experiments or stored at -20°C.

Purification of H-BM3, H-BlSP: H-BM3 and H-BlSP were purified with a HisTrap™ FF 5 mL column on a ÄKTA-system (ÄKTAprime plus or ÄKTA go, Cytiva, Vienna, Austria) with UV detection (Figure S2-S3). Purification was at 4°C. Buffer A (50 mM HEPES, pH 7.5, 500mM NaCl, 20 mM imidazole) was used for the loading of the cell free extract. Buffer B (50 mM HEPES, pH 7.5, 500mM NaCl, 500 mM imidazole) was used for gradient elution. The flow rate was 3 ml min^{-1}. Typically, 20-30 mL cell-free extract (from ~5 g cell pellet) was loaded per purification Gradient elution was performed for 20 column volumes (100 mL). The purified enzyme was pooled and the buffer was exchanged by ultrafiltration (Vivaspin® Ultrafiltration Unit, Sartorius, Göttingen, Germany) to HEPES buffer (50 mM, pH 7.5). Purified enzymes were concentrated to a final concentration of 5-25 mg/mL and directly used for immobilization experiments or stored at -20°C. For H-HAD4 the purification did not achieve the desired amount of active enzyme, therefore it was not further examined in immobilization studies.

SDS-PAGE. Purified enzymes were additionally checked by SDS PAGE. Ten microlitre of the sample was loaded on a NuPAGE 4–12% Bis–Tris Protein Gel (Thermo Fisher Scientific Inc., Waltham, MA, USA). The protein separation was performed at 200 V for 60 min in 1× MOPS buffer (NuPAGE MOPS SDS Running Buffer 20×, Invitrogen). The finished gel was stained with staining solution (75:500:425; acetic acid:ethanol:water; v:v:v; 2.5 g/L of Brilliant blue R250) for ~ 30 min and destained (75:200:725; acetic acid:ethanol:water, v:v:v) to visualize the protein bands. Purified enzymes were concentrated to a final concentration of 5-25 mg/mL and directly used for immobilization experiments or stored at -20°C.

I3. Enzyme immobilization in BioHOF-1.

Procedure for enzyme immobilization in BioHOF-1 was adopted from literature.[8] Purified enzymes in HEPES buffer (50 mM, pH 7.5) were used for immobilization, where Z-DAAO/BM3/BlSP/HAD4 were supplemented with 250 mM NaCl. 1 mL of an aqueous solution containing 4 mM (methanetetrayltetrakis(benzene-4,1-diyl))tetrakis(aminomethaniminium) and 2 mg mL^{-1} enzyme, then left to stir for 10 min at 22°C. Afterwards, 1 mL of 3 mM 4,4',4'',4'''-methanetetrayltetrabenzoate in H_2O containing 1% NH_3(aq) was added dropwise and left to stir at 22°C for 1 h. Afterwards, the samples were centrifuged (20000 rcf for 2 min) and washed once with 0.5 % Tween 20 and three times with HEPES (50 mM, pH 7.5) to remove loosely bound enzyme. For comparison, BioHOF-1 was prepared as described above, but without addition of enzyme.

I4. Immobilization of Z-DAAO@ZIF-8.

Z-DAAO@ZIF-8 was prepared similarly as described by Liang et al.[9] but with adjusted precursor concentrations to obtain *sod* topology (See Results and Discussion: Phase optimization of Z-DAAO@ZIF-8 and Z-DAAO@MAF-7.). 1 mL of 80 mM $Zn(OAc)_2 \cdot 2H_2O$ in H_2O was added to 1 mL of an aqueous solution containing 1 mg mL^{-1} Z-DAAO and 1280 mM 2-methyl-1*H*-imidazole, stirred at 22°C for 1 h. Afterwards, the samples were centrifuged (20000 rcf for 2 min) and washed once with 0.5 % Tween 20 and three times with 20 mM HEPES (pH 8) to remove loosely bound enzyme. For comparison, ZIF-8 was prepared as described above, but without addition of enzyme.

I5. Immobilization of Z-DAAO@MAF-7.

Z-DAAO@MAF-7 was prepared as described previously by Liang et al.[9] but with increased NH_3(aq) to obtain *sod* topology (See Results and Discussion: Phase optimization of Z-DAAO@ZIF-8 and Z-DAAO@MAF-7.). 1 mL of 80 mM $Zn(NO_3)_2 \cdot 6H_2O$ in H_2O was added to 1 mL of an aqueous solution containing 2 mg mL^{-1} Z-DAAO, 240 mM 3-methyl-1*H*-1,2,4-triazole and 1% NH_3(aq) , stirred at 22°C for 1 h. Afterwards, the samples were centrifuged (20000 rcf for 2 min) and washed once with 0.5 % Tween 20 and three times with 20 mM HEPES (pH 8) to remove loosely bound enzyme. For comparison, MAF-7 was prepared as described above, but without addition of enzyme.

I6. Fourier transform infrared spectroscopy (FTIR).

FT-IR spectra were recorded on a Bruker ALPHA FTIR spectrometer (Bruker corporation, Billerica, MA, USA) using the ATR accessory with a diamond window in the range of $\tilde{\nu}$ 400 – 4000 cm^{-1}, 256 scans, resolution 2 cm^{-1}. Samples were dropcast on the diamond window and dried using a nitrogen stream, prior to the measurement.

I7. Optical microscopy.

Optical microscope images were acquired with a Zeiss Axio Scope A1 optical microscope (Carl Zeiss, Jena, Germany).

I8. Confocal laser scanning microscopy (CLSM).

Z-DAAO was tagged with Invitrogen Alexa Fluor™ 647 Protein Labelling Kit (Thermo Fisher Scientific, USA) according to the user guide for detection of the immobilized enzyme by CLSM.

Sample preparation. Briefly, the NHS ester (or succinimidyl ester) of Alexa Fluor® 647 was used to label primary amines ($R\text{-}NH_2$) of Z-DAAO. 50 µL of sodium bicarbonate (1 M in ddH_2O) was added to 500 µL of Z-DAAO (2 mg/mL). The reaction solution was transferred to a vial containing the reactive dye and a magnetic stirrer at incubated at 22°C for 1h. To remove unreacted dye a Zeba™ Dye and Biotin Removal Column (Thermo Fisher Scientific, USA) contained in the labelling kit was used. The column was washed with bicarbonate buffer (0.2 M, pH 9.4) three times, by centrifugation at 1000 rcf for 2 min, prior to applying the reaction solution, followed by centrifugation for 1000 rcf for 2 min. The flowthrough was collected and used for immobilization. After immobilization, 10 µL sample was dropped on a microscope slide, with a cover glass and sealed using clear nail-polish.

Image acquisition and processing. CLSM was performed by excitation with a 635 nm laser, using a Leica TCS SPE (Leica microsystems, Germany). The laser intensity was set to 100% and the gain set to 1200. The acquired images were cropped and recolored using Affinity Designer 1.10.4.1198 (Serif (Europe) Ltd., Nottingham, UK).

I9. Scanning electron microscopy (SEM).

SEM micrographs were collected using Tescan VEGA 3 SEM (TESCAN ORSAY HOLDING, a.s., Brno, Czech Republik) with tungsten source filament (20 kV). Prior the analysis the powder samples were dropcasted on a piece of Si (100) and sputter-coated with gold. SEM Image analysis was performed with FIJI.[10]

I10. Powder X-ray Diffraction (PXRD).

XRD patterns were acquired using a Rigaku® SmartLab II (Rigaku Europe SE, Neu-Isenburg, Germany) equipped with a Cu anode (9 kW, λ=1.5406Å). Prior the analysis the powder samples were dropcasted on a piece of Si (100) and dried overnight at 22°C.

I11. ζ-potential.

ζ-potential measurements via electrophoretic light scattering were performed using the litesizer 500 (Anton Paar® GmbH, Graz, Austria). Sample preparation was performed by dissolving each sample in filtered (0.2µm filter) ddH_2O to reach a final Z-DAAO concentration of ~0.1 mg/mL for free and immobilized Z-DAAO. Material without Z-DAAO was diluted in an equivalent amount (1:10) after synthesis. Capillary of the zeta potential cuvette was filled with ~100 µL sample, capped and measured.

I12. Determination of protein concentration.

The protein concentration before and after immobilization was determined according to the Bradford assay[11] (ROTI®Quant, Carl Roth, Karlsruhe, Germany) or the bicinchoninic acid assay[12] (Pierce™ BCA Protein Assay Kit, Thermo Fisher Scientific, Rockford, USA) using Albumin Fraction V (bovine serum) as a standard for calibration (**Figure S5-S6**). For enzymes encapsulated in ZIF-8 and MAF-7 the Bradford assay showed no interference by MOF precursors. For Z-DAAO encapsulated in BioHOF-1, the BCA assay showed no interference by the HOF precursors. UV-VIS spectroscopy was performed on a FLUOstar® Omega multi-mode microplate reader (BMG Labtech, Ortenberg, Germany).

I13. Enzymatic activity assays.

DAAO: In a standard experiment the enzymatic activity of DAAO and immobilized DAAO was determined by monitoring the initial O_2 consumption rate during the conversion of D-methionine (Scheme S1).[13] Typically the linear initial-rate of O_2 consumption after an initial equilibration period (~15s) and within the first 2 min of the reaction was used for linear regression analysis (**Figure S7**). O_2 concentrations were measured with a fiber-optic oxygen meter consisting of a FireStingO2 (FSO2-4) control unit with an OXROB10

probe (PyroScience GmbH, Aachen, Germany). The reaction set-up consisted of an open glass vial containing 4 mL reaction mix in a water bath (30°C) with magnetic stirring (6 x 3 mm, 400 rpm). The reaction mix (4 mL) consisted of D-methionine (20 mM) and DAAO or immobilized DAAO in air-saturated HEPES buffer (20 mM, pH 8). The specific activity of purified DAAO and Z-DAAO varied between 45-75 U mg_{enyzme}^{-1} depending on expression batch and storage time.[14] The conversion of D-serine and cephalosporin c was performed with a final substrate concentration of 10 mM (Scheme S1).

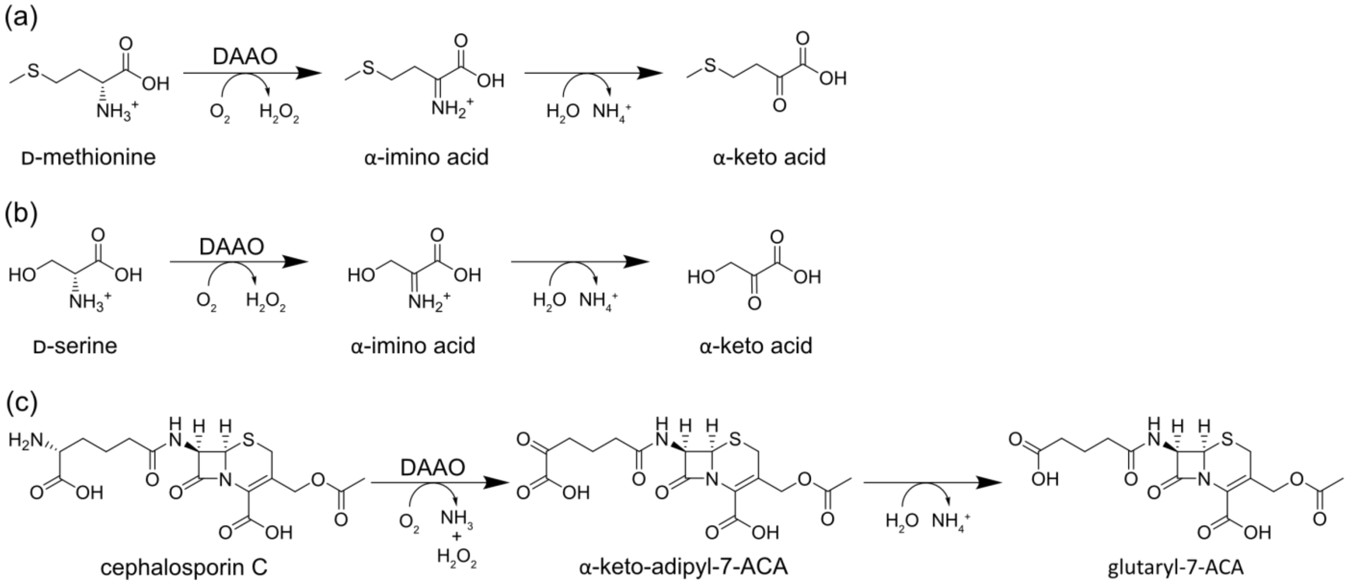

Scheme S1. Scheme of the reactions catalyzed by D-amino acid oxidase. Conversion of (a) d-methionine, (b) d-serine and (c) cephalosporin C

BM3: The activity of BM3 and immobilized BM3 was measured through the initial O_2 consumption rate during the hydroxylation of lauric acid at 30°C.[4] The reaction mix (4 mL) contained lauric acid (2 mM), $NADP^+$ (0.2 mM), glucose (200 mM), glucose dehydrogenase (1 mg/mL) and free or immobilized Z/H-BM3 in air-saturated HEPES buffer (50 mM, pH 7.4).

BlSP: The activity of BlSP and immobilized BlSP was determined by continuously measuring the formation of NADH in a cascade reaction containing BlSP, phosphoglucomutase and glucose-6-phosphate dehydrogenase.[6,15] The reaction was performed in a cuvette for 5 min at 30°C. The reaction mix (2 mL) consisted of phosphoglucomutase (3 U mL^{-1}), glucose-6-phosphate dehydrogenase (3.4 U mL^{-1}), NAD^+ (2.5 mM), sucrose (250 mM) and BlSP or immobilized BlSP in HEPES buffer (50 mM, pH 7.4) The NADH formation was monitored at 340 nm using a Varian Cary® 50 Bio UV-Vis Spectrophotometer (Varian, Inc., Palo Alto, California, USA) fitted with a magnetic stirrer.

HAD4: The activity of HAD4 and immobilized HAD4 was determined discontinuously by measuring the released phosphate during the conversion of α-glucose 1-phosphate.[6,15] The reaction was performed in a thermomixer at 30°C. The reaction mix (1 mL) consisted of $MgCl_2$ (25 mM), NaCl (100 mM), α-glucose 1-phosphate (15 mM) and HAD4 or immobilized HAD4. Every 10 min 100 µL sample was taken and the reaction was stopped by adding 100 µL 1 M NaOH. After centrifugation for 2 min at 15000 rpm the amount of released phosphate was measured according to Saheki et al.[16] with modifications on a FLUOstar® Omega multi-mode microplate reader (BMG Labtech, Ortenberg, Germany). Briefly, Molybdate reagent was prepared containing 15 mM ammonium molybdate and 100 mM Zinc acetate. The working reagent consisted of 4 parts Molybdate reagent and 1 part 10% ascorbic acid reagent (4+1). As a standard a 1 M KH_2PO_4 solution was prepared and diluted appropriately. 10 µL sample was mixed with 150 µL working reagent and incubated for 15 min at 25°C, subsequently, the absorbance was measured at 850 nm.

I14. Protease stability.

Free Z-DAAO and Z-DAAO@BioHOF-1 was incubated in a buffer solution containing 5 mg mL^{-1} trypsin from porcine pancreas at 37°C for 1 h. As a control, Z-DAAO and Z-DAAO@BioHOF-1 was incubated under the same conditions, excluding trypsin.

I15. Recycling of Z-DAAO@MAF-7 and Z-DAAO@BioHOF-1.

Recycling of each material was performed in 2 mL tubes on and end-over-end rotator at a reaction volume between 1 mL and 1.2 mL to ensure sufficient O_2 supply. Each recycling step was performed for 2 min at 30°C. 20 $mg_{wet\ weight}$ mL^{-1} material was employed for each reaction. The reaction medium contained 20 mM HEPES (pH 8) and 10 mM D-methionine. After each cycle, the catalyst was recovered by centrifugation (20000 rcf for 2 min at 4°C) and reused in freshly prepared medium.

I16. Characterization of immobilization performance.

The **protein yield** (Y_P) was calculated as follows:

$$Y_P = \frac{P_{immobilzed}}{P_{contacted}} = \frac{P_{contacted} - P_{residual}}{P_{contacted}}$$

Where $P_{residual}$ is determined by measuring the protein concentration in the supernatant after immobilization.

Protein loading ($P_{loading}$) was calculated as follows:

$$P_{loading} = \frac{m_{protein\ immobilized}}{m_{carrier}}$$

Where $m_{protein\ immobilized}$ is the mass of the immobilized biocatalyst and $m_{carrier}$ the mass of carrier.

The **specific activity of the material** ($Specific\ activity_{material}$) was determined as follows:

$$Specific\ activity_{material} = \frac{A_{immobilized}}{m_{material}}$$

Where $A_{immobilized}$ is the activity of the immobilized biocatalyst and $m_{material}$ the mass of the carrier.

To determine the effectiveness of the immobilization the **effectiveness factor** (η) was calculated:

$$\eta = \frac{Specific\ activity_{immobilized\ enzyme}}{Specific\ activity_{free\ enzyme}}$$

Where $Specific\ activity_{immobilized\ enzyme}$ is the specific activity of the enzyme per mass of enzyme bound to the carrier (U mg_{enzyme}^{-1}).

Apparent Michaelis-Menten kinetic parameters were assessed by measuring the O_2 consumption rate at varying D-methionine concentrations (0.1 – 10 mM). Nonlinear regression analysis was performed using the Michaelis-Menten model:

$$v_0 = \frac{V_{max}[S]}{K_M + [S]}$$

Where v_0 is the initial rate of reaction, V_{max} the maximum rate, K_M the substrate concentration at $\frac{V_{max}}{2}$ and $[S]$ the initial substrate concentration.

$$V_{max} = k_{cat} * [E]$$

Where k_{cat} is the turnover number and $[E]$ the enzyme concentration.

I17. Data analysis.

Data analysis and fitting was performed with Origin(Pro), Version 2021b (OriginLab Corporation, Northampton, MA, USA).

Supporting Results

Phase optimization of Z-DAAO@ZIF-8 and Z-DAAO@MAF-7.

Initially, the immobilization of DAAO@ZIF-8 was explored using an established protocol[9] with a metal to ligand ratio (M/L) of 1:16 mM during the synthesis. To confirm the topology of each material the crystallinity of each biocomposite was monitored using powder x-ray diffraction (PXRD). PXRD patterns revealed that pure ZIF-8 formed with *sod* phase, whereas Z-DAAO@ZIF-8 leads to the formation of ZIF-L (**Figure S10**). ZIF-L is a 2D ZIF with a decreased pore size (vs. ZIF-8).[17] To obtain porous ZIF-8 with *sod* topology, the synthesis conditions were optimized. The enzyme preparation contains 250 mM NaCl to minimize unspecific ionic interactions of Z-DAAO and its purification tag (Z_{basic2}).[2,5] We examined the addition of NaCl (50/100 mM) during the synthesis of ZIF-8, which lead to the formation of ZIF-L (**Figure S12**). By increasing the concentration of 2-methyl-1*H*-imidazole (M/L = 1:32), ZIF-8 and Z-DAAO@ZIF-8 resulted in the formation of *sod*, in the presence of 100 mM NaCl (**Figure S14**). Therefore the M/L of 1:32 was chosen for the one-pot immobilization of Z-DAAO@ZIF-8. Next, the synthesis of Z-DAAO@MAF-7 was examined using PXRD. Initial one-pot immobilization of Z-DAAO@MAF-7 with a previously reported method[9] resulted in the formation of amorphous material (**Figure S11**). In contrast to Z-DAAO@ZIF-8, NaCl did not affect the synthesis of MAF-7 (**Figure S13**). However, to obtain crystalline Z-DAAO@MAF-7 the concentration of base (NH_3(aq)), required in the synthesis of MAF-7, had to be increased (from 0.25% to >0.5% **Figure S15**). Although Z-DAAO influences the crystallinity of ZIF-8 and MAF-7 no such effect could be observed for Z-DAAO@BioHOF-1 (**Figure 3a**). The optimized immobilization protocols can be seen in **I3 – I5**. Interestingly, while Z-DAAO@ZIF-L/ZIF-8 was inactive, the amorphous MAF-7 lead to an increase in immobilization performance over Z-DAAO@MAF-7 with a sodalite topology (**Figure S38**).

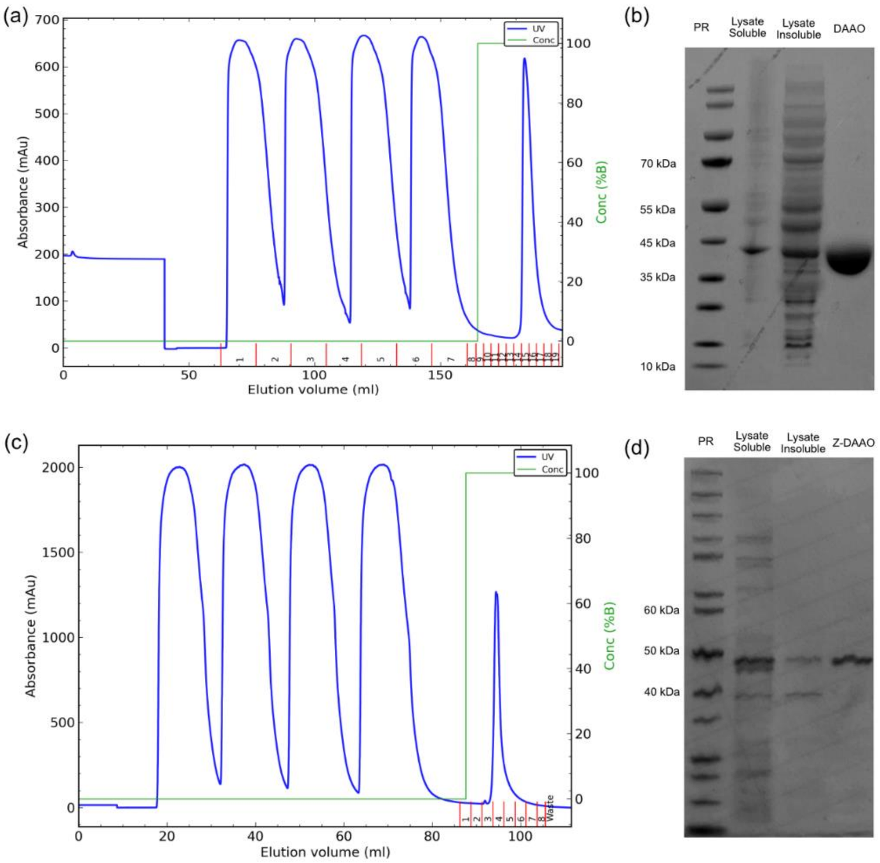

Figure S1. Purification of native DAAO (Strep II[1] tagged) and Z-DAAO. (a) Elution profile of native DAAO using StrepTrap™ HP 5 mL column, where Conc (%B) is the concentration of elution buffer (100 mM Tris-HCl, pH 8, 150 mM NaCl, 1mM EDTA, 2.5 mM desthiobiotin). Flow rate = 3 ml/min (b) SDS polyacrylamide gel of soluble/insoluble lysate fraction and purified native DAAO. (c) Elution profile of Z-DAAO purification using HiTrap™ SP FF 5 mL column, where Conc (%B) is the concentration of elution buffer (50 mM HEPES, pH 7.5, 2 M NaCl). Flow rate = 3 ml/min. (d) SDS polyacrylamide gel of soluble/insoluble lysate fraction and purified Z-DAAO. For detailed procedure see I2 Enzyme expression and Purification.

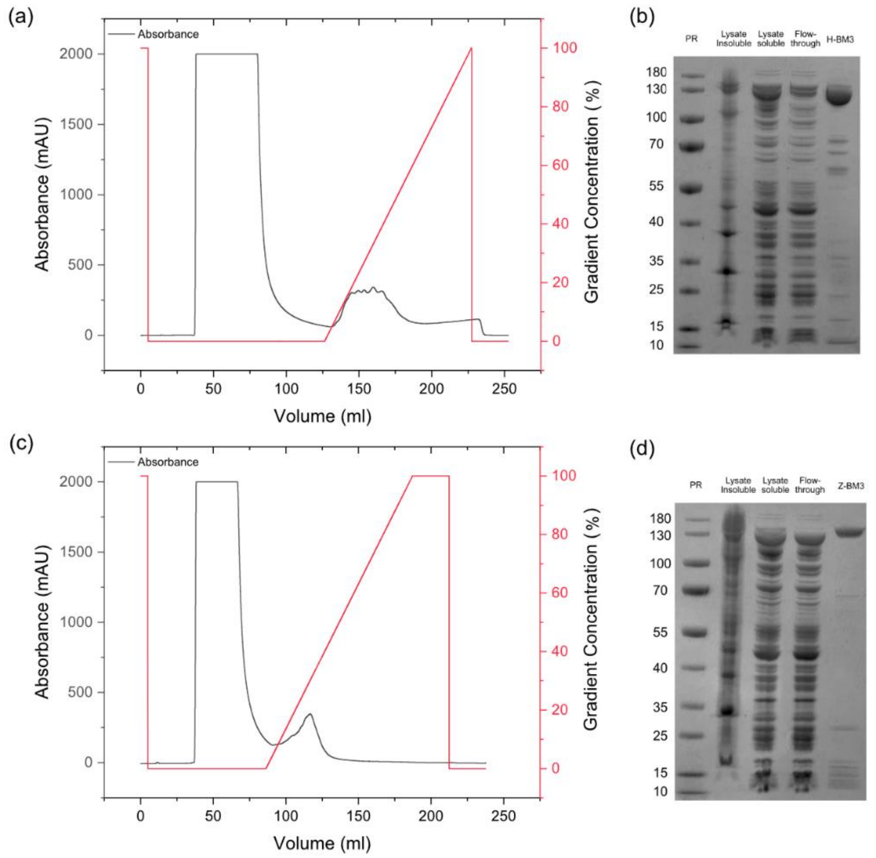

Figure S2. Purification of H-BM3 and Z-BM3. (a) Elution profile of H-BM3 using HisTrap™ FF 5 mL column, where Gradient Concentration (%) is the concentration of elution buffer (50 mM HEPES, pH 7.5, 500mM NaCl, 500 mM imidazole). Flow rate = 3 ml/min (b) SDS polyacrylamide gel of soluble/insoluble lysate fraction, flowthrough and purified H-BM3. (c) Elution profile of Z-BM3 purification using HiTrap™ SP FF 5 mL column, where Gradient Concentration (%) is the concentration of elution buffer (50 mM HEPES, pH 7.5, 2 M NaCl). Flow rate = 3 ml/min. (d) SDS polyacrylamide gel of soluble/insoluble lysate fraction, flowthrough and purified Z-BM3. For detailed procedure see I2 Enzyme expression and Purification.

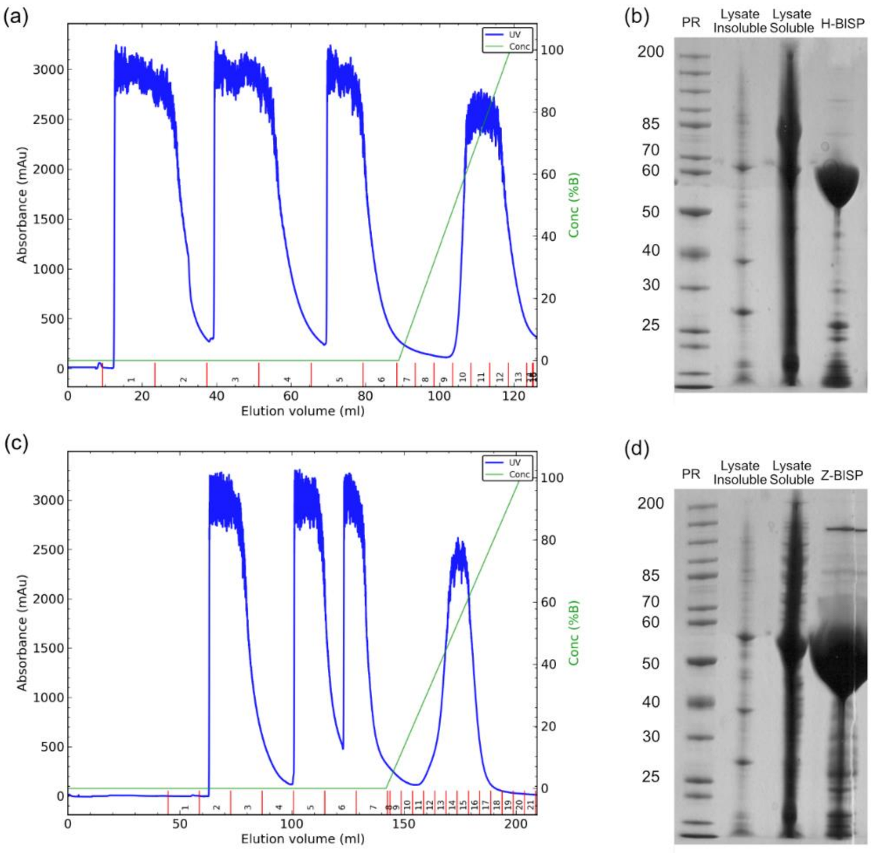

Figure S3. Purification of H-BlSP and Z-BlSP. (a) Elution profile of H-BlSP using HisTrap™ FF 5 mL column, where Conc (%B) is the concentration of elution buffer (50 mM HEPES, pH 7.5, 500mM NaCl, 500 mM imidazole). Flow rate = 3 ml/min (b) SDS polyacrylamide gel of soluble/insoluble lysate fraction, flowthrough and purified H-BlSP. (c) Elution profile of Z-BlSP purification using HiTrap™ SP FF 5 mL column, where Conc (%B) is the concentration of elution buffer (50 mM HEPES, pH 7.5, 2 M NaCl). Flow rate = 3 ml/min. (d) SDS polyacrylamide gel of soluble/insoluble lysate fraction, flowthrough and purified Z-BlSP. For detailed procedure see I2 Enzyme expression and Purification.

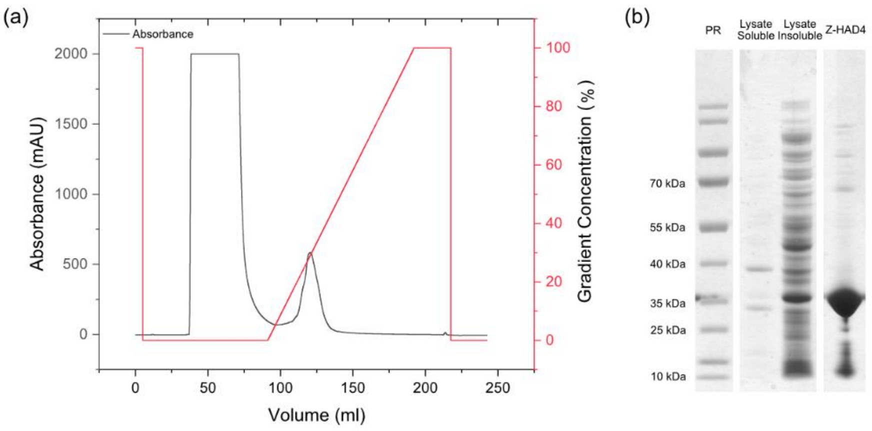

Figure S4. Purification of Z-HAD4. (a) Elution profile of Z-HAD4 purification using HiTrap™ SP FF 5 mL column, where Gradient Concentration (%) is the concentration of elution buffer (50 mM HEPES, pH 7.5, 2 M NaCl). Flow rate = 3 ml/min. (b) SDS polyacrylamide gel of soluble/insoluble lysate fraction, flowthrough and purified Z-HAD4. For detailed procedure see I2 Enzyme expression and Purification.

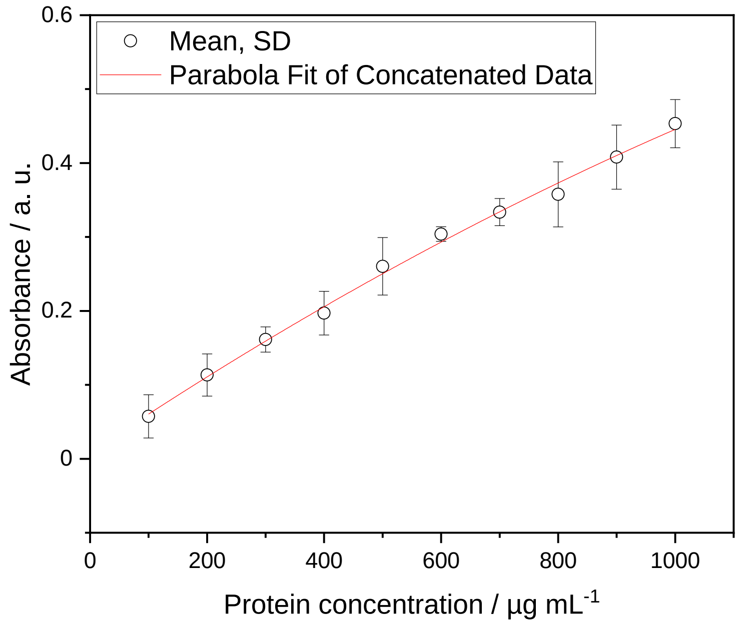

Figure S5. Example calibration of Bradford assay. Albumin Fraction V (bovine serum) was used as a standard for calibration of the assay.

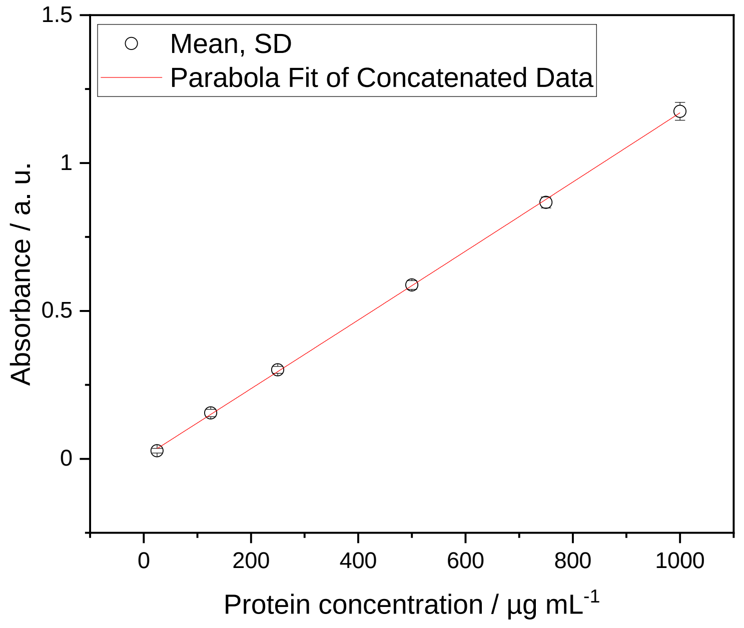

Figure S6. Example calibration of Pierce BCA assay. Albumin Fraction V (bovine serum) was used as a standard for calibration of the assay.

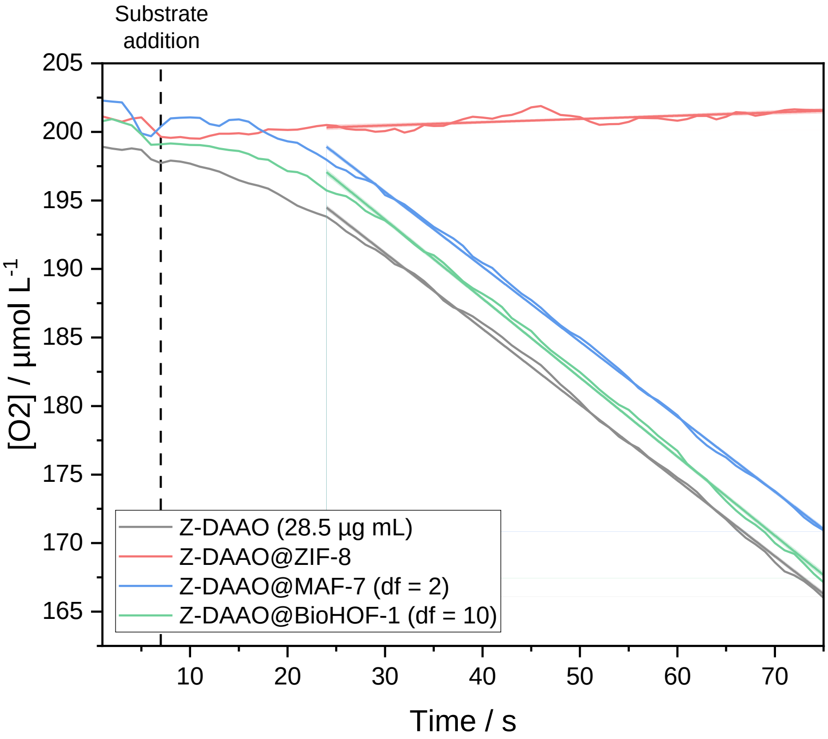

Figure S7. Example Oxygen time courses of free Z-DAAO and immobilized Z-DAAO. Due to the response time of the O_2 sensor linear regression was performed after an initial equilibration period (~15s).

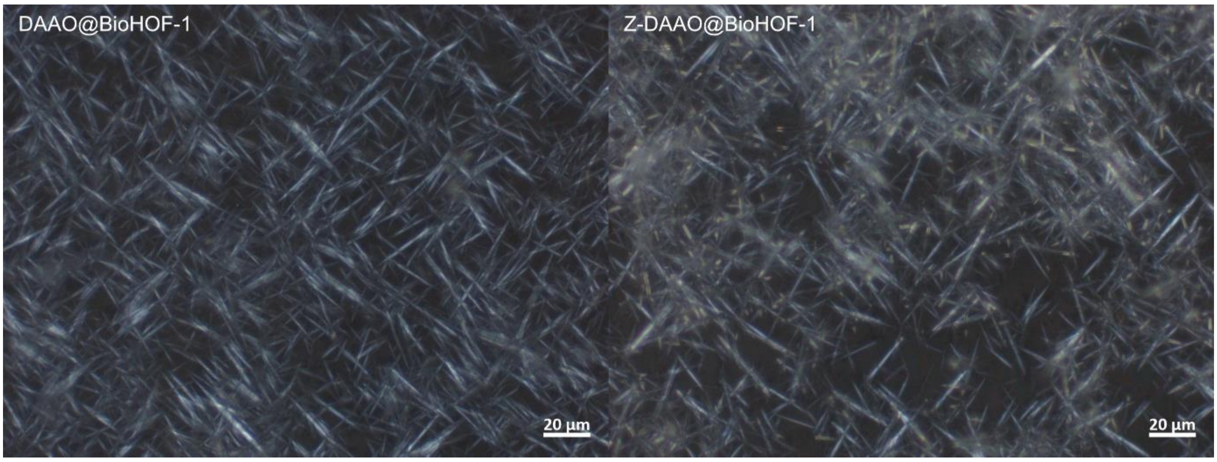

Figure S8. Optical microscopy images of native DAAO@BioHOF-1 and Z-DAAO@BioHOF-1.

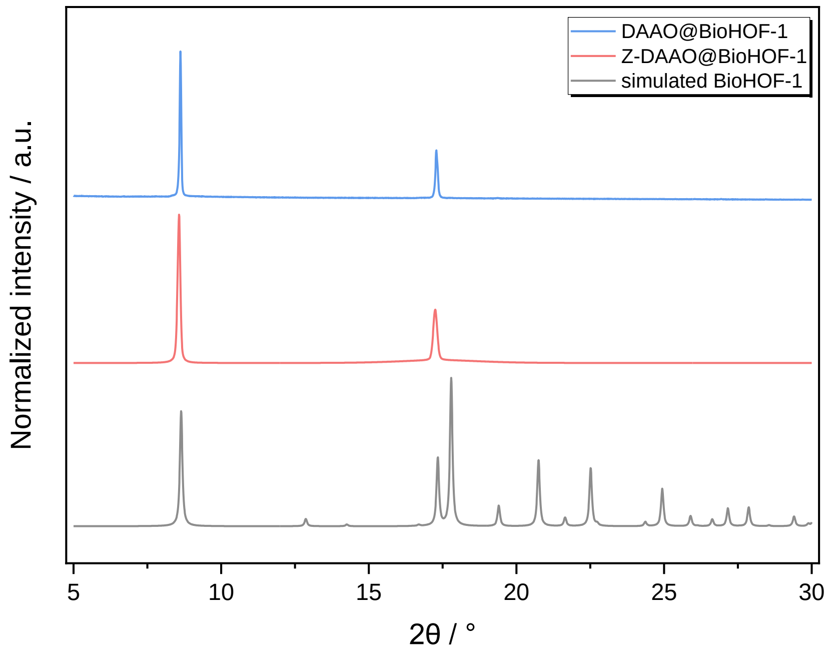

Figure S9. PXRD patterns of native DAAO (DAAO) and Z-DAAO immobilized with BioHOF-1.

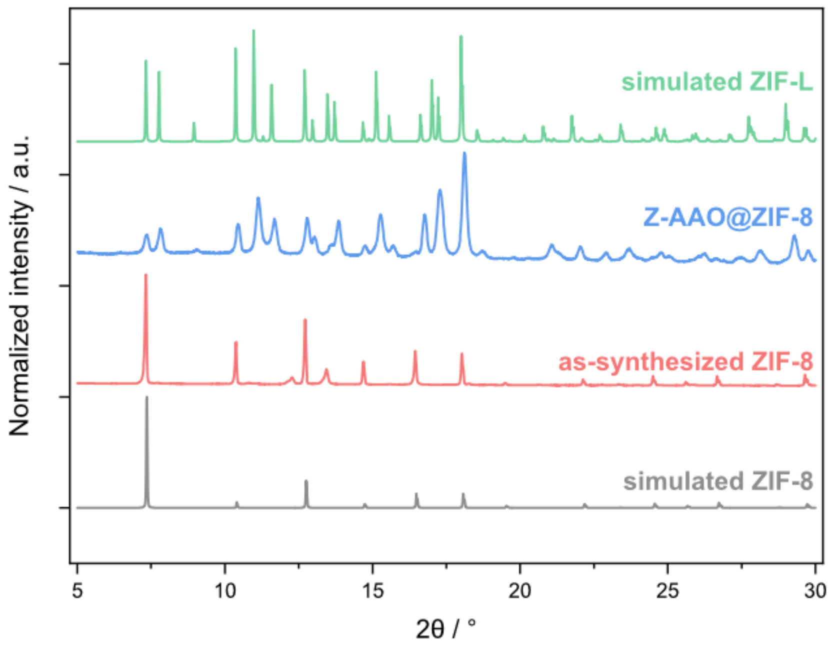

Figure S10. PXRD patterns of ZIF-8 and Z-DAAO@ZIF-8 obtained with 40 mM $Zn(OAc)_2$ and 640 mM 2-methyl-1H-imidazole during the MOF synthesis.

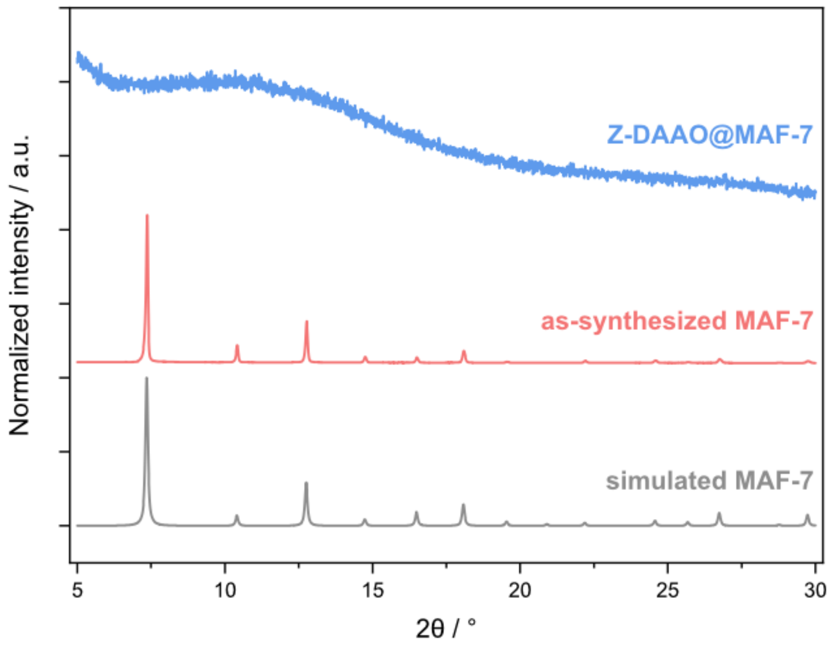

Figure S11. PXRD patterns of MAF-7 and Z-DAAO@MAF-7 obtained with 40 mM $Zn(NO_3)_2$ and 120 mM 3-methyl-1,2,4-triazole and 0.25% NH_3(aq) during the MOF synthesis.

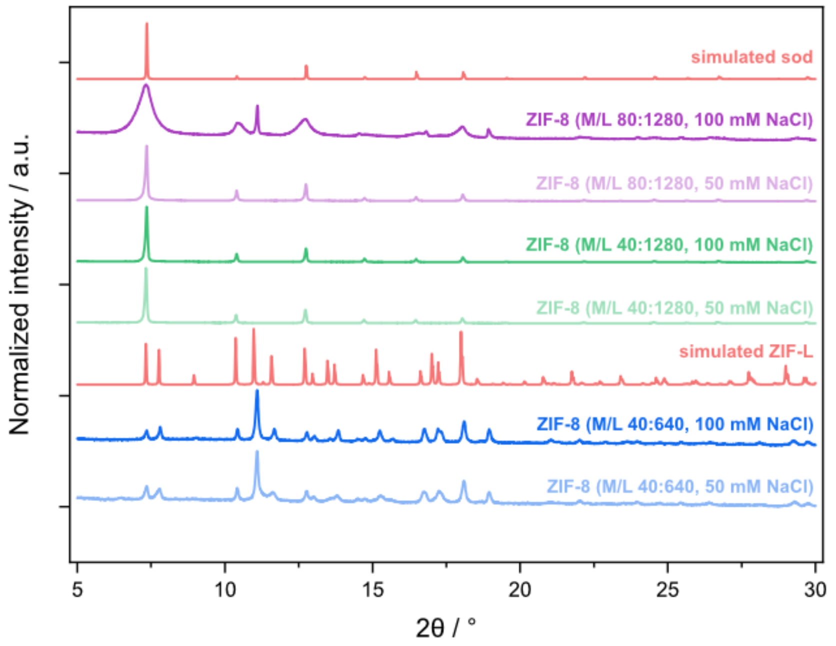

Figure S12. PXRD patterns of ZIF-8 with varied metal/linker concentrations and addition of 50/100 mM NaCl during the MOF synthesis.

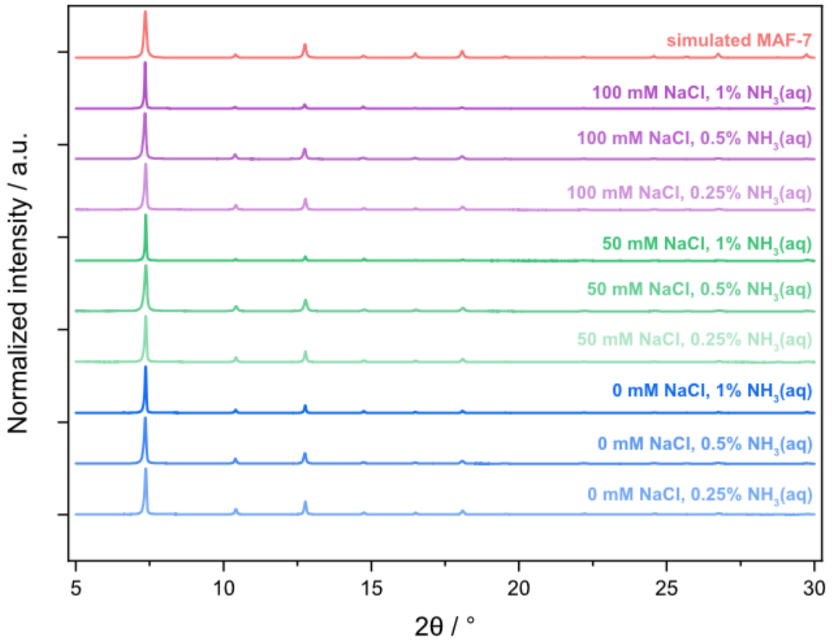

Figure S13. PXRD patterns of MAF-7 with varied NH_3(aq) concentrations and addition of 0/50/100 mM NaCl during the MOF synthesis.

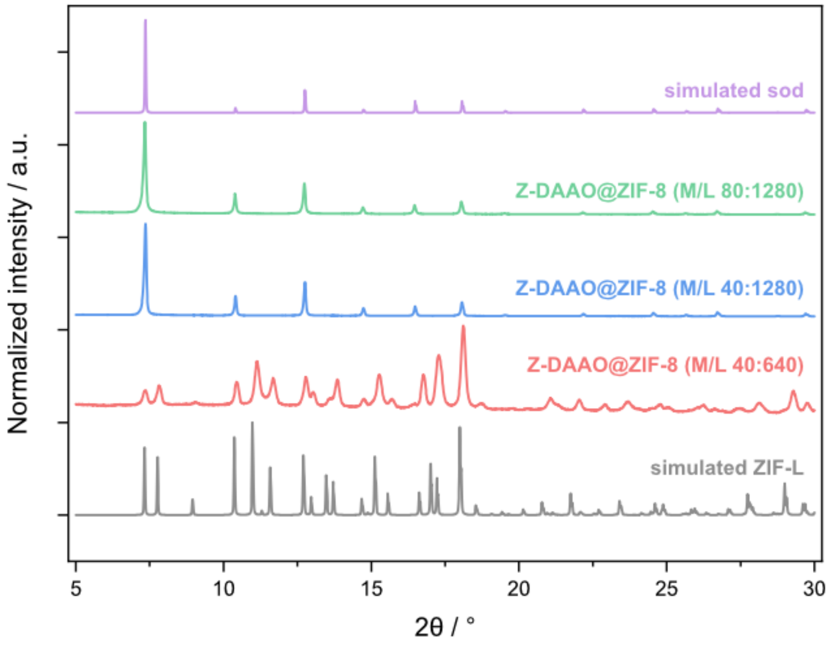

Figure S14. PXRD patterns of Z-DAAO@ZIF-8 with varied metal/ligand (M/L) ratios during MOF synthesis.

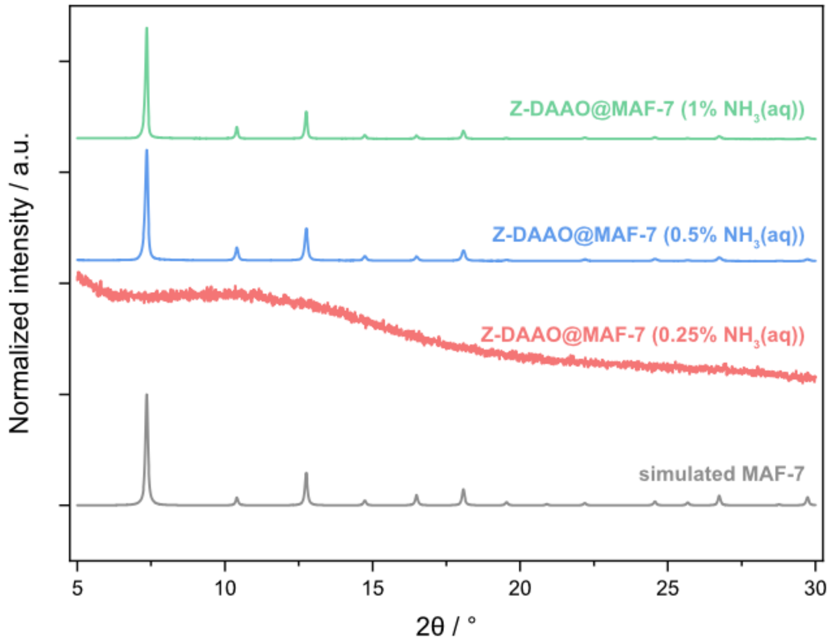

Figure S15. PXRD patterns of Z-DAAO@MAF-7 with varied NH_3(aq) concentrations during MOF synthesis.

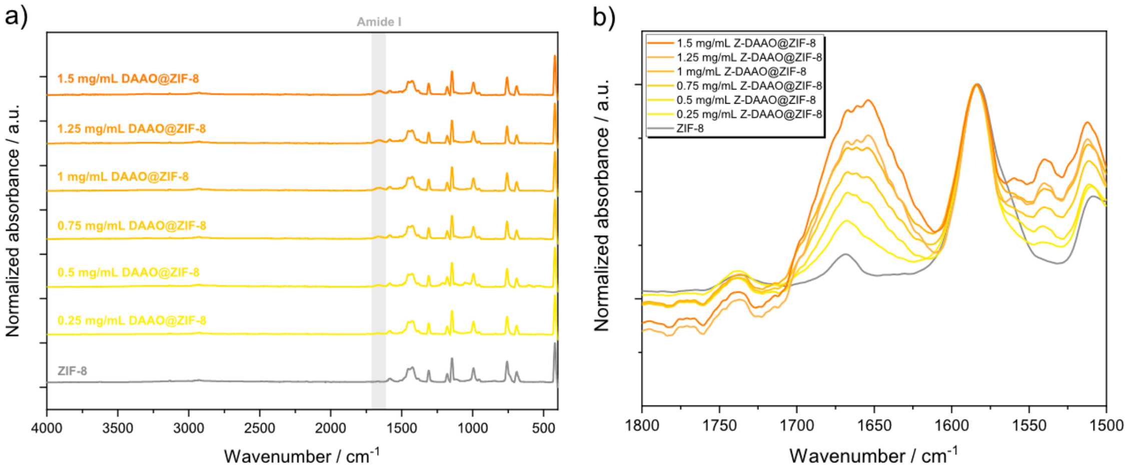

Figure S16. ATR-FTIR spectra of ZIF-8 and Z-DAAO@ZIF-8 with varied Z-DAAO concentration. (a) Spectra overview and (b) Detailed view of amid I region.

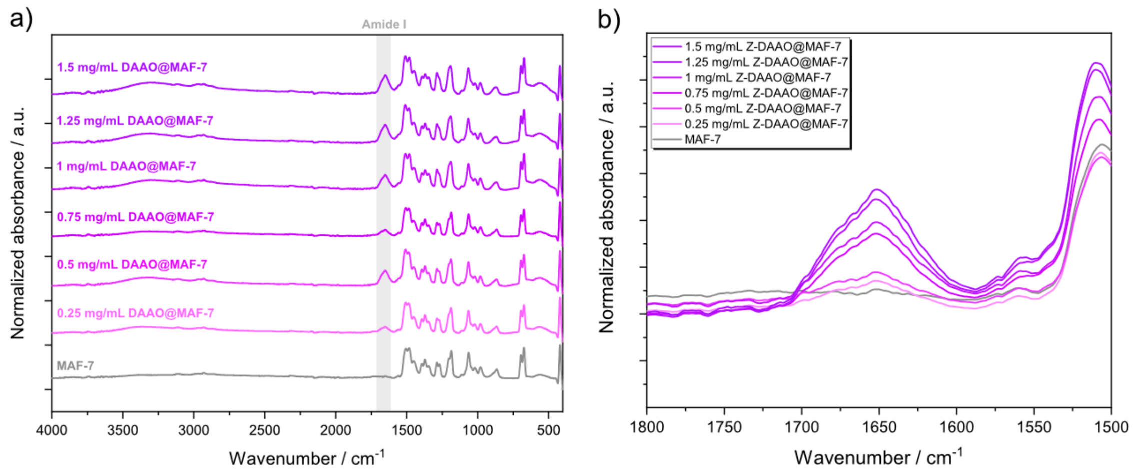

Figure S17. ATR-FTIR spectra of MAF-7 and Z-DAAO@MAF-7 with varied Z-DAAO concentration. (a) Spectra overview and (b) Detailed view of amid I region.

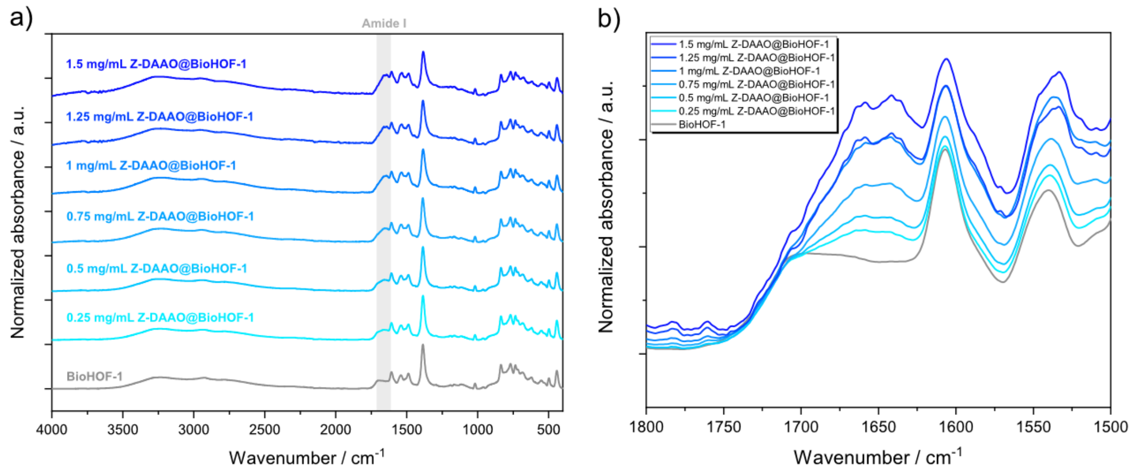

Figure S18. ATR-FTIR spectra of BioHOF-1 and Z-DAAO@BioHOF-1 with varied Z-DAAO concentration. (a) Spectra overview and (b) Detailed view of amid I region.

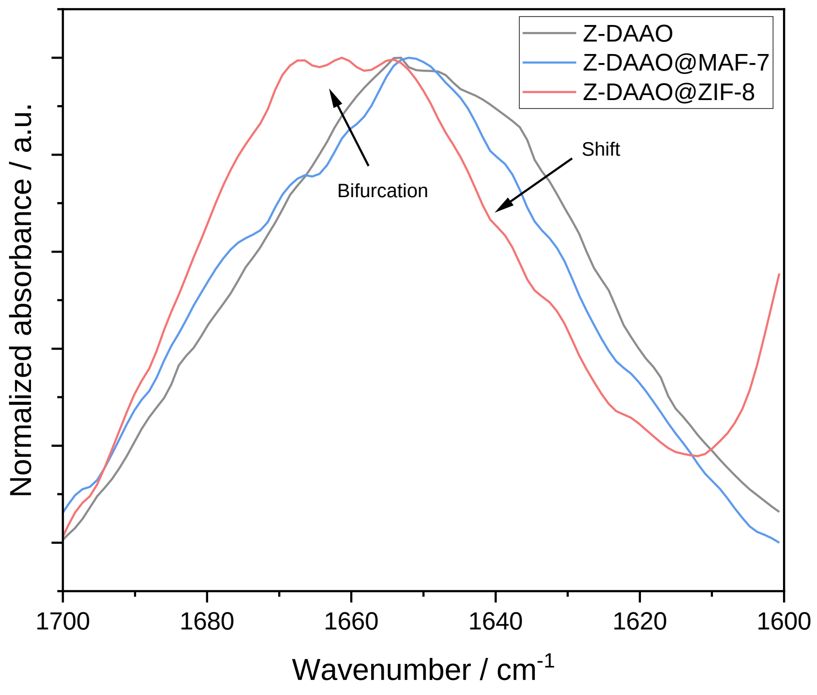

Figure S19. ATR-FTIR spectra of Z-DAAO, Z-DAAO@ZIF-8 and Z-DAAO@MAF-7. Shift in amide I peak, due to secondary structural changes and bifurcation of the amide I peak, coinciding with activity loss. Normalization of the amide I peak was performed.

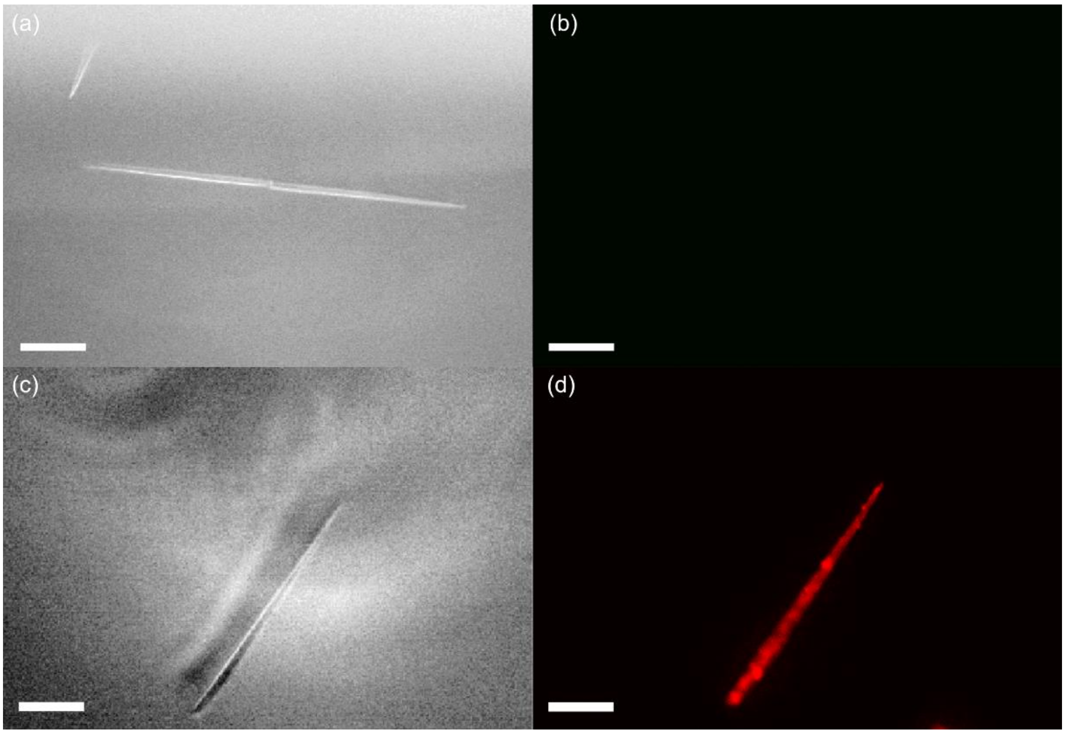

Figure S20. Bright-field and CLSM images of (a, b) BioHOF-1 and (c, d) Z-DAAO@BioHOF-1. The scale bar represents 10 µm.

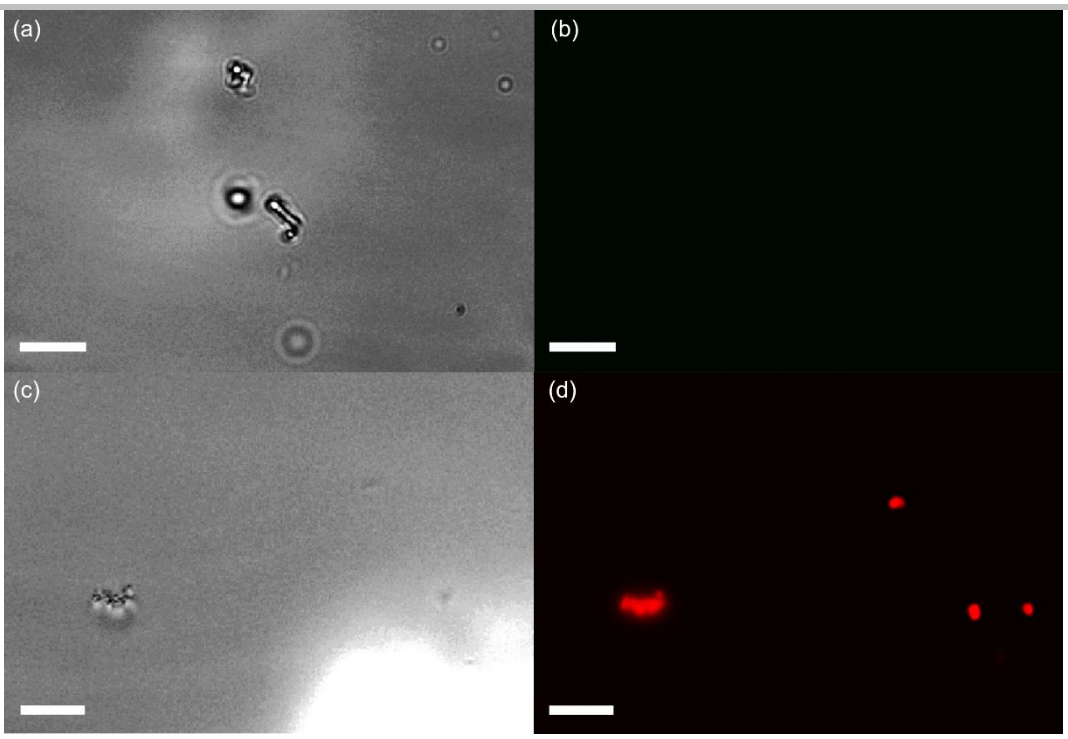

Figure S21. Bright-field and CLSM images of (a, b) ZIF-8 and (c, d) Z-DAAO@ZIF-8. The scale bar represents 10 µm.

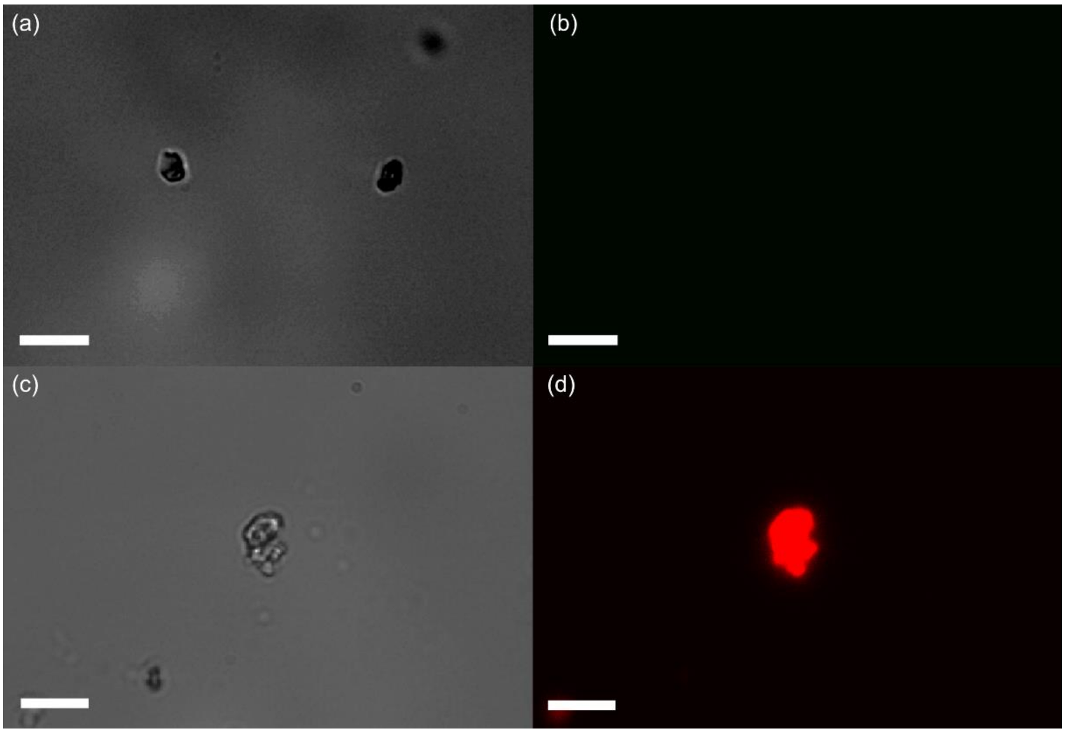

Figure S22. Bright-field and CLSM images of (a, b) MAF-7 and (c, d) Z-DAAO@MAF-7. The scale bar represents 10 µm.

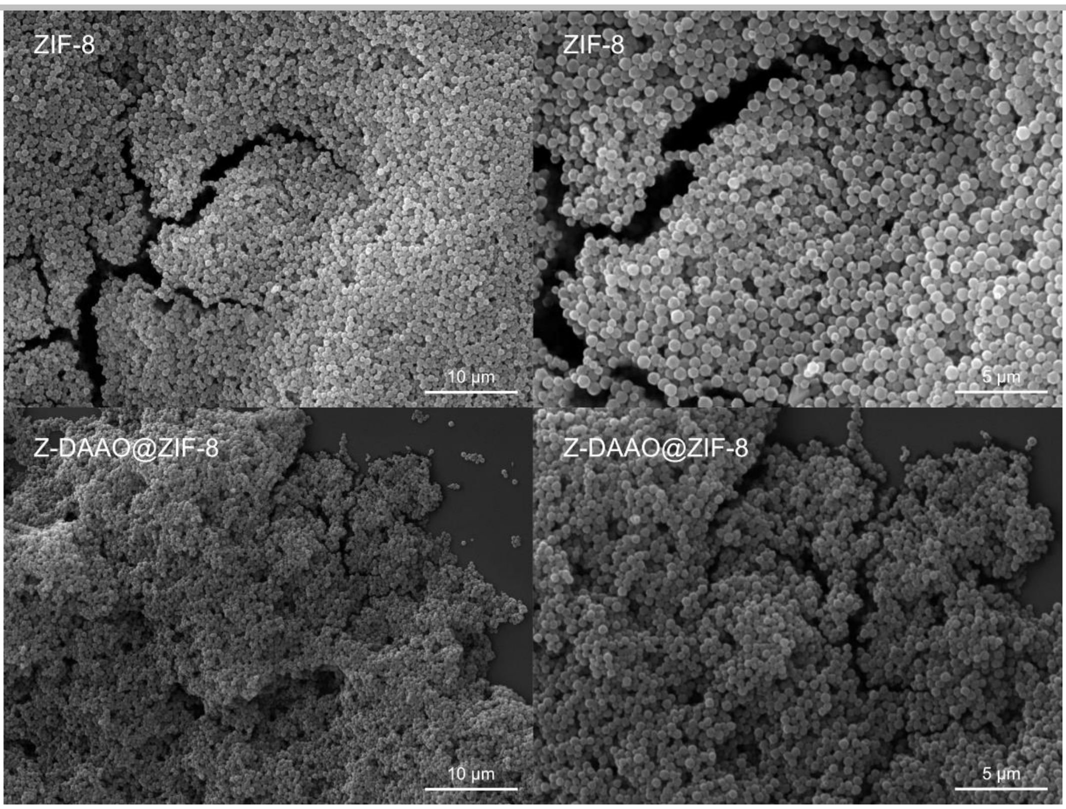

Figure S23. SEM images of ZIF-8 (top) and Z-DAAO@ZIF-8 (bottom).

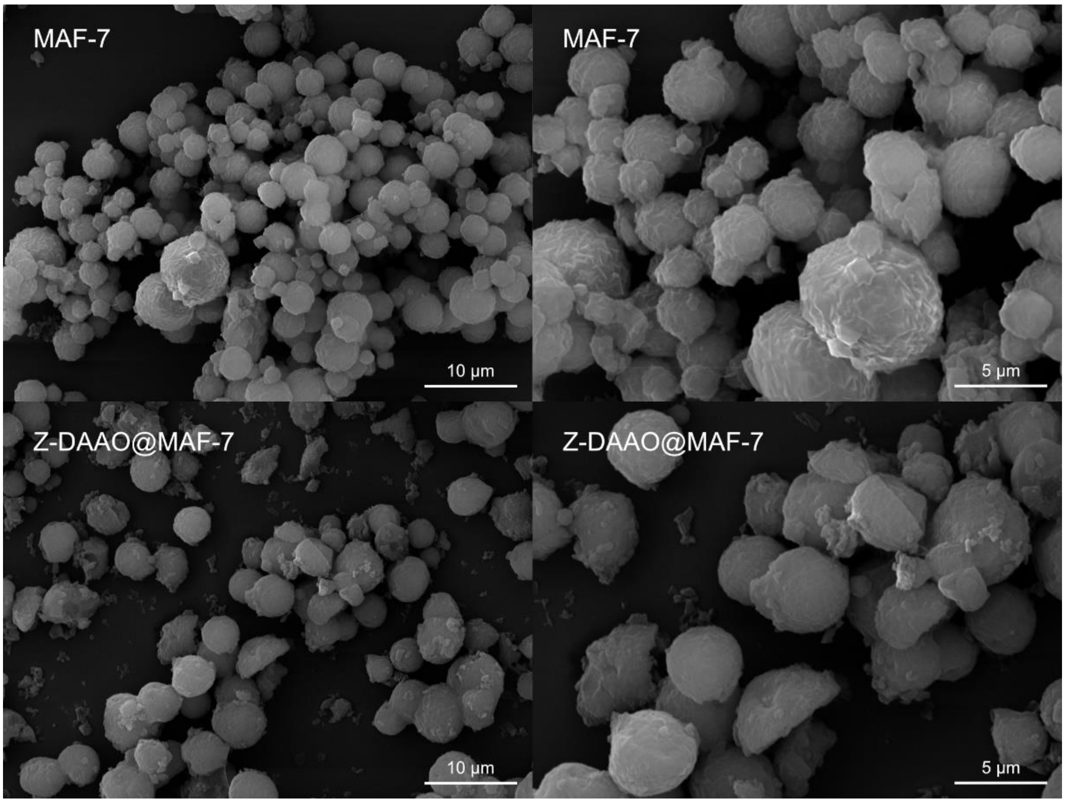

Figure S24. SEM images of MAF-7 (top) and Z-DAAO@MAF-7 (bottom).

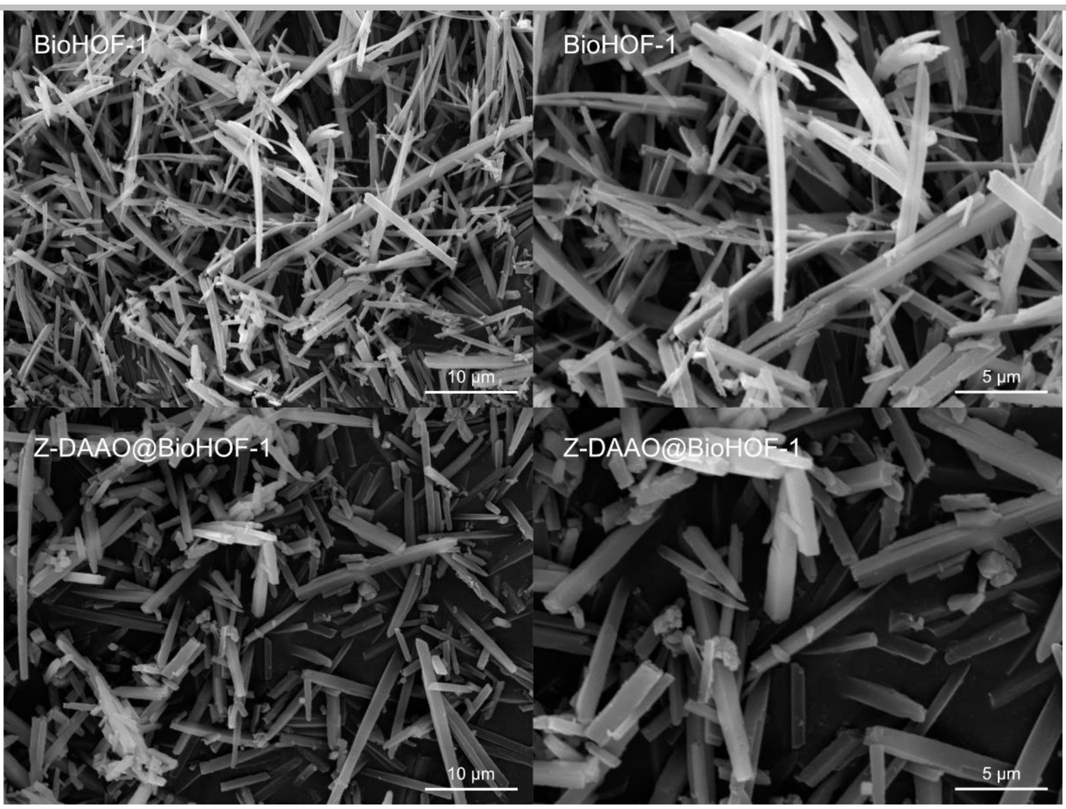

Figure S25. SEM images of BioHOF-1 (top) and Z-DAAO@BioHOF-1 (bottom).

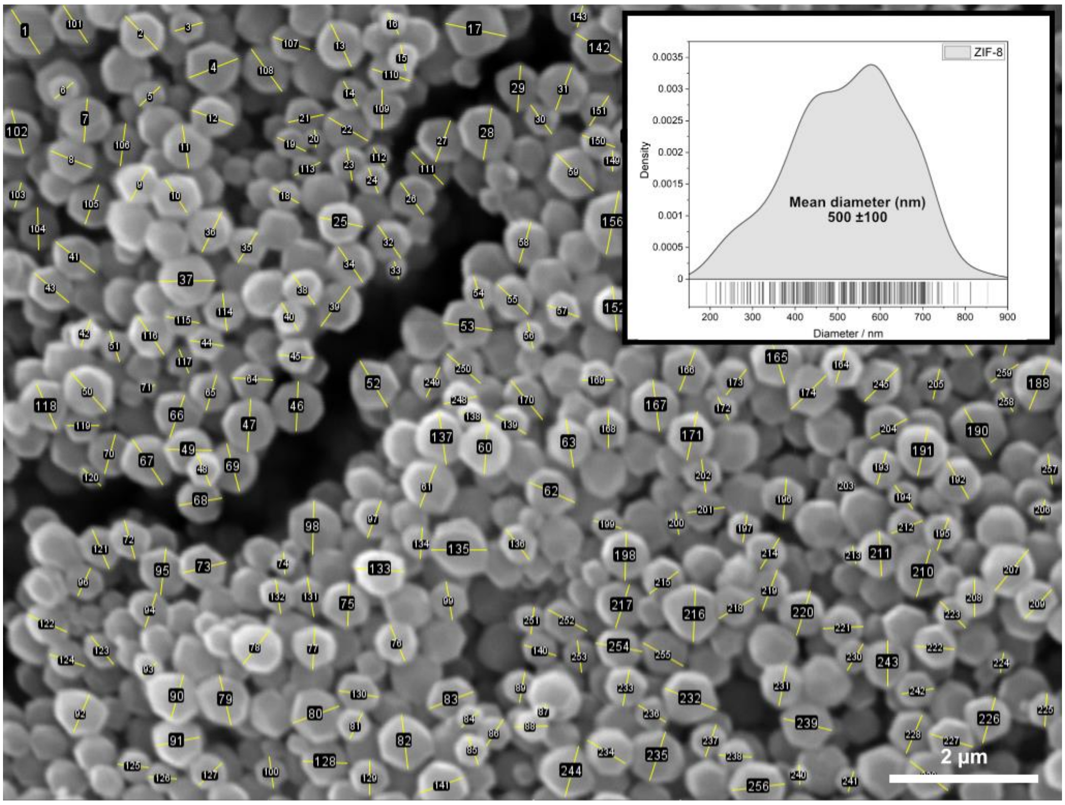

Figure S26. Particle size analysis of ZIF-8 with inset particle distribution density plot. Where ±…represents the standard deviation. Image analysis was performed with FIJI.[10]

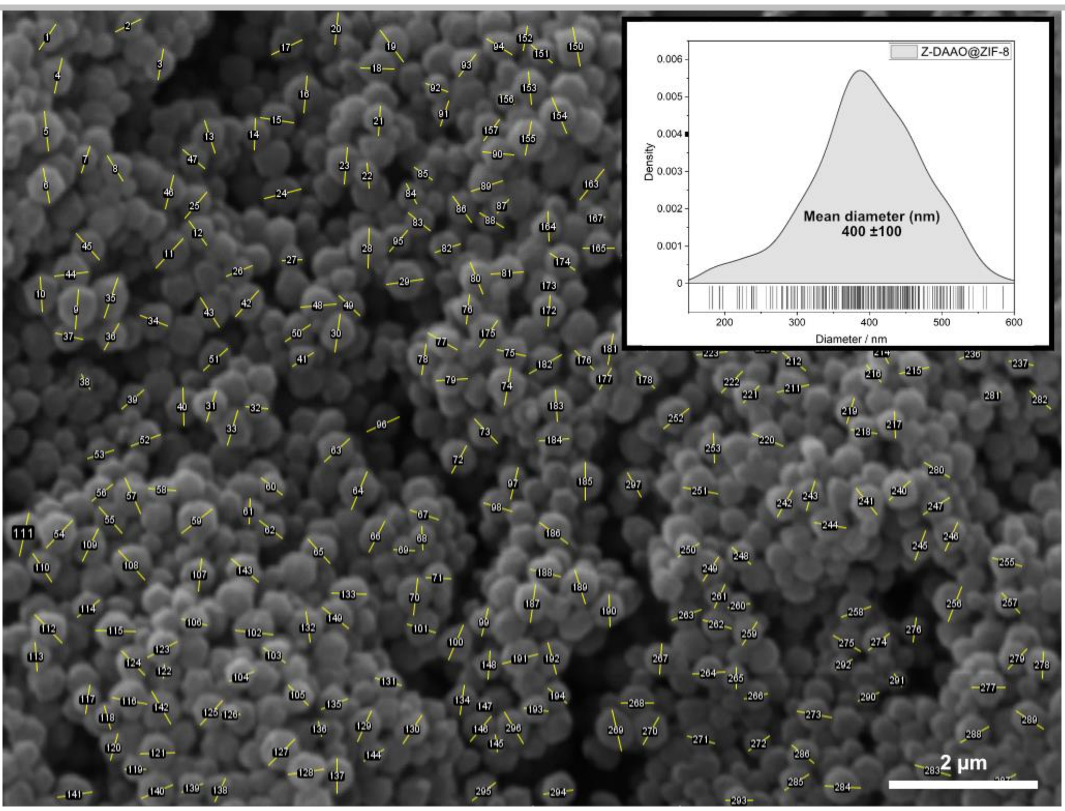

Figure S27. Particle size analysis of Z-DAAO@ZIF-8 with inset particle distribution density plot. Where ±…represents the standard deviation. Image analysis was performed with FIJI.[10]

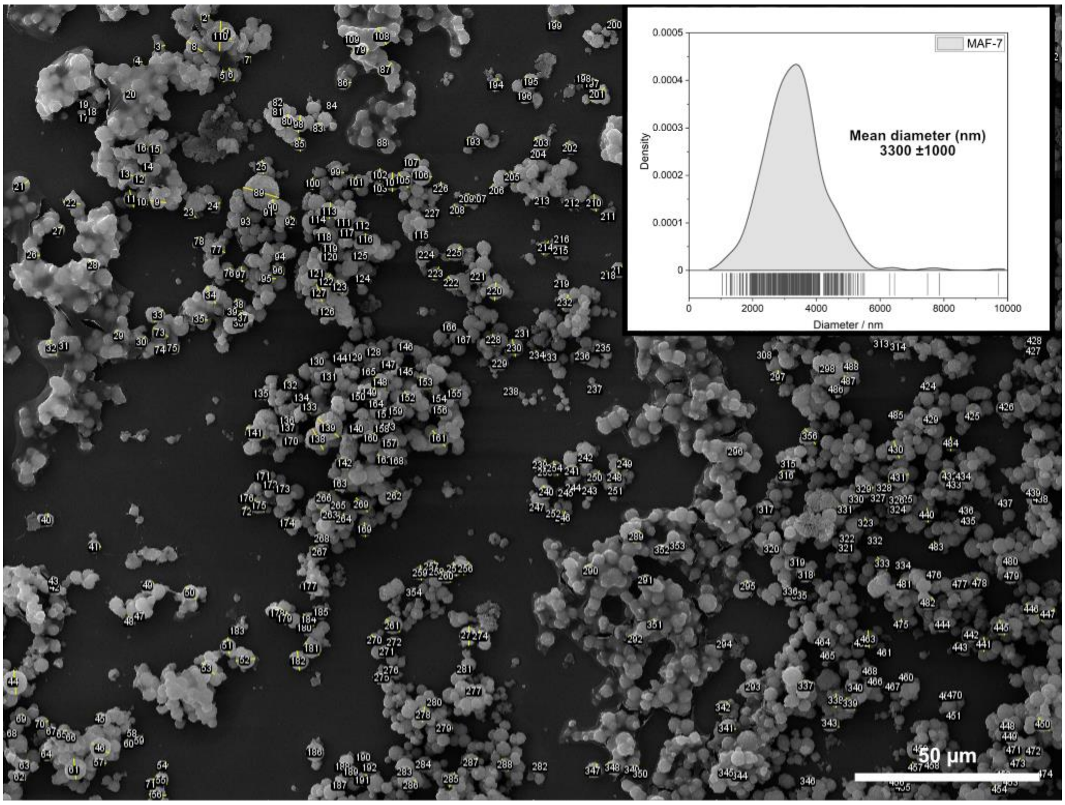

Figure S28. Particle size analysis of MAF-7 with inset particle distribution density plot. Where ±…represents the standard deviation. Image analysis was performed with FIJI.[10]

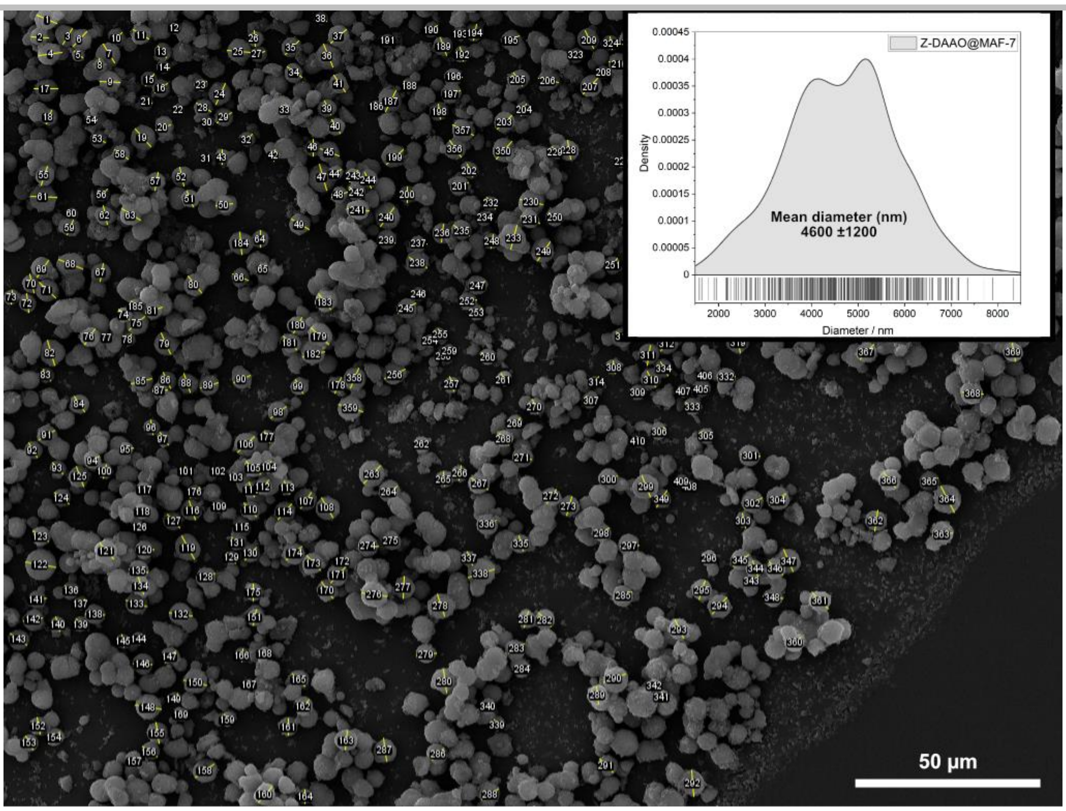

Figure S29. Particle size analysis of Z-DAAO@MAF-7 with inset particle distribution density plot. Where ±…represents the standard deviation. Image analysis was performed with FIJI.[10]

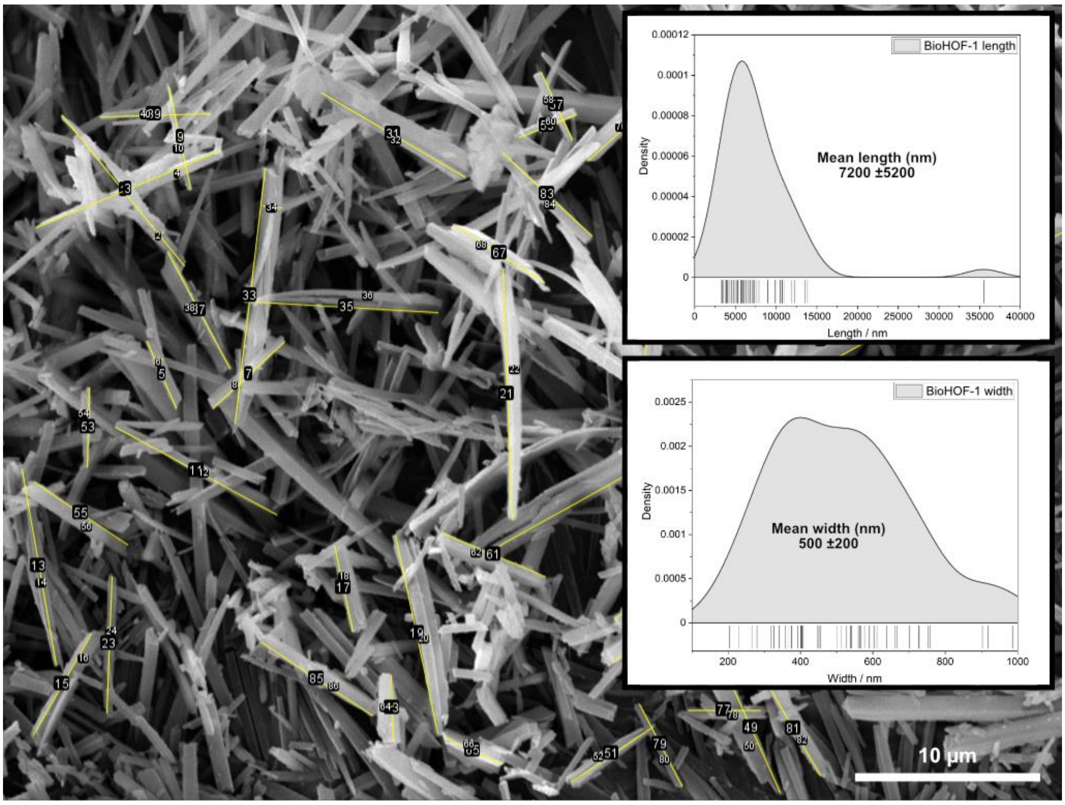

Figure S30. Particle size analysis of BioHOF-1 with inset particle distribution density plot. Where ±…represents the standard deviation. Image analysis was performed with FIJI.[10]

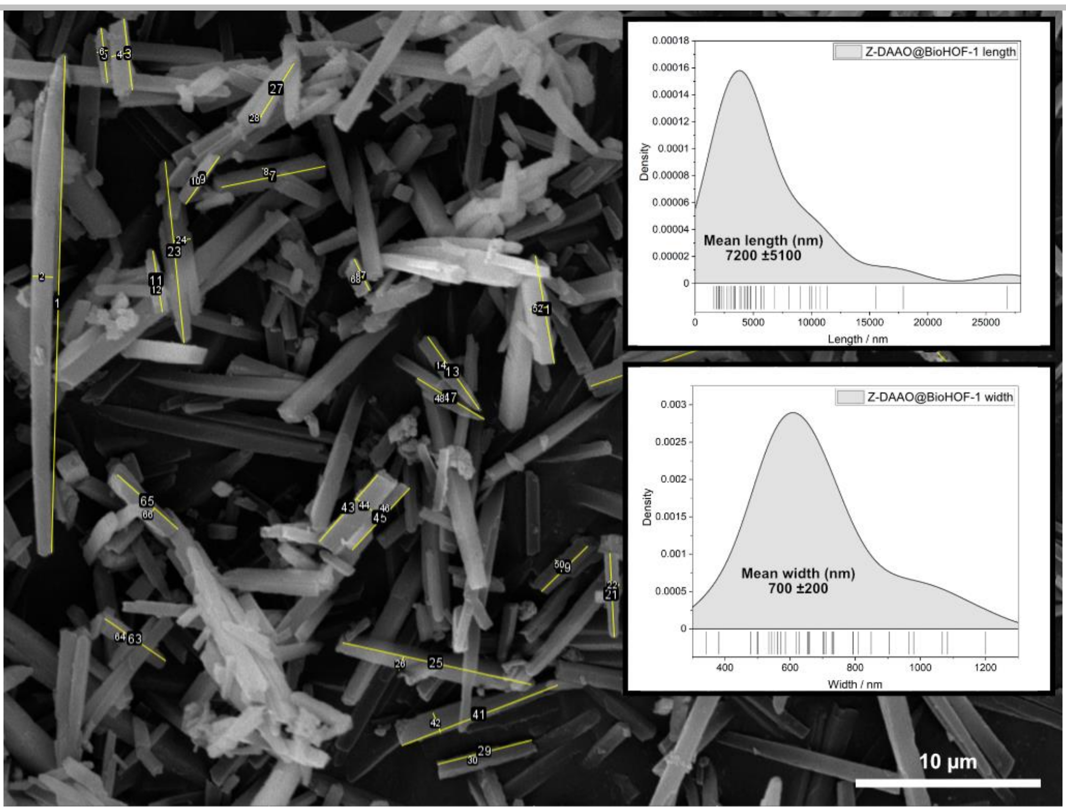

Figure S31. Particle size analysis of Z-DAAO@BioHOF-1 with inset particle distribution density plot. Where ±…represents the standard deviation. Image analysis was performed with FIJI.[10]

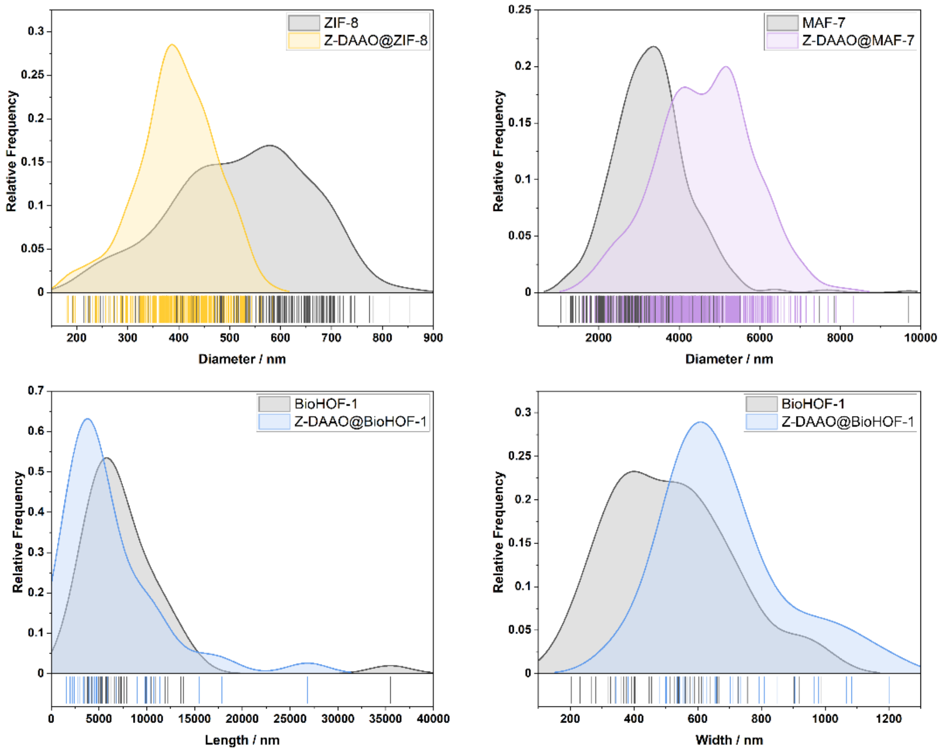

Figure S32. Comparison of the particle size distribution. (a) particle diameter of ZIF-8 and Z-DAAO@ZIF-8, (b) particle diameter of MAF-7 and Z-DAAO@MAF-7, (c) particle length of BioHOF-1 and Z-DAAO@BioHOF-1, and (d) particle width of BioHOF-1 and Z-DAAO@BioHOF-1.

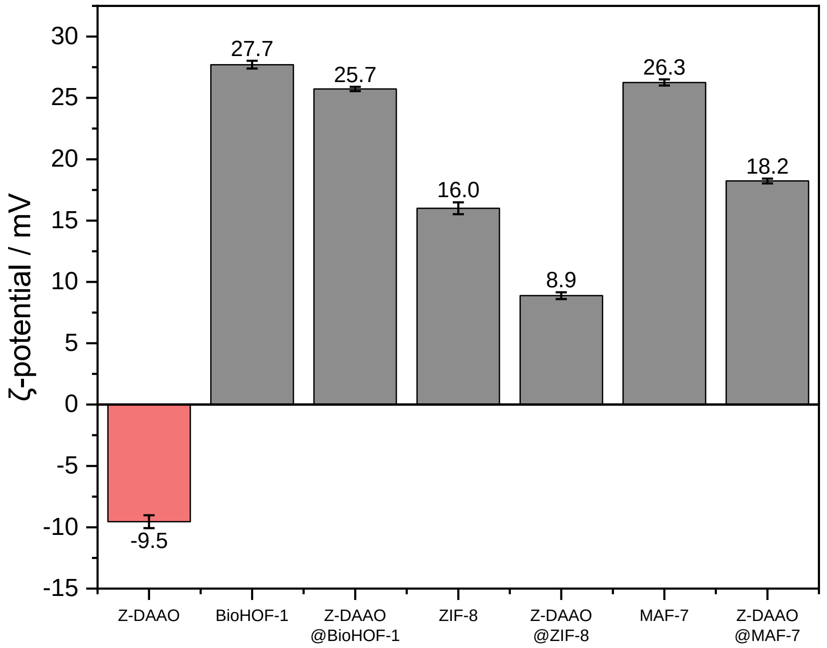

Figure S33. ζ-potentials of soluble Z-DAAO, each material without Z-DAAO (BioHOF-1, ZIF-8, MAF-7) and each biocomposite (Z-DAAO@BioHOF-1, Z-DAAO@ZIF-8, Z-DAAO@MAF-7).

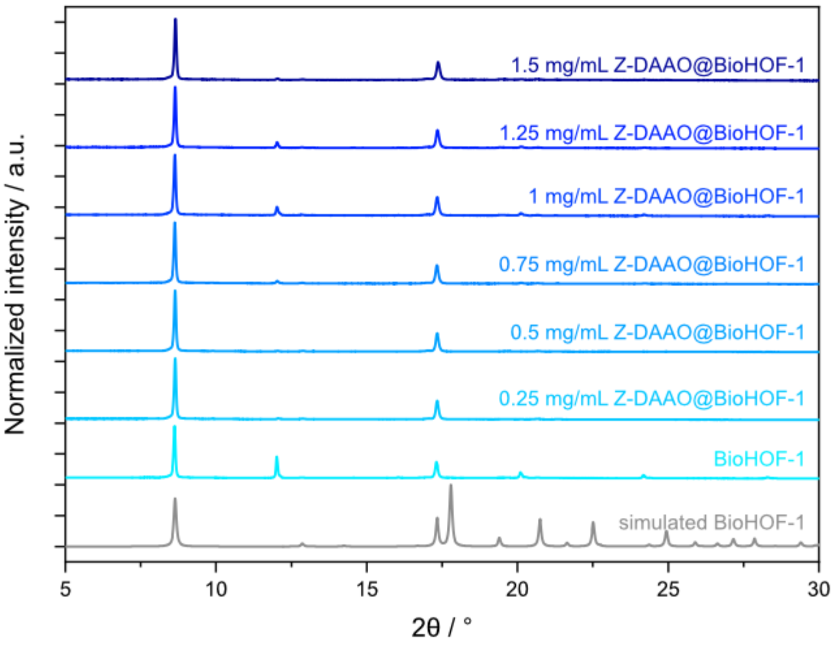

Figure S34. PXRD patterns of BioHOF-1 and Z-DAAO@BioHOF-1 with varied Z-DAAO concentrations, 4 mM (methanetetrayltetrakis(benzene-4,1-diyl))tetrakis(aminomethaniminium) and 3 mM 4,4',4'',4'''-methanetetrayltetrabenzoate during the HOF synthesis.

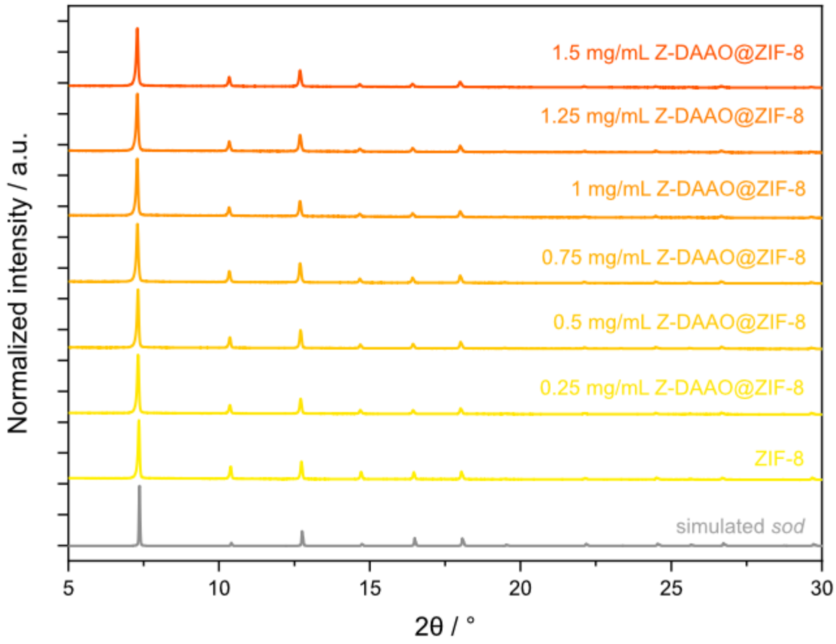

Figure S35. PXRD patterns of ZIF-8 and Z-DAAO@ZIF-8 with varied Z-DAAO concentrations, 40 mM $Zn(OAc)_2$ and 1280 mM 2-methyl-1H-imidazole during the MOF synthesis.

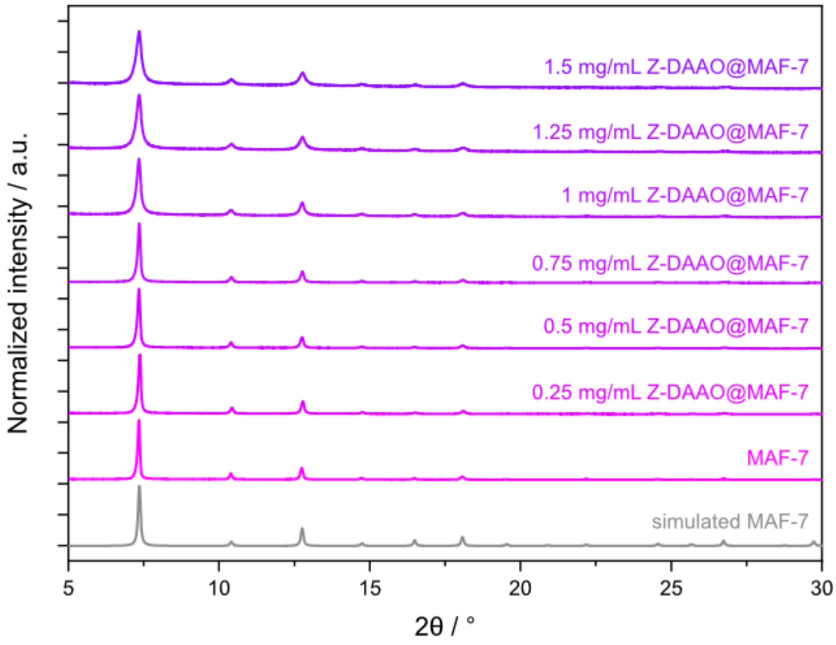

Figure S36. PXRD patterns of MAF-7 and Z-DAAO@MAF-7 with varied Z-DAAO concentrations, 40 mM $Zn(NO_3)_2{\cdot}6H_2O$ and 120 mM 2-methyl-1,2,3-triazole during the MOF synthesis.

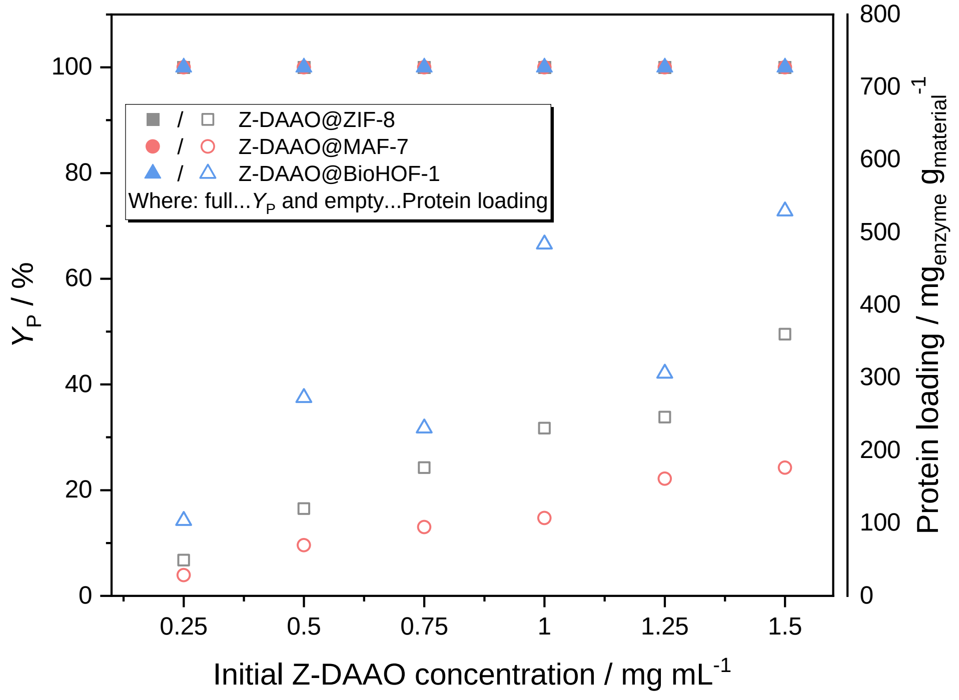

Figure S37. Protein yield (Y_P) and Protein loading at different initial Z-DAAO concentrations during the one-pot immobilization of Z-DAAO@ZIF-8, Z-DAAO@MAF-7 and Z-DAAO@BioHOF-14.

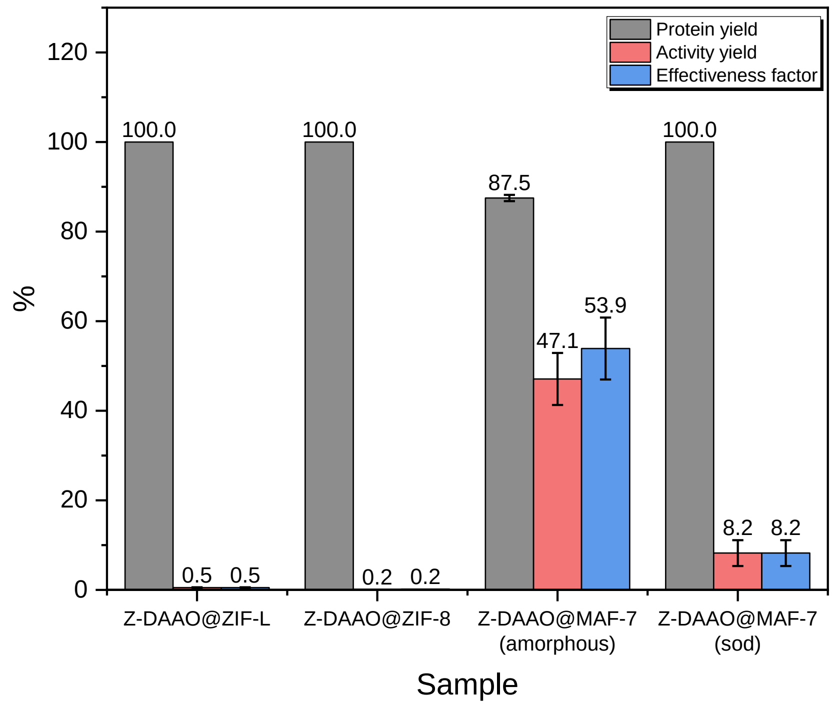

Figure S38. Immobilization performance of Z-DAAO@ZIF-L, Z-DAAO@ZIF-8, Z-DAAO@MAF-7(amorphous) and Z-DAAO@MAF-7(sod).

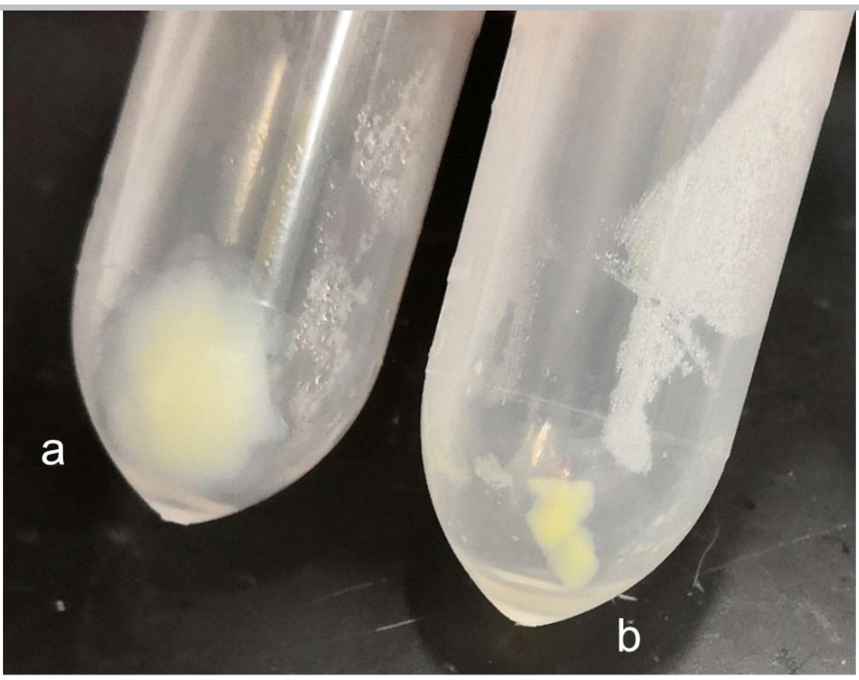

Figure S39. Z-DAAO@MAF-7(amorphous) (a) before treatment with 10 mM D-methionine (b) after treatment with 10 mM D-methionine.

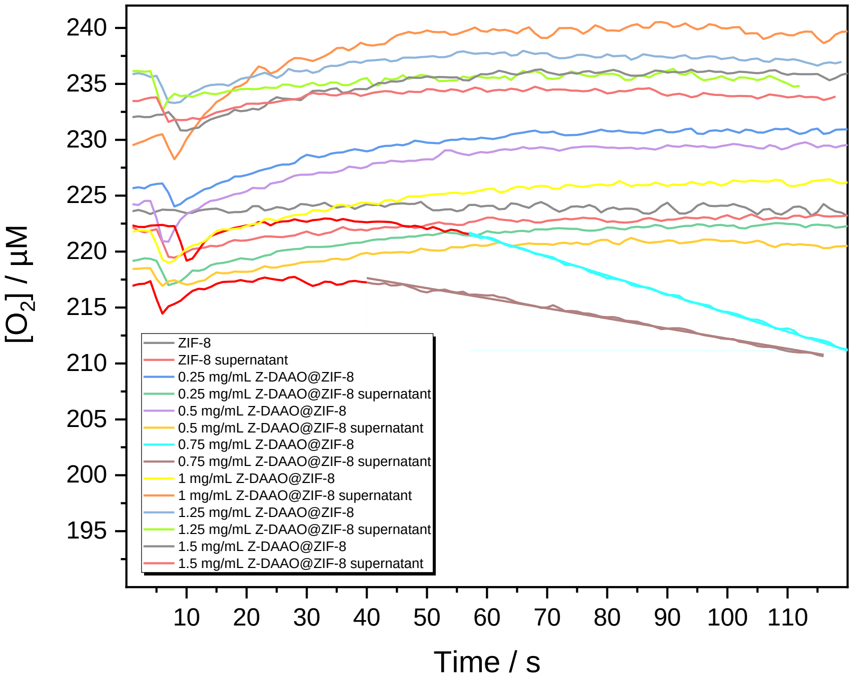

Figure S40. Oxygen time courses of varying concentrations of Z-DAAO@ZIF-8 and the supernatant after immobilization. Linear regression after an initial equilibration period (see I10 DAAO Activity assay).

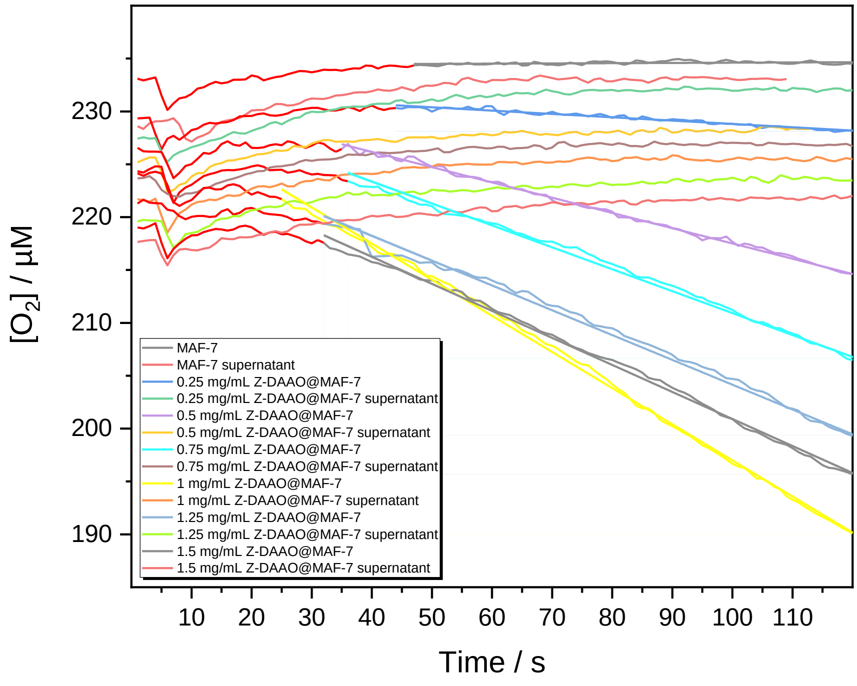

Figure S41. Oxygen time courses of varying concentrations of Z-DAAO@MAF-7 and the supernatant after immobilization. Linear regression after an initial equilibration period (see I10 DAAO Activity assay).

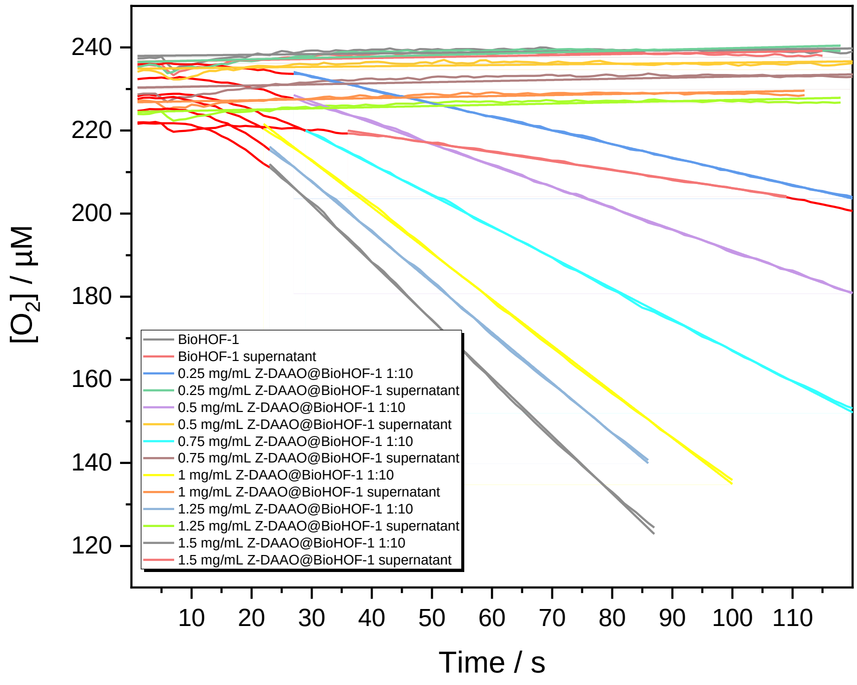

Figure S42. Oxygen time courses of varying concentrations of Z-DAAO@BioHOF-1 and the supernatant after immobilization. Linear regression after an initial equilibration period (see I10 DAAO Activity assay).

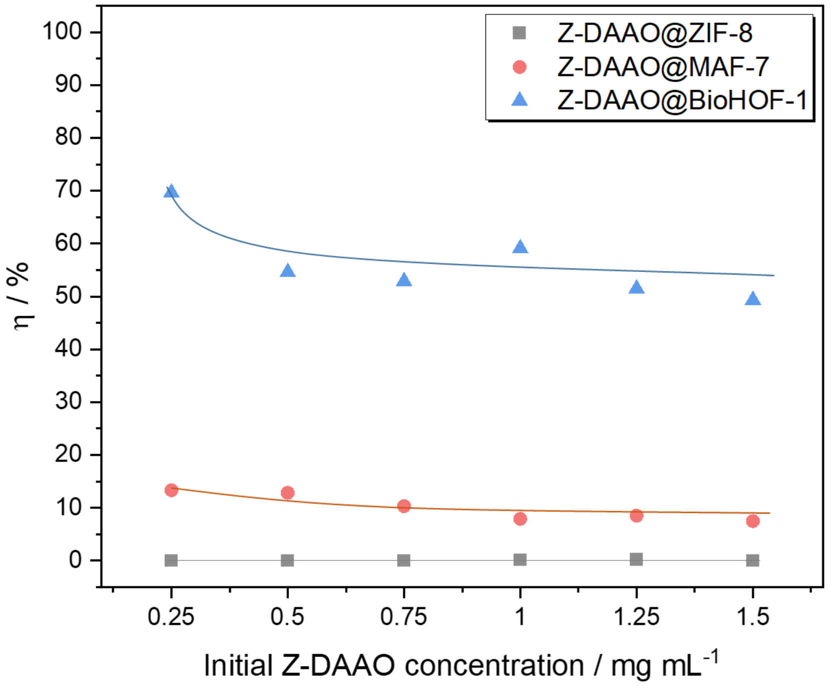

Figure S43. Effectiveness factor (η) at different initial Z-DAAO concentrations during the one-pot immobilization.

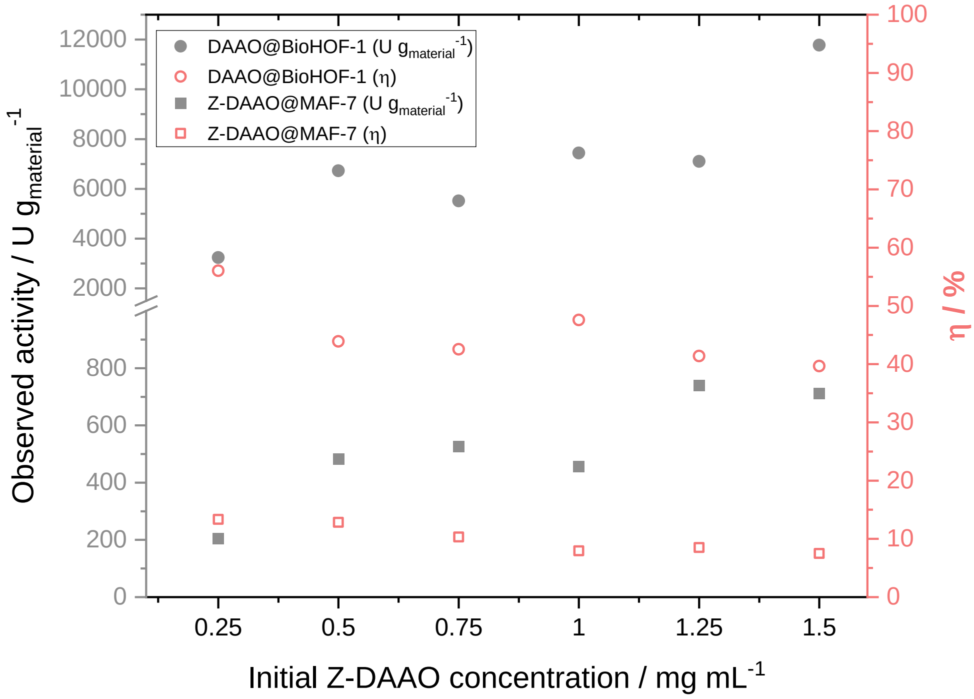

Figure S44. Comparison of observed activity (U $g_{material}^{-1}$) and effectiveness factor (η) for Z-DAAO@MAF-7 and Z-DAAO@BioHOF-1.

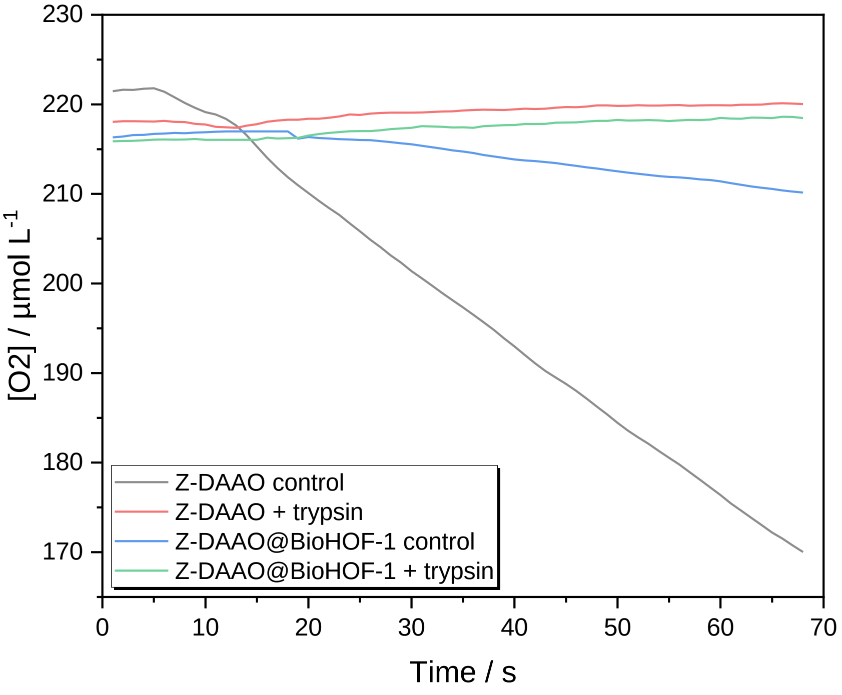

Figure S45. Oxygen time courses of Z-DAAO and Z-DAAO@BioHOF-1 with and without trypsin digestion incubated at 37°C for 1 h.

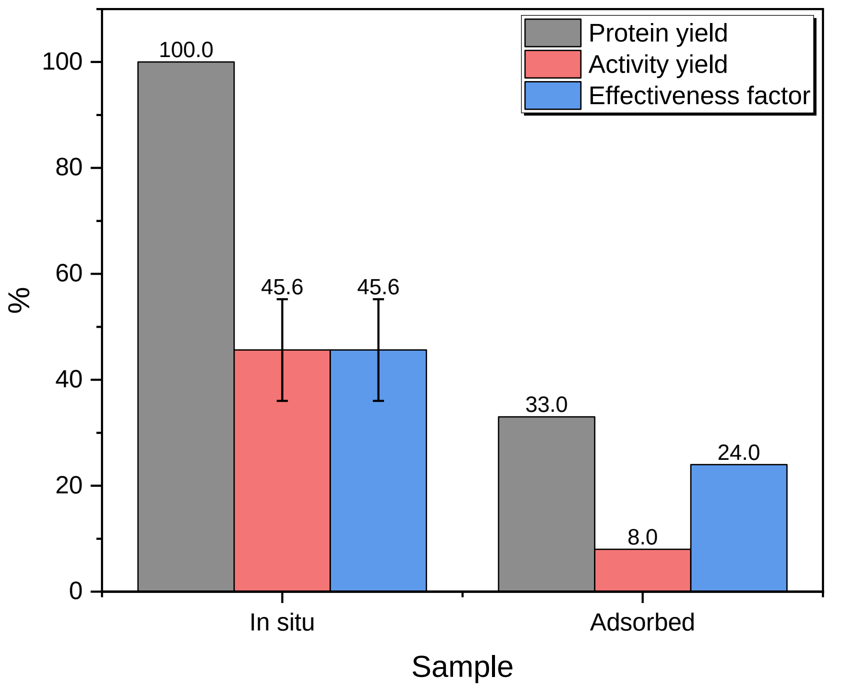

Figure S46. Immobilization performance of Z-DAAO@BioHOF-1 by in situ formation of the material in presence of the enzyme and after adsorption of Z-DAAO for 1 h on previously synthesized BioHOF-1.

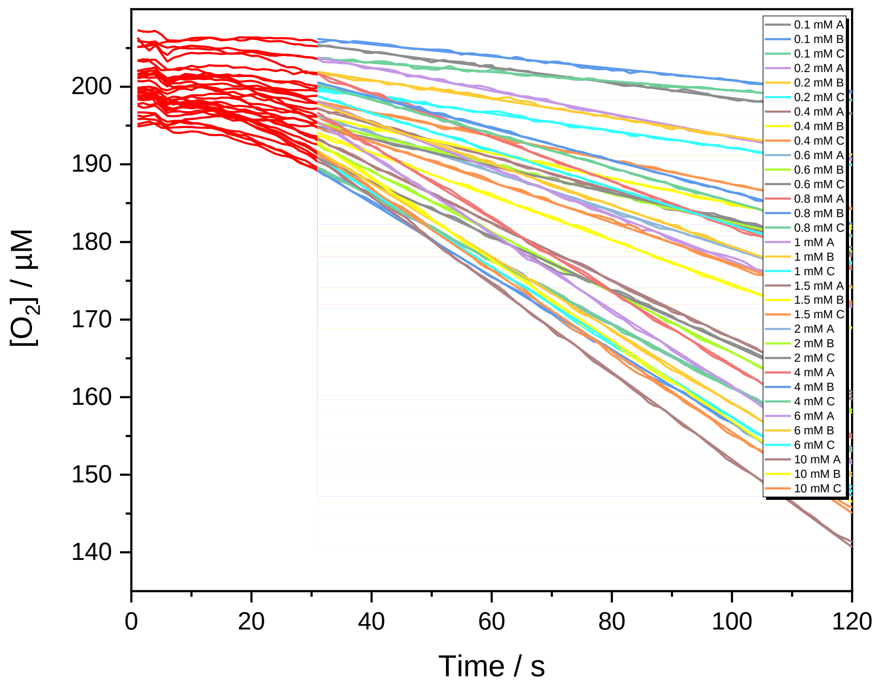

Figure S47. Oxygen time courses of Z-DAAO (28.54 µg/mL) at different D-methionine concentrations (0.1 – 10 mM) and fitted curves to determine the consumption rate. Linear regression after an initial equilibration period (see I10 DAAO Activity assay).

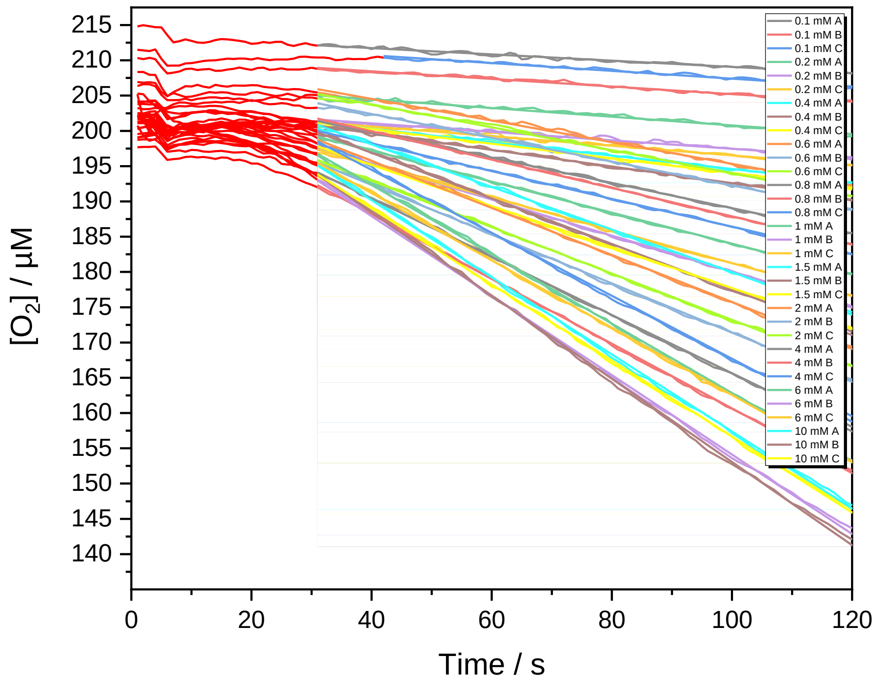

Figure S48. Oxygen time courses of Z-DAAO@MAF-7 (df=2) at different D-methionine concentrations (0.1 – 10 mM) and fitted curves to determine the consumption rate. Linear regression analysis was performed after an initial equilibration period (see I10 DAAO Activity assay).

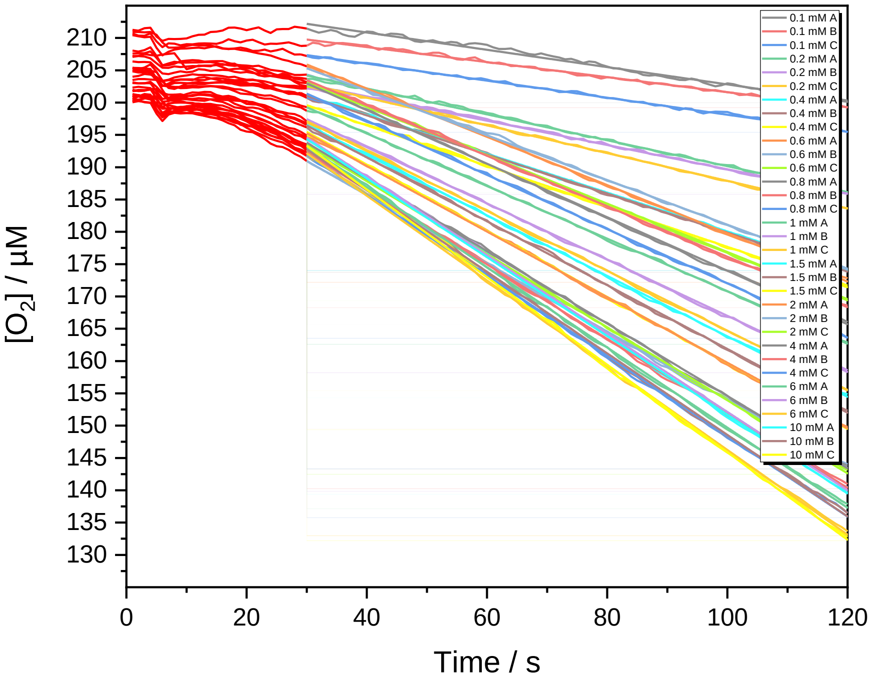

Figure S49. Oxygen time courses of Z-DAAO@BioHOF-1 (df=10) at different D-methionine concentrations (0.1 – 10 mM) and fitted curves to determine the consumption rate. Linear regression after an initial equilibration period (see I10 DAAO Activity assay).

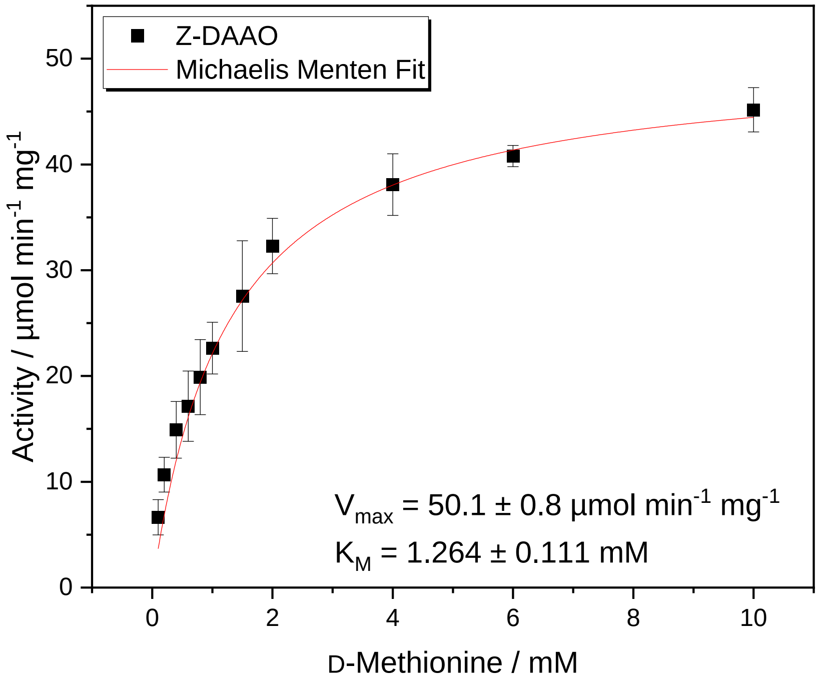

Figure S50. Michaelis-Menten plot of Z-DAAO activity measured at D-methionine concentrations between 0.1 – 10 mM.

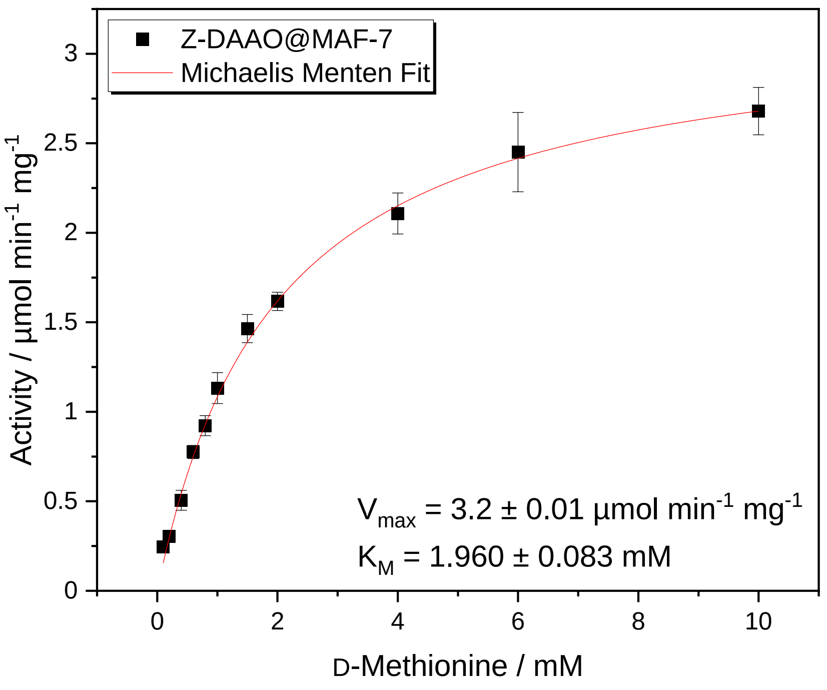

Figure S51. Michaelis-Menten plot of Z-DAAO@MAF-7 activity measured at D-methionine concentrations between 0.1 – 10 mM.

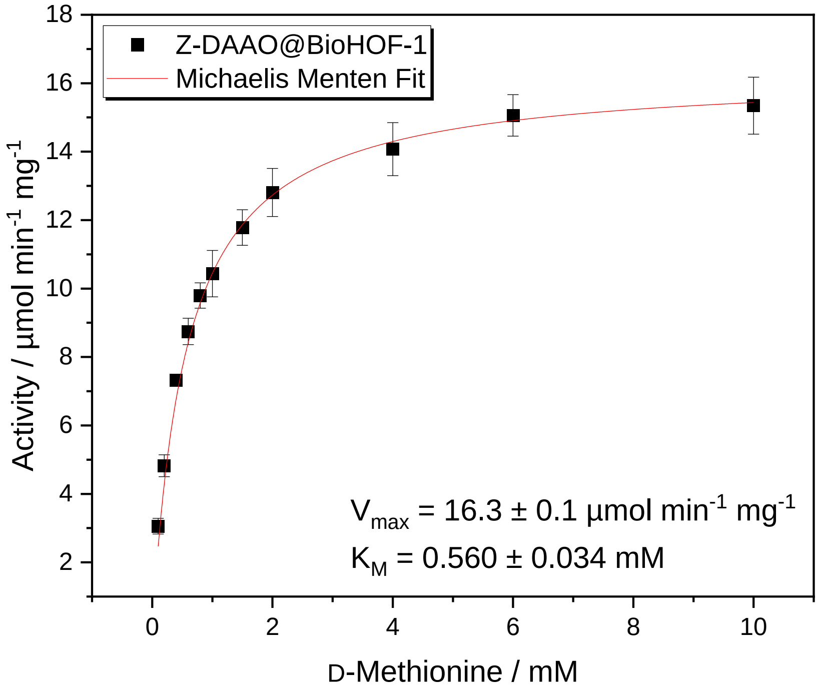

Figure S52. Michaelis-Menten plot of Z-DAAO@BioHOF-1 activity measured at D-methionine concentrations between 0.1 – 10 mM.

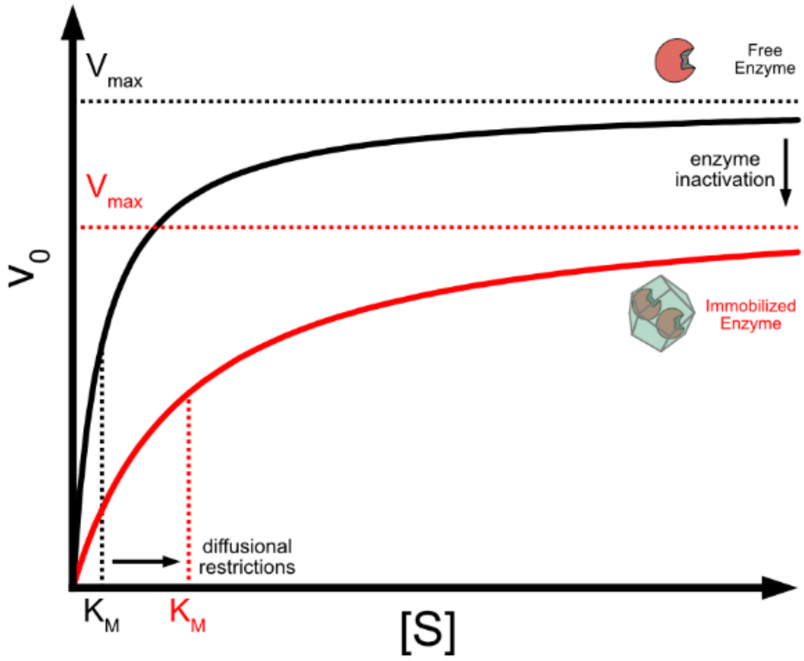

Figure S53. Illustration of the effects of immobilization on the kinetic parameters of an enzyme, following a Michaelis-Menten reaction order. Where v0 is the reaction rate and [S] the substrate concentration. Decreased Vmax can indicate biocatalyst inactivation, whereas an increase in KM can be attributed to an increase in diffusional restrictions.

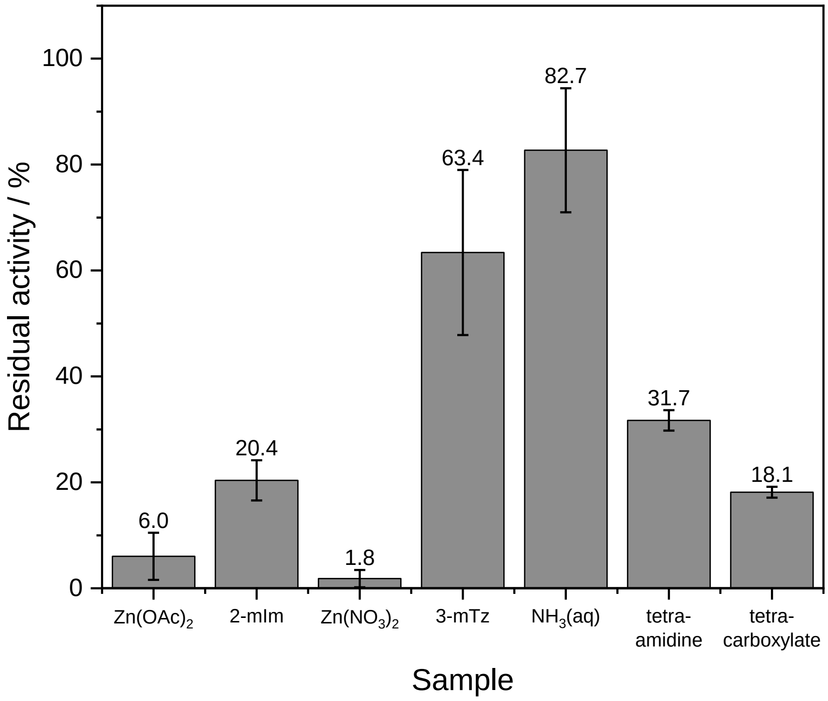

Figure S54. Precursor stability of Z-DAAO. 1 mg mL^{-1} Z-DAAO was incubated in the presence of each framework precursor (at concentrations used in the immobilization procedure) at 22°C for 1h (750 rpm).

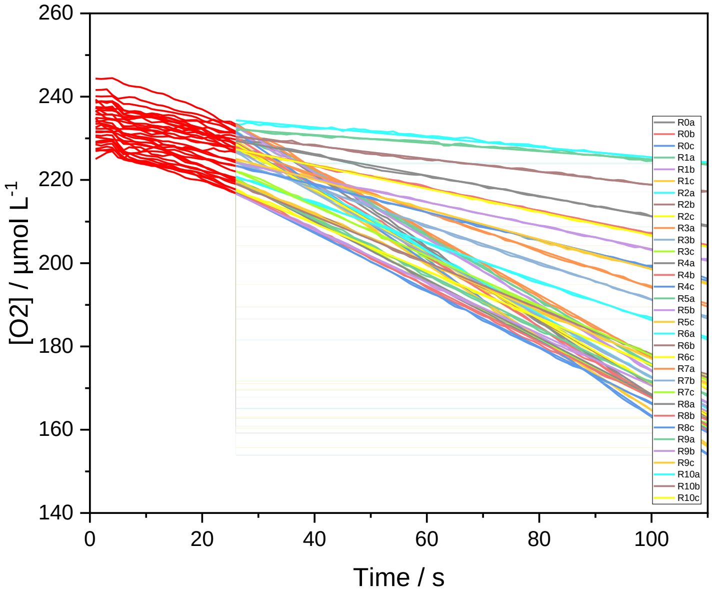

Figure S55. Oxygen time courses of Z-DAAO@MAF-7 after recycling. The reaction was performed with 20 $mg_{wet\ weight}$ mL^{-1} biocomposite, 10 mM D-methionine and 20 mM HEPES (pH 8) at 30°C after each the biocomposite was separated by centrifugation and reused in a fresh reaction mixture. Linear regression after an initial equilibration period (see I10 DAAO Activity assay).

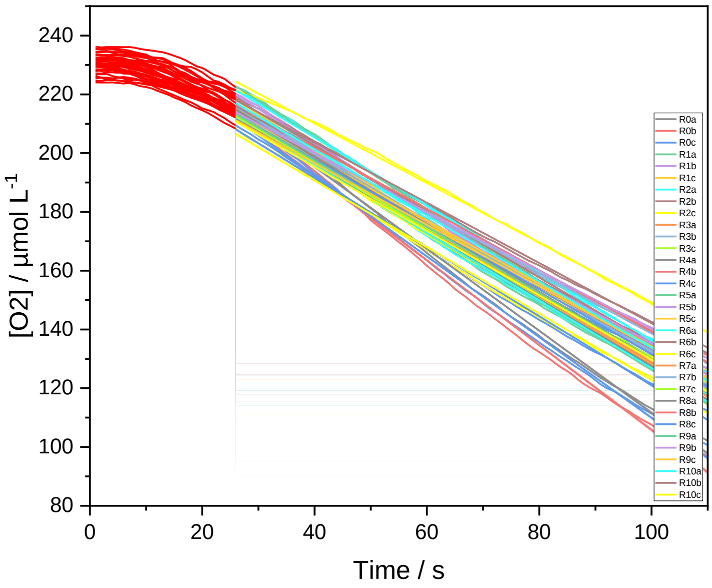

Figure S56. Oxygen time courses of Z-DAAO@BioHOF-1 after recycling. The reaction was performed with 20 $mg_{wet\ weight}$ mL^{-1} biocomposite, 10 mM D-methionine and 20 mM HEPES (pH 8) at 30°C after each the biocomposite was separated by centrifugation and reused in a fresh reaction mixture. Linear regression after an initial equilibration period (see I10 DAAO Activity assay).

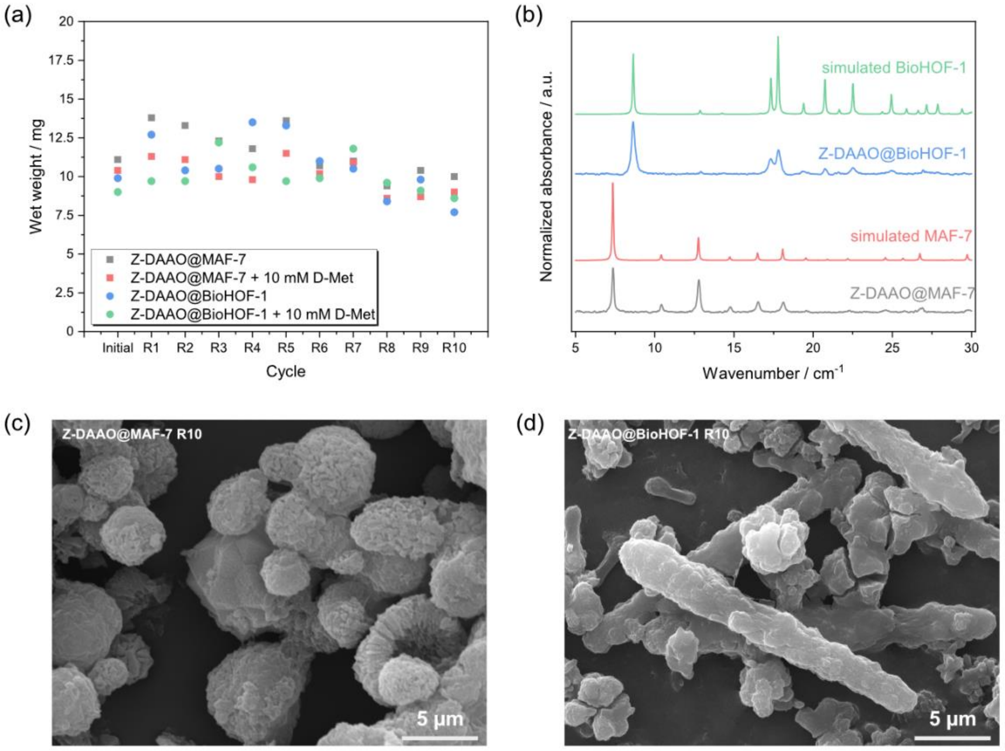

Figure S57. Characterization of Z-DAAO@MAF-7 and Z-DAAO@BioHOF-1 after recycling. (a) Stability test of Z-DAAO@MAF-7 and Z-DAAO@BioHOF-1: wet weight after incubation in 20 mM HEPES (pH 8) or 20 mM HEPES (pH 8) + 10 mM D-met for 2 min at 30°C. (b) PXRD of Z-DAAO@MAF-7 and Z-DAAO@BioHOF-1 after 10 cycles. (c-d) SEM of Z-DAAO@MAF-7 and Z-DAAO@BioHOF-1 after 10 cycles.

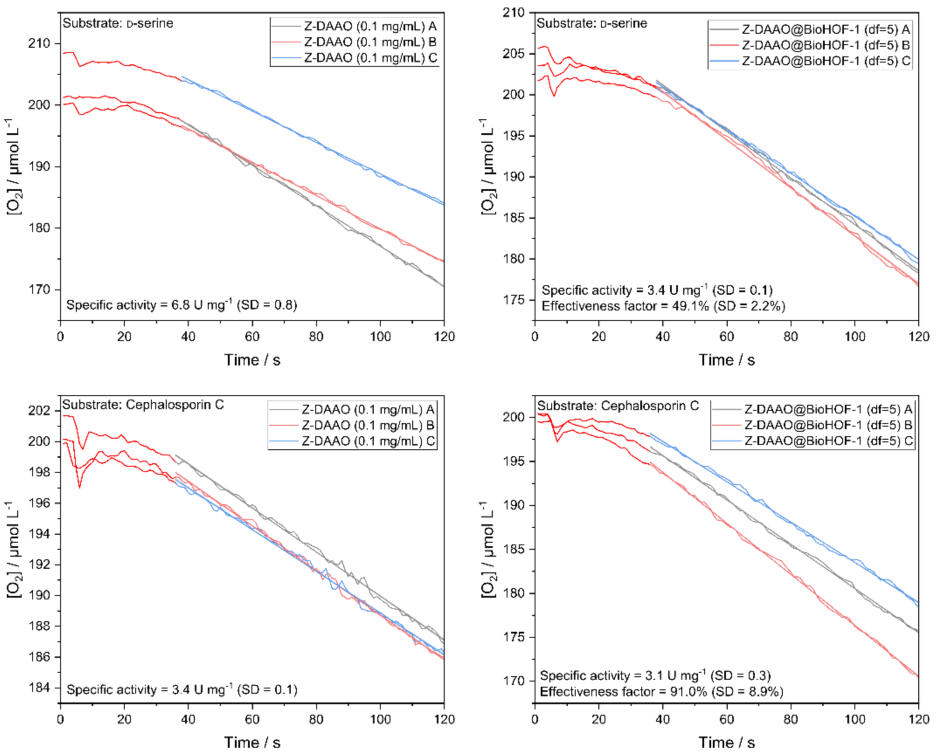

Figure S58. Activity of Z-DAAO and Z-DAAO@BioHOF-1 with 10 mM D-serine (a, b) and 10 mM cephalosporin C (c, d) as substrate.

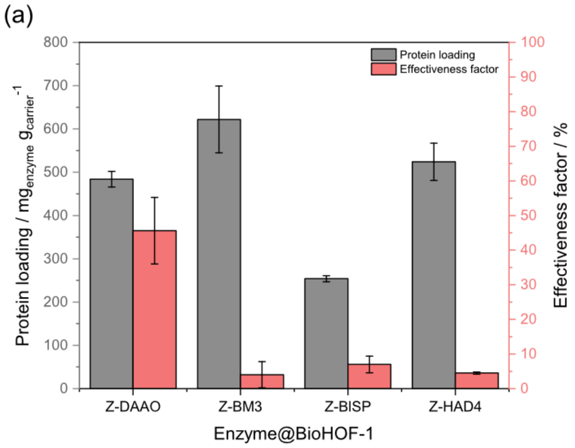

Figure S59. Extension of BioHOF-1 immobilization approach to P450 BM3, BlSP and HAD4. (a) Protein loading and effectiveness factor of Z-DAAO, Z-BM3, Z-BlSP and Z-HAD4 @BioHOF-1. (b) Comparison of protein loading of enzymes harboring the Z$_{basic2}$ binding module and Strep II (DAAO) or His-tagged (BM3, BlSP) variants.

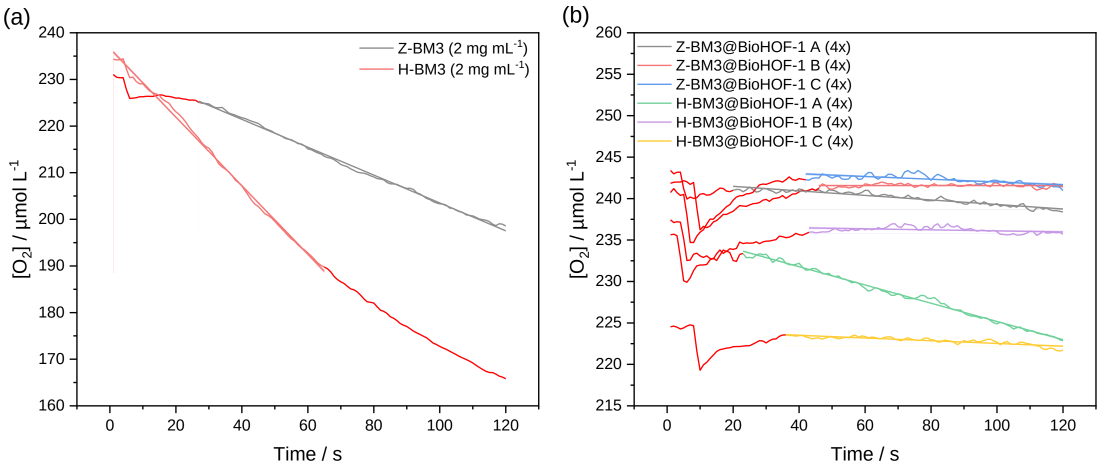

Figure S60. Oxygen time courses of (a) Z/H-BM3 and (b) Z/H-BM3@BioHOF-1 during the conversion of lauric acid (2 mM) at 30°C, pH 7.4.

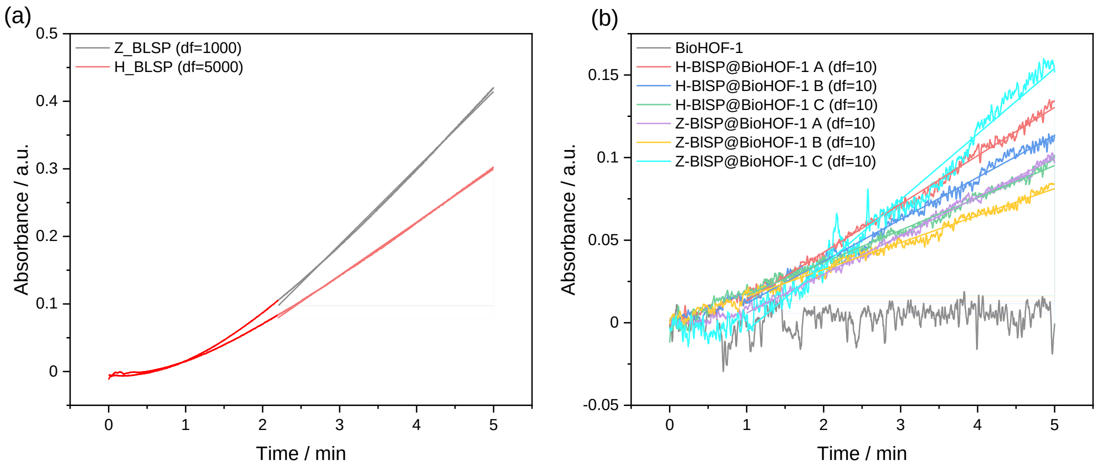

Figure S61. Formation of NADH in a cascade reaction containing BlSP, phosphoglucomutase and glucose-6-phosphate dehydrogenase during the conversion of sucrose. (a) Z/H-BlSP and (b) Z/H-BlSP@BioHOF-1. The reaction was performed at 30°C, pH 7.4, in a spectrophotometer fitted with a magnetic stirrer.

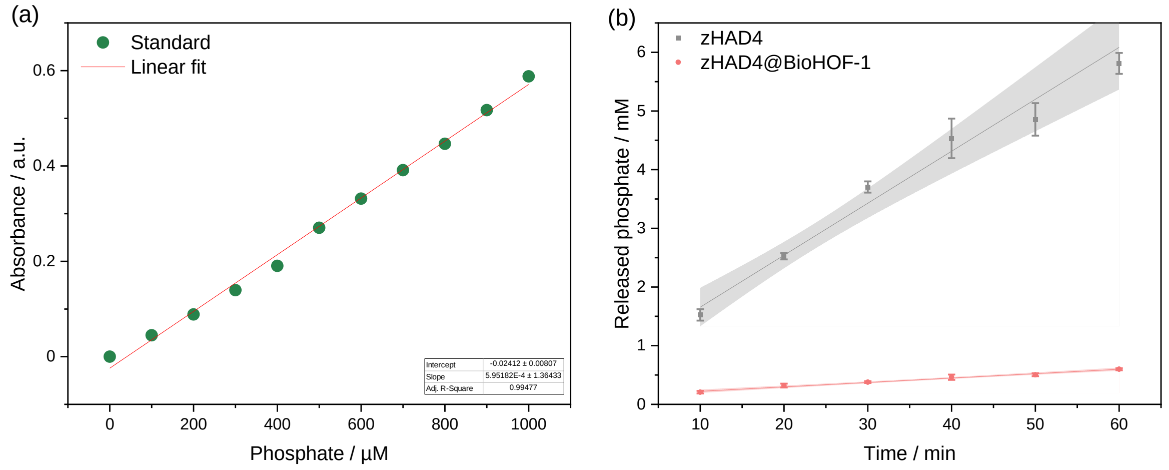

Figure S62. (a) Example calibration curve for free phosphate measured per microwell plate. (b) Released phosphate for Z-HAD4 and Z-HAD4@BioHOF-1.

Table S1. Comparison of protein loading for Z-BM3, Z-BlSP and Z-HAD4 immobilized with BioHOF-1 (this work) and different porous carriers, previously reported in literature.

Enzyme	Preparation	Material	Protein loading (mg_{enzyme} $g_{carrier}^{-1}$)	Specific activity$_{material}$ (U $g_{material}^{-1}$)	Reference
Z-BM3	Purified enzyme	BioHOF-1	622.0	9.6 (η = 0.04)	Our work
Z-BM3	Cell-free extract	anionic sulfopropyl - activated carrier (ReliSorb SP400)	12.7	5.0[a]	[4]
Z-BlSP	Purified enzyme	BioHOF-1	254.0	663.0 (η = 0.07)	Our work
Z-BlSP	Cell-free extract	anionic sulfopropyl - activated carrier (ReliSorb SP400)	1.6	59.1[a]	[18]
Z-HAD4	Purified enzyme	BioHOF-1	524.0	19.7 (η = 0.05)	Our work
Z-HAD4	Cell-free extract	Fractogel EMD SO_3^-	12.0 - 18	10.0 - 15.0[a]	[7]
Z-HAD4	Cell-free extract	Relisorb SP400	12.0 - 18	10.0 - 15.0[a]	[7]

[a] Theoretical specific activity of each material, calculated from the specific activity of each enzyme obtained in this work (Z-BM3 = 0.4 U mg^{-1}, Z-BlSP = 38.1 U mg^{-1}, Z-HAD4 = 0.8 U mg^{-1}) and the reported protein loading of each material. Theoretical specific activities of each material were calculated assuming an ideal effectiveness factor (η = 1).

References

[1] T. G. Schmidt, A. Skerra, *Nat Protoc* **2007**, *2*, 1528–1535.
[2] M. Hedhammar, S. Hober, *J. Chromatogr. A* **2007**, *1161*, 22–28.
[3] I. Dib, D. Stanzer, B. Nidetzky, *Appl. Environ. Microbiol.* **2007**, *73*, 331–333.
[4] D. Valikhani, J. M. Bolivar, A. Dennig, B. Nidetzky, *Biotechnol. Bioeng.* **2018**, *115*, 2416–2425.
[5] J. Wiesbauer, J. M. Bolivar, M. Mueller, M. Schiller, B. Nidetzky, *ChemCatChem* **2011**, *3*, 1299–1303.
[6] C. Goedl, A. Schwarz, A. Minani, B. Nidetzky, *J. Biotechnol.* **2007**, *129*, 77–86.
[7] M. Pfeiffer, P. Wildberger, B. Nidetzky, *J. Mol. Catal. B: Enzym.* **2014**, *110*, 39–46.
[8] W. Liang, F. Carraro, M. B. Solomon, S. G. Bell, H. Amenitsch, C. J. Sumby, N. G. White, P. Falcaro, C. J. Doonan, *J. Am. Chem. Soc.* **2019**, *141*, 14298–14305.
[9] W. Liang, H. Xu, F. Carraro, N. K. Maddigan, Q. Li, S. G. Bell, D. M. Huang, A. Tarzia, M. B. Solomon, H. Amenitsch, L. Vaccari, C. J. Sumby, P. Falcaro, C. J. Doonan, *J. Am. Chem. Soc.* **2019**, *141*, 2348–2355.
[10] J. Schindelin, I. Arganda-Carreras, E. Frise, V. Kaynig, M. Longair, T. Pietzsch, S. Preibisch, C. Rueden, S. Saalfeld, B. Schmid, J.-Y. Tinevez, D. J. White, V. Hartenstein, K. Eliceiri, P. Tomancak, A. Cardona, *Nat. Methods* **2012**, *9*, 676–682.
[11] M. M. Bradford, *Anal. Biochem.* **1976**, *72*, 248–254.
[12] P. K. Smith, R. I. Krohn, G. T. Hermanson, A. K. Mallia, F. H. Gartner, M. D. Provenzano, E. K. Fujimoto, N. M. Goeke, B. J. Olson, D. C. Klenk, *Anal. Biochem.* **1985**, *150*, 76–85.
[13] J. M. Bolivar, T. Consolati, T. Mayr, B. Nidetzky, *Biotechnol. Bioeng.* **2013**, *110*, 2086–2095.
[14] I. Dib, A. Slavica, W. Riethorst, B. Nidetzky, *Biotechnol. Bioeng.* **2006**, *94*, 645–654.
[15] A. Weinhäusl, R. Griessler, A. Krebs, P. Zipper, D. Haltrich, K. D. Kulbe, B. Nidetzky, *Biochem. J.* **1997**, *326*, 773–783.
[16] S. Saheki, A. Takeda, T. Shimazu, *Anal. Biochem.* **1985**, *148*, 277–281.
[17] R. Chen, J. Yao, Q. Gu, S. Smeets, C. Baerlocher, H. Gu, D. Zhu, W. Morris, O. M. Yaghi, H. Wang, *Chem. Commun.* **2013**, *49*, 9500–9502.
[18] C. Zhong, B. Nidetzky, *Biotechnol. J.* **2020**, *15*, 1900349.
